# Radical hydroxymethylation of alkyl iodides using formaldehyde as a C1 synthon†

**DOI:** 10.1039/d1sc03083c

**Published:** 2021-07-06

**Authors:** Lewis Caiger, Conar Sinton, Timothée Constantin, James J. Douglas, Nadeem S. Sheikh, Fabio Juliá, Daniele Leonori

**Affiliations:** Department of Chemistry, University of Manchester Manchester M13 9PL UK daniele.leonori@manchester.ac.uk https://leonorigroup.com; Early Chemical Development, Pharmaceuticals Sciences, R&D, AstraZeneca Macclesfield UK; Department of Chemistry, College of Science, King Faisal University P. O. Box 400 Al-Ahsa 31982 Saudi Arabia

## Abstract

Radical hydroxymethylation using formaldehyde as a C1 synthon is challenging due to the reversible and endothermic nature of the addition process. Here we report a strategy that couples alkyl iodide building blocks with formaldehyde through the use of photocatalysis and a phosphine additive. Halogen-atom transfer (XAT) from α-aminoalkyl radicals is leveraged to convert the iodide into the corresponding open-shell species, while its following addition to formaldehyde is rendered irreversible by trapping the transient O-radical with PPh_3_. This event delivers a phosphoranyl radical that re-generates the alkyl radical and provides the hydroxymethylated product.

## Abstract 2

Halogen-atom transfer (XAT) based on phosphoranyl radical chemistry enables the hydroxymethylation of alkyl iodides with formaldehyde.

## Graphical abstract

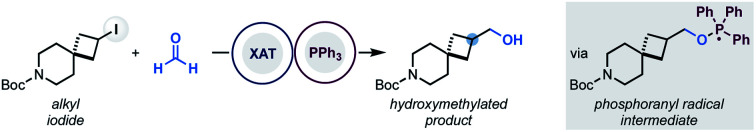

## Introduction

**Scheme 1:**
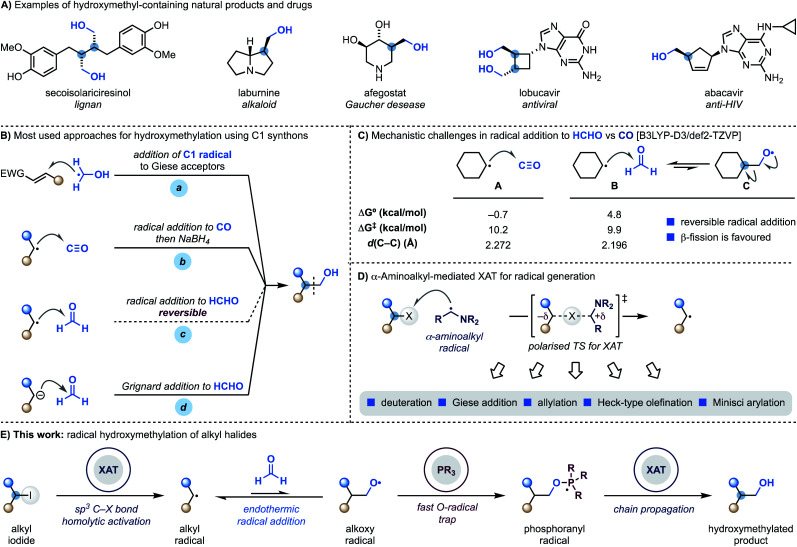
(A) Examples of natural products and therapeutic agents containing the hydroxymethyl motif. (B) Overview of the main methods to install a hydroxymethyl motif using radical chemistry. (C) Radical addition to CO is possible while addition to HCHO is difficult due to reversibility. (D) α-Aminoalkyl radicals enable XAT activation of alkyl iodides and bromides and the corresponding carbon radicals can be engaged in several transformations. (E) This work shows the utilisation of XAT and phosphoranyl radical chemistry for the direct coupling of unactivated alkyl iodides with HCHO.

Hydroxymethyl motifs are frequently encountered in the core structure of natural products and drugs and commonly used as handles for further derivatisation (Scheme 1A).^1^ This structural and practical relevance makes the development of methods able to introduce the “CH_2_OH” fragment onto advanced building blocks impactful to the discovery and development of high-value materials.^2^

Retrosynthetically, the most direct approach to achieve programmable small-molecule hydroxymethylation is through the formation of a sp^3^–sp^3^ C–C bond with an oxygenated C1 synthon.^3^ Within the realm of radical chemistry, α-hydroxymethyl radicals, which are easily generated from MeOH *via* HAT (H-atom transfer), have been successfully applied to the functionalisation of Giese^4^ and Minisci^5^ acceptors (Scheme 1B, path a). A significantly less explored avenue is the development of methodologies where C-radicals react with C1 SOMOphiles.^6^

Considering the three most atom-economic and abundant oxygenated C1 synthons – CO_2_, CO and HCHO – CO is the most used in radical strategies. Indeed, while radical addition to CO_2_ is endothermic,^7^ reaction with CO is both kinetically and thermodynamically facile and, as demonstrated by the pioneering work of Ryu, also compatible for application in radical chain propagations.^8^ This approach however, provides the aldehyde as the product of the radical process and thus requires a subsequent stoichiometric hydride reduction step (Scheme 1B, path b).^9^

The difficulties in using formaldehyde as a C1 synthon for radical hydroxymethylation (Scheme 1B, path c) come from the reversible nature of the addition process.^9*a*,10^ According to our computational studies, the reaction between cyclohexyl radicals and HCHO (**B**) is kinetically accelerated compared to the reaction with CO (**A**) but, crucially, endothermic with a later transition state character (as determined by comparison of the respective *d*(C–C) values) (Scheme 1C).^11^ This means that the back reaction, involving β-scission of the primary O-radical **C**, is a fast process which hampers reaction development. Overall, this mechanistic challenge has impacted the practical ways we conduct hydroxymethylation with HCHO, which is generally achieved using Grignard or organolithium reagents that might limit functional group compatibility (Scheme 1B, path d).^12^

We have recently demonstrated that alkyl radicals can be conveniently accessed from the corresponding halides using halogen-atom transfer (XAT) with α-aminoalkyl radicals (Scheme 1D).^13^ These open-shell species can be generated from the corresponding amines by SET (single-electron transfer) oxidation^14^ and deprotonation^15^ and display an abstracting profile similar to that of tin radicals in the homolytic activation of organic halides. This reactivity mode benefits from a polarised transition state^13,16^ with significant charge-transfer character and can be used in redox-neutral photoredox manifolds and also as an initiation mechanism in transformation based on radical-chain propagations.^13*b*^

We recently became interested in benchmarking XAT reactivity with the aim of enabling radical couplings between alkyl halides and HCHO. Here we demonstrate the realisation of this goal and report the development of a practical approach for direct radical hydroxymethylation (Scheme 1E). The process sequentially exploits XAT to generate an alkyl radical and then harnesses the ability of PPh_3_ to trap the resulting O-radical. This approach effectively renders the radical addition to HCHO an irreversible process. Crucially, the ensuing phosphoranyl radical is able to sustain a radical chain propagation based on XAT to give the hydroxymethylated product.

## Results and discussion

### Mechanistic considerations and reaction optimization

**Scheme 2:**
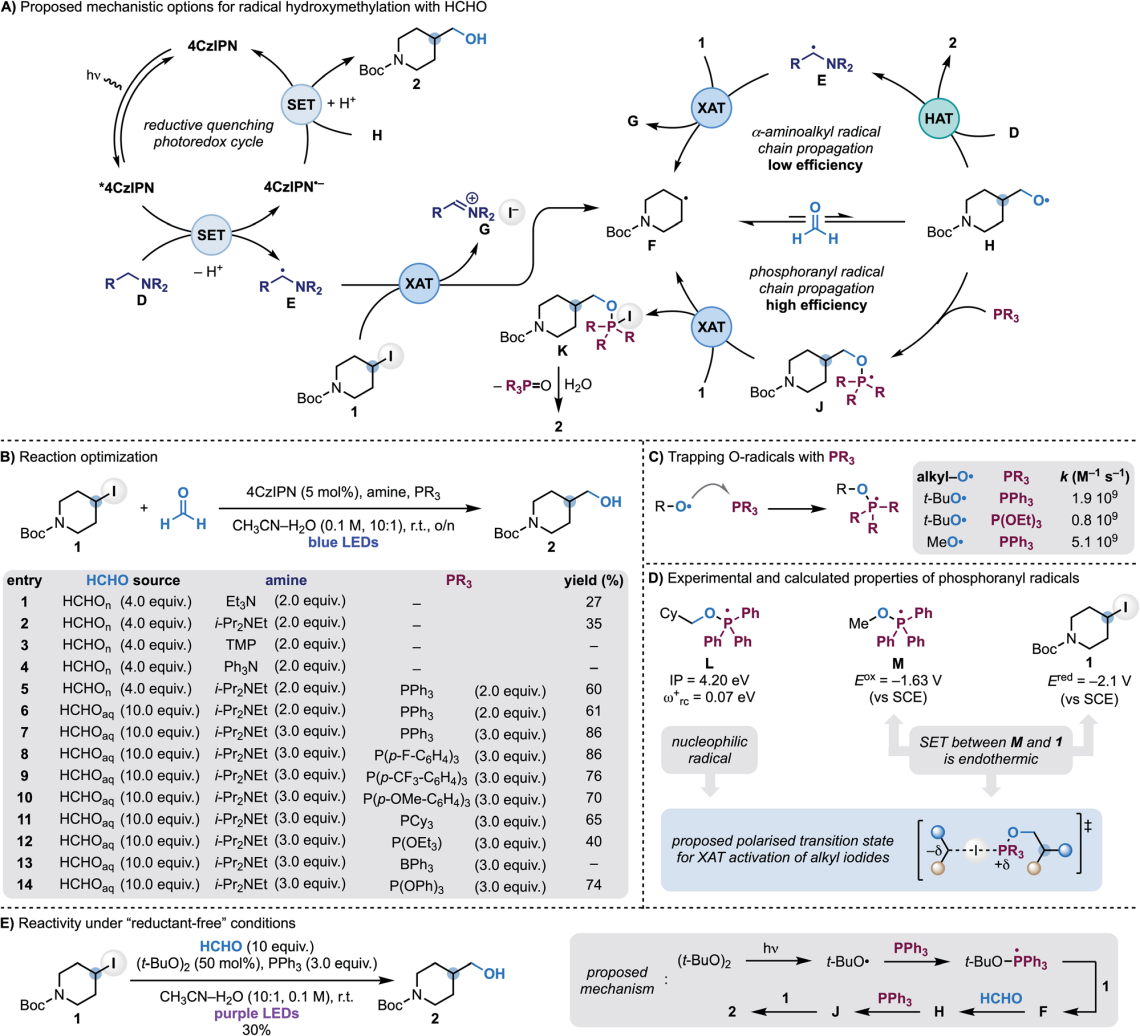
(A) Mechanistic proposal for the coupling of alkyl iodides with HCHO using XAT and phosphoranyl radical chemistry. (B) Optimisation of the radical coupling between iodide **1** and HCHO. (C) Literature rate constants for the reaction of O-radical with P(iii) compounds to give phosphoranyl radicals. (D) Experimental and calculated properties of phosphoranyl radicals suggest XAT as the alkyl iodide activation mechanism. (E) “Reductant-free” reaction for hydroxymethylation of alkyl iodides *via* phosphoranyl radicals.

In approaching the development of a radical hydroxymethylation reaction using HCHO as a C1 synthon, we initially considered the utilisation of a reductive quenching photoredox cycle (Scheme 2A). In principle, this manifold would enable the generation of the key α-aminoalkyl radical (**D** → **E**) for XAT with the alkyl iodide (*e.g.* 4-iodo-*N*-Boc-piperidine **1**). According to our previous work, this step should be facile and provide radical **F** along with the iminium iodide **G**. Addition of **F** to HCHO is reversible making a potential SET reduction (followed by protonation) of the O-radical **H** with the reduced photocatalyst challenging. However, our interest was drawn by the fact that this mechanism could potentially benefit from radical-chain reactivity where **H** undergoes polarity matched HAT (H-atom transfer) with the amine **D**. Since O-radicals undergo related HAT reactions at fast rates (∼10^8^ M^−1^ s^−1^)^17^ and the amine would be used in excess, this step should provide **2** whilst regenerating the chain-carrying α-aminoalkyl radical **E**.

We started our investigations using **1** as the iodide, 4CzIPN as the photocatalyst and paraformaldehyde in CH_3_CN–H_2_O (10 : 1) solvent under blue light irradiation at room temperature (Scheme 2B). Pleasingly, using Et_3_N as the amine, alcohol **2** was formed in moderate yield which was improved by using i-Pr_2_NEt (entries 1 and 2). In accordance with our mechanistic picture, evaluation of amines that can act as efficient electron donors for *4CzIPN but cannot lead to the formation of an α-aminoalkyl radical (by either oxidation and deprotonation or HAT) led to no product formation [compare entries 3 (TMP = tetramethylpiperidine) and 4 (Ph_3_N) with 2 (i-Pr_2_NEt)]. Unfortunately, evaluation of the other reaction parameters^11^ did not further improve the efficiency of the process which likely underscores the challenges in overcoming the reversibility of the radical addition process (**F** + HCHO ⇆ **H**).

We therefore proposed to overcome the unwanted β-fragmentation by trapping the transient O-radical **H** with an immediate reaction. Specifically, we were interested by the well-known ability of phosphines to trap O-radicals at diffusion-controlled rates leading to the corresponding phosphoranyl radicals (Scheme 2C).^18^ These open-shell intermediates can still undergo β-scission across the sp^3^ C–O bond but the feasibility of this process depends on the nature of the ensuing C-radical, and it is only efficient when tertiary or stabilised (*e.g.* benzylic) species are generated.^19^ In our case this fragmentation would lead to a primary alkyl radical, so we were hopeful that **J** would be long-lived enough to participate in a following reaction. In particular, we anticipated that **J** might be able to abstract an I-atom from **1** and therefore establish a phosphoranyl radical-based chain propagating system. This step would give the P(v) intermediate **K** (ref. 20) that could provide the targeted **2** upon hydrolysis. While XAT between phosphoranyl radicals and alkyl halides has not been reported before,^21^ we speculated that their high nucleophilic character^22^ should lead to polar effects related to those demonstrated in abstraction reactions with tin, silicon and α-aminoalkyl radicals.^13*a*^

This mechanistic proposal was validated by the addition of PPh_3_ to the reaction mixture, which immediately led to a dramatic increase in the process yield (Scheme 2B, entry 6). Further improvements were made by using formalin in place of paraformaldehyde and increasing the equivalents of both i-Pr_2_NEt and PPh_3_. Under the conditions reported in entry 7, **2** was obtained in 86% yield. Other triaryl/trialkyl phosphines were evaluated and were also successful albeit in lower yield (entries 9–12). Phosphites and triaryl boranes are also known to efficiently trap O-radicals^18^ and were evaluated. While P(OEt)_3_ resulted in significantly lower yield (entry 12) and BPh_3_ completely suppressed the reactivity (entry 13), P(OPh)_3_ gave **2** in 74% yield (entry 14).^23^ Finally, control experiments demonstrated the reaction required all components as well as continuous blue LEDs irradiation.^11^

A photoredox initiation based on a reductive quenching cycle is supported by our Stern–Volmer studies whereby the amine quenches *4CzIPN fluorescence with the largest rate constant.^11^ Obtaining evidence on the XAT reactivity of the phosphoranyl radical **J** has been more difficult since, despite considerable efforts, we have not be able to locate a transition state for this transformation. Nevertheless, we believe the strong nucleophilic character of phosphoranyl radical [determined by calculating the ionization potential (IP) and electrophilicity index (*ω*_rc_^+^) for **L**] should provide effective charge-transfer stabilisation in a XAT transition state, just like the chain initiating α-aminoalkyl radicals. Furthermore, its intermediate reductive power (determined by measuring the reduction potential of the phosphonium salt Ph_3_(MeO)P(OTf) to give **M**) supports a reaction with alkyl iodide **1** (*E*_red_ = −2.09 V *vs.* SCE) based on XAT over SET (Scheme 2D).^11^ In addition, we have been able to translate this reactivity under “reductant-free” conditions. As shown in Scheme 2E we have used the photochemical O–O bond homolysis of (*t*-BuO)_2_ to generate a *t*-BuO-containing phosphoranyl radical from which XAT from **1** and a subsequent reaction with HCHO enables formation of **2** (30% yield).^11^ Overall, we believe this reaction provides supporting evidence for the ability of phosphoranyl radicals to abstract iodine atoms from alkyl residues.

### Substrate scope

Having identified optimal conditions for the radical coupling between alkyl iodides and formaldehyde, we evaluated the scope of the process. A series of commercial iodides based on valuable N-heterocyclic systems provided access to C3-functionalised *N*-Boc-piperidine (**3**), -azepane (**4**), -pyrrolidine (**5**) and -proline (**6**) in generally high yields. The chemistry was also translated to I-containing *N*-Boc-azetidine and 4-iodo-(thio)pyran to give alcohols **7–10**. Alcohol **9** Represents an example of a chemoselective XAT process as aryl bromides are more difficult to activate using α-aminoalkyl radicals, allowing selective targeting of the sp^3^ C–I bond. The successful formation of **11** in good yield demonstrates compatibility with carbonyl functionalities that might be problematic when using reactive organometallic intermediates. The chemistry was then applied to the hydroxymethylation of unactivated 4-Ph-cyclohexyl iodide (**12**) and also used on 2- and 1-iodo-adamantane (**13** and **14**), thus demonstrating compatibility with tertiary substrates.

We also succeeded in employing this chemistry on iodinated *N*-Boc-protected cyclohexylamine (**15**) and cyclobutylamine (**16**) as well as two commercial spirocyclic building blocks (**17** and **18**) and a bicyclic derivative (**19**) in high yield.

**Scheme 3:**
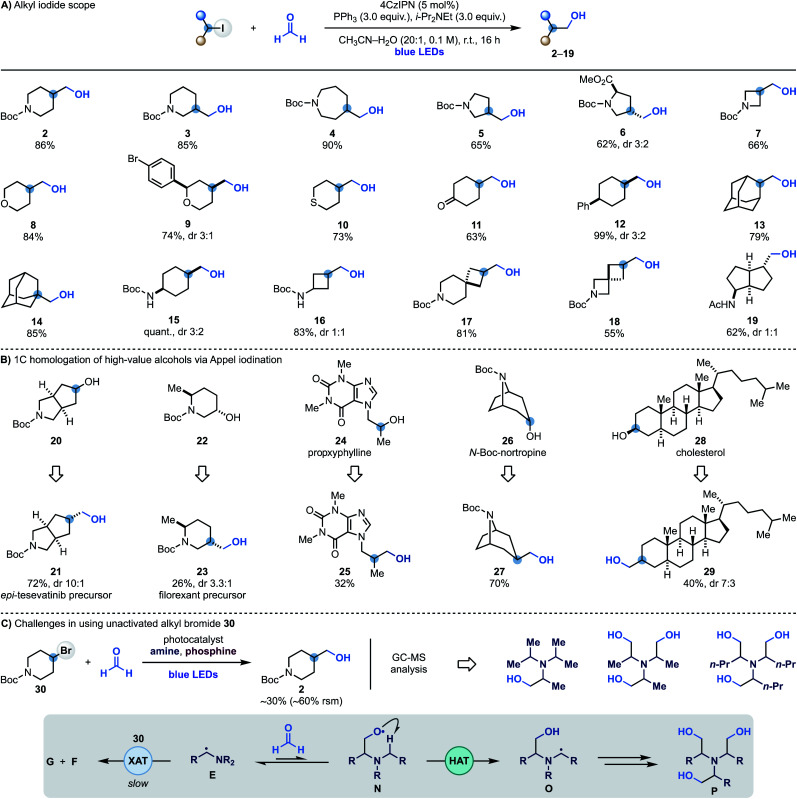
(A) Alkyl iodide scope. (B) Application of the hydroxymethylation strategy in the 1C homologation of high-value alcohols *via* Appel iodination followed by radical coupling with HCHO. (C) Difficulties in extending the hydroxymethylation reactivity to unactivated alkyl bromide **30**. rsm is remaining starting material.

To showcase the applicability of this methodology we sought to use it to achieve the one-carbon homologation of high-value alcohols using Appel iodination followed by XAT–phosphoranyl radical-mediated hydroxymethylation. As shown in Scheme 3B, the commercial N-heterocycle **20** gave **21** which can lead to the preparation of analogues of the kidney cancer treatment drug tesevatinib. Piperidine **22** provided **23**, which is a synthetic intermediate in the manufacture of the orexin antagonist filorexant. The blockbuster cardiac stimulant proxyphylline **24** could also be engaged thus broadening the functional group compatibility and providing access to the 1C-homologated drug analogue **25** in useful yield. Furthermore, subjecting the *N*-Boc protected alkaloid nortropine **26** and cholesterol **28** to this two-step sequence gave the 1C-homologation products **27** and **29**.

While α-aminoalkyl radicals can be successfully used for the homolytic activation of both alkyl iodides and bromides,^13*a*^ this hydroxymethylation strategy is at the moment synthetically useful only for the iodides. In the case of the bromides, as XAT is slower (*k*_XAT_ < 10^5^ M^−1^ s^−1^ for Cy–Br and the Et_3_N α-aminoalkyl radical),^13*a*^ other unwanted reactivities become competitive which may hamper product formation. Indeed, when we attempted the reaction with 4-bromo-*N*-Boc-piperidine **30** using various combinations of photocatalysts, amines and oxidants, we obtained **2** in up to 30% yield, recovered **30** in >60% yield and identified by mass spectrometry analysis several by-products resulting from the hydroxymethylation of the amine reagent. We believe that in these cases where XAT is slower, the nucleophilic α-aminoalkyl radical **E** can be trapped by HCHO leading to O-radical **N**. This species can either react with PPh_3_, or, in the case of linear trialkyl amines (*e.g.* Et_3_N), undergo intramolecular and polarity matched HAT from the other α-*N*-positions (**O**). This radical translocation process ultimately leads to the accumulation of polar poly-hydroxymethylated derivatives (**P**).^11^

### Hydroxymethylation of other alkyl radical precursors

**Scheme 4:**
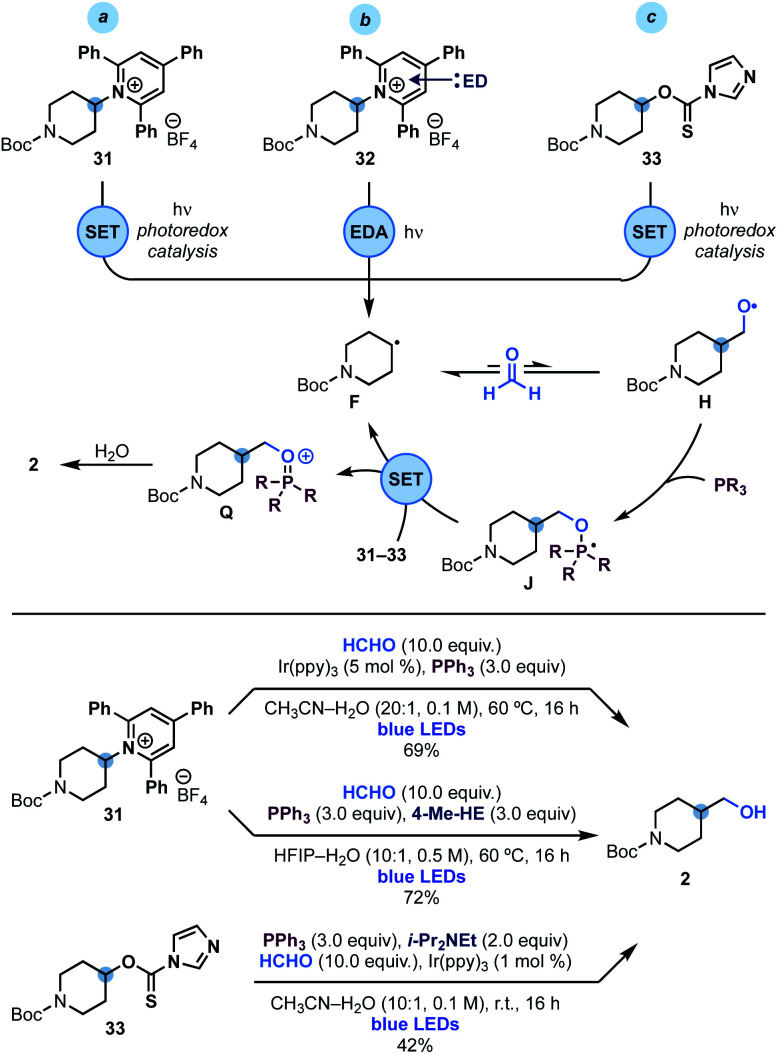
Mechanistic analysis and reaction conditions for the hydroxymethylation of Katritzky's pyridinium **31** and xanthate **33**.

Since phosphoranyl radicals are relatively good reductants (*E*^ox^ = −1.63 V *vs.* SCE, see Scheme 2D), we wondered if this strategy could be extended to the hydroxymethylation of other alkyl radical precursors based on electrophores easier to reduce than alkyl iodides. We were particularly interested by the use of Katritzky's pyridinium salts^24^ and imidazole thiocarbonyls^25^ as these species would enable the overall hydroxymethylation of amines and alcohols. As shown in Scheme 4, we succeeded in engaging pyridinium **31** (*E*_red_ = −0.94 V *vs.* SCE) in this reactivity using either photoredox or EDA (electron donor–acceptor)^26^ conditions. In the first case an oxidative quenching photoredox cycle using Ir(ppy)_3_ photocatalyst was used as the initiation mechanism for alkyl radical **F** generation. In the latter case, the supramolecular association of **31** and 4-Me-Hantzsch ester (4-Me-HE) generated an EDA complex^26^**32** that underwent photoinduced SET upon irradiation of its charge-transfer band with blue light.

Extension of this chemistry to thiocarbamate **33** proved more challenging due to the tendency of these species to undergo radical O → S rearrangement.^27^ Nevertheless, optimisation of the reaction parameters led to conditions for its implementation in useful yield using oxidative quenching of the photocatalyst Ir(ppy)_3_ as the initiation step.

In all three cases, we believe that upon alkyl radical **F** generation, reversible reaction with HCHO and fast trapping of **H** with PPh_3_, leads to phosphoranyl radical **J** that could sustain a chain propagation based on SET (instead of XAT) as the alkyl radical re-generation step. The feasibility of this step is supported by the matching redox potentials for SET between **M** (see Scheme 2D) and those of **31** and **33**.

## Conclusions

We have demonstrated that the unfavorable addition of carbon radicals to HCHO can be overcome by trapping the resulting O-radical with Ph_3_P in a fast and irreversible reaction. This leads to the generation of a phosphoranyl radical that can sustain chain propagations based on either XAT or SET. This provides versatility to the hydroxymethylation protocol that can be applied to alkyl iodides and Katritzky's salts in high yield and also thiocarbamates.

## Data availability

The data that support the findings of this study are available in the ESI and from the corresponding author upon reasonable request.

## Author contributions

FJ and DL designed the project. LC, CS, TC and FJ performed all the synthetic experiments. JJD performed some mechanistic experiments. NSS performed the computational studies. All authors analysed the results and wrote the manuscript.

## Conflicts of interest

There are no conflicts to declare.

## Supplementary Material

SC-012-D1SC03083C-s001
