# Supplementary material for: Radical hydroxymethylation of alkyl iodides using formaldehyde as a C1 synthon
Source: Chem Sci. 2021 Jul 6;12(31):10448–54. doi: 10.1039/d1sc03083c (PMC8356745; doi:10.1039/d1sc03083c)
Supplement: SC-012-D1SC03083C-s001 [file SC-012-D1SC03083C-s001.pdf]

Electronic Supplementary Material (ESI) for Chemical Science.
This journal is © The Royal Society of Chemistry 2021

Radical Hydroxymethylation of Alkyl Iodides Using Formaldehyde as a C1 Synthon

Lewis Caiger,[a] Conar Sinton,[a] Timothée Constantin,[a] James J. Douglas,[b] Nadeem S. Sheikh,[c] Fabio Juliá,[a] and Daniele Leonori*[a]

[a] *Department of Chemistry, University of Manchester, Oxford Road, Manchester M13 9PL, UK.*

[b] *Early Chemical Development, Pharmaceuticals Sciences, R&D, AstraZeneca, Macclesfield, UK*

[c] *Department of Chemistry, College of Science, King Faisal University, P. O. Box 400, Al-Ahsa 31982, Saudi Arabia.*

daniele.leonori@manchester.ac.uk

1 General Experimental Details 3

2 Starting Material Synthesis 4

3 Reaction Optimisations 11

3.1 Pictures of Reaction Set-Up 11

3.2 Hydroxymethylation of Alkyl Iodides 12

3.3 Hydroxymethylation of Katritzky's Pyridiniums 15

3.4 Hydroxymethylation of Thiocarbamates 18

4 Mechanistic Considerations 20

4.1 Proposed Mechanism for Hydroxymethylation of Alkyl Iodides 20

4.2 Stern-Volmer Quenching Studies 21

4.3 Ruling Out the Formation of Electron Donor-Acceptor (EDA) Complexes 22

4.4 Evidences Supporting XAT by the Phosphoranyl Radical 23

4.5 Quantum Yield (Φ) Determination 25

4.6 Hydroxymethylation of Alkyl Bromide 26

4.7 Cyclic Voltammetry Studies 28

5 Reaction Scope 29

6 Computational Studies 37

6.1 Computational Methods 37

6.2 Electronic Properties of Phosphoranyl Radical *38*
6.3 Reaction Energies *41*
7 NMR Spectra **49**
8 References **60**

1 General Experimental Details

All required fine chemicals were used directly without purification unless stated otherwise. All air and moisture sensitive reactions were carried out under nitrogen atmosphere using standard Schlenk manifold technique. All solvents were bought from Acros as 99.8% purity. ^{1}H and ^{13}C Nuclear Magnetic Resonance (NMR) spectra were acquired at various field strengths as indicated and were referenced to $CHCl_3$ (7.26 and 77.0 ppm for ^{1}H and ^{13}C respectively). ^{1}H NMR coupling constants are reported in Hertz and refer to apparent multiplicities and not true coupling constants. Data are reported as follows: chemical shift, integration, multiplicity (s = singlet, br s = broad singlet, d = doublet, t = triplet, q = quartet, qi = quintet, sx = sextet, sp = septet, m = multiplet, dd = doublet of doublets, etc.), proton assignment (determined by 2D NMR experiments: COSY, HSQC and HMBC) where possible. High resolution mass spectra were obtained using a JEOL JMS-700 spectrometer or a Fissions VG Trio 2000 quadrupole mass spectrometer. Spectra were obtained using electron impact ionization (EI) and chemical ionization (CI) techniques, or positive electrospray (ES). Analytical TLC: aluminium backed plates pre-coated (0.25 mm) with Merck Silica Gel 60 F254. Compounds were visualized by exposure to UV-light or by dipping the plates in permanganate ($KMnO_4$), ninhydrin or phosphomolybdic acid stains followed by heating. Flash column chromatography was performed using Merck Silica Gel 60 (40–63 μm). All mixed solvent eluents are reported as v/v solutions. Absorption and emission spectra were obtained using a Horiba Duetta spectrometer and 1 mm High Precision Cell made of quartz from Hellma Analytics. The LEDs used are Kessil PR 160 440 nm. All the reactions were conducted in CEM 10 mL glass microwave tubes.

2 Starting Material Synthesis

General Procedure for the Appel Iodination – GP1

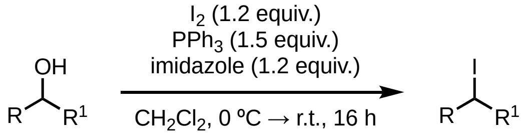

A round-bottom flask equipped with a stirring bar was charged with the alcohol (1.0 equiv.), Ph_3P (1.2 equiv.) and imidazole (1.2 equiv.). The flask was evacuated and refilled with N_2. CH_2Cl_2 (0.1 M) was added, and the reaction was cooled to 0 °C with an ice-water bath. I_2 (1.2 equiv.) was added portion-wise and then the cooling bath was removed. The reaction was stirred 16 hours at room temperature and then diluted with H_2O. The layers were separated and the aqueous layer was extracted with CH_2Cl_2 (x 3). The combined organic layers were washed with $Na_2S_2O_3$ sat., brine, dried ($MgSO_4$), filtered and evaporated. Purification by flash column chromatography on silica gel gave the products.

tert-Butyl 4-Iodoazepane-1-carboxylate (S1)

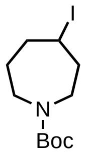

Following **GP1**, *tert*-butyl 4-hydroxyazepane-1-carboxylate (500 mg, 2.32 mmol) gave **S1** as a solid (547 mg, 72%). ^{1}H NMR (500 MHz, $CDCl_3$, rotamers) δ 4.48 (1H, bs), 3.49–3.38 (2H, m), 3.36–3.26 (2H, m), 2.24 (2H, bs), 2.15–2.04 (2H, m), 1.84 (1H, bs), 1.76–1.68 (1H, m), 1.46 (9H, s); ^{13}C NMR (126 MHz, $CDCl_3$, rotamers) δ 155.6, 79.7, 46.1, 45.7, 45.2, 41.8, 41.3, 38.8, 33.3, 28.6, 27.9, 27.7. LRMS (GCMS): Found M 325.0, $C_{11}H_{20}O_2NI$ requires 325.0539.

1-(*tert*-Butyl) 2-Methyl (2*S*,4*S*)-4-Iodopyrrolidine-1,2-dicarboxylate (S2)

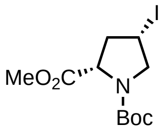

Following **GP1**, 1-(*tert*-butyl) 2-methyl (2*S*,4*R*)-4-hydroxypyrrolidine-1,2-dicarboxylate (3.55 g, 10.00 mmol) gave **S2** as a solid (2.33g, 66%). ^{1}H NMR (500 MHz, $CDCl_3$, rotamers) δ 4.28 (0.5H, t, *J* = 7.50 Hz), 4.20 (0.5H, t, *J* = 7.50 Hz), 4.10–4.00 (2H, m), 3.72 (3H, s), 3.63 (1H, dd, *J* = 10.2, 8.2), 2.85–2.82 (1H, m), 2.35–2.26 (1H, m), 1.43 (4.5H, s), 1.38 (4.5H, s); ^{13}C NMR (126 MHz, $CDCl_3$, rotamers) δ 172.1, 171.8, 153.2, 152.6, 80.7, 59.1, 58.6, 57.0, 56.6, 52.4, 52.2, 42.8, 41.9, 28.3, 28.2, 12.7, 11.9. Data in accordance with the literature.[1]

4-Iodotetrahydro-2*H*-thiopyran (S3)

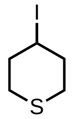

S3 is commercially available [CAS: 281204-90-8] but was prepared. Following **GP1**, tetrahydrothiopyran-4-ol (0.96 g, 8.2 mmol) gave **S3** as an oil (1.41 g, 75%). ^{1}H NMR (400 MHz, $CDCl_3$) δ 4.55–4.40 (1H, m), 2.90–2.74 (2H, m), 2.65–2.45 (2H, m), 2.40–2.15 (4H, m); ^{13}C NMR (126 MHz, $CDCl_3$, rotamers) δ 33.8, 31.0, 28.1. Data in accordance with the literature.[2]

***cis*-(4-Iodocyclohexyl)benzene (S4)**

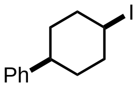

Following **GP1**, 4-phenylcyclohexan-1-ol (1.76 g, 10.00 mmol) gave **S4** as a mixture of diastereoismers as a solid (1.37 g, 48%). *cis*:*trans* 94:6. ^{1}H NMR (400 MHz, $CDCl_3$) δ 7.26–7.02 (5H, m), 4.85 (0.94H, p, *J* = 3.4 Hz), 4.12 (0.06H, tt, *J* = 12, 3.4 Hz), 2.58–2.40 (1H, m), 2.18–2.03 (2H, m), 2.02-1.87 (2H, m), 1.77–1.55 (4H, m); ^{13}C NMR (126 MHz, $CDCl_3$, rotamers) δ 146.6, 146.1, 128.4, 126.8, 126.6, 126.2, 126.1, 43.8, 42.8, 40.7, 36.5, 36.0, 30.0, 29.0. Data in accordance with the literature.[3]

***tert*-Butyl *cis*-(4-Iodocyclohexyl)carbamate (S5)**

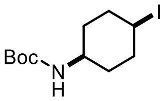

Following **GP1**, *tert*-butyl *trans*-(4-hydroxycyclohexyl)carbamate (1.00 g, 4.64 mmol) gave **S5** as a mixture of diastereomers as a solid (401 mg, 27%). ^{1}H NMR (500 MHz, $CDCl_3$) δ 4.68 (1H, bs), 4.56 (1H, bs), 3.54 (1H, bs), 2.14–1.99 (2H, m), 1.89–1.55 (6H, m), 1.43 (9H, s); ^{13}C NMR (126 MHz, $CDCl_3$, rotamers) δ 155.3, 79.4, 48.2, 35.5, 33.4, 30.2, 28.5. Data in accordance with the literature.[2]

***tert*-Butyl (3-Iodocyclobutyl)carbamate (S6)**

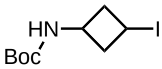

S6 is commercially available [CAS: 1389264-12-3] but was prepared. Following **GP1**, *tert*-butyl (3-hydroxycyclobutyl)carbamate (0.94 g, 5.00 mmol) gave **S6** as a solid (1.05 g, 71) as a

mixture of diastereomers. *cis:trans* 1:1. ^{1}H NMR (400 MHz, $CDCl_3$, diastereomers) δ 4.88 (0.5H, br s), 4.83 (0.5H, br s), 4.63–4.45 (0.5H, m), 4.37 (0.5H, tt, *J* = 7.7, 3.8 Hz), 4.20–4.08 (0.5H, m), 4.03 (0.5H, tt, J = 9.1, 7.3 Hz), 3.16–3.01 (1H, m), 2.78–2.66 (1H, m), 2.64–2.52 (1H, m), 2.49–2.38 (1H, m), 1.42 (4.5H, s), 1.41 (4.5H, s); ^{13}C NMR (126 MHz, $CDCl_3$, diastereomers) δ 155.0, 154.6, 46.0, 45.7, 43.8, 28.5, 11.0, 4.0; HRMS (ASAP): Found M+H$^+$ 298.0293, $C_9H_{17}O_2NI$ requires 298.0298.

***tert*-Butyl 2-Iodo-7-azaspiro[3.5]nonane-7-carboxylate (S7)**

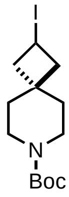

S7 is commercially available [CAS: 1638764-90-5] but was prepared. Following **GP1**, *tert*-butyl 2-hydroxy-7-azaspiro[3.5]nonane-7-carboxylate (0.96 g, 4.00 mmol) gave **S7** as a solid (0.23 g, 16%). ^{1}H NMR (400 MHz, $CDCl_3$) δ 4.49 (1H, p, *J* = 8.3 Hz), 3.35–3.29 (2H, m), 3.29–3.25 (2H, m), 2.70–2.61 (2H, m), 2.46–2.38 (2H, m), 1.72–1.63 (2H, m), 1.59–1.52 (2H, m), 1.44 (9H, s); ^{13}C NMR (126 MHz, $CDCl_3$) δ154.8, 46.3, 40.8, 39.8, 39.5, 35.2, 28.4, 9.5. Data in accordance with the literature.[3]

***tert*-Butyl 6-Iodo-2-azaspiro[3.3]heptane-2-carboxylate (S8)**

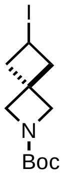

S8 is commercially available [CAS: 2059140-61-1] but was prepared. Following **GP1**, *tert*-butyl 6-hydroxy-2-azaspiro[3.3]heptane-2-carboxylate (0.85 g, 4.0 mmol) gave **S8** as a solid (0.97 g, 75%). ^{1}H NMR (400 MHz, $CDCl_3$) δ 4.29 (1H, p, *J* = 7.8 Hz), 3.94 (4H, d, *J* = 12.3 Hz), 2.96–2.87 (2H, m), 2.74–2.66 (2H, m), 1.42 (9H, s); ^{13}C NMR (126 MHz, $CDCl_3$) δ 156.1, 79.6, 47.1, 38.4, 28.4, 7.5. Data in accordance with the literature.[3]

tert-Butyl 5-Iodohexahydrocyclopenta[c]pyrrole-2(1_H_)-carboxylate (S9)

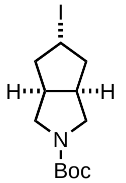

Following **GP1** *tert*-butyl-5-hydroxyhexahydrocyclopenta[c]pyrrole-2(1*H*)-carboxylate (250 mg, 1.1 mmol) gave **S9** as an oil (126 mg, 34%). R_f 0.52 [pentane:EtOAc (8.5:1.5)]; ^{1}H NMR (400 MHz, $CDCl_3$) δ 4.42 (1H, p, *J* = 5.5 Hz), 3.58–3.42 (2H, m), 3.21 (2H, dd, *J* = 11.5, 2.9 Hz), 2.90 (2H, dtt, *J* = 4.1, 8.0, 8.0 Hz), 2.44–2.26 (2H, m), 2.00 (2H, dt, *J* = 14.2, 5.4 Hz), 1.45 (9H, s); ^{13}C NMR (101 MHz, $CDCl_3$) δ 154.7, 79.6, 51.6, 46.5, 42.2, 26.2; HRMS (ESI): Found M+Na$^+$ 360.0417, $C_{12}H_{20}INO_2Na$ requires 360.0431.

tert-Butyl 5-Iodo-2-methylpiperidine-1-carboxylate (S10)

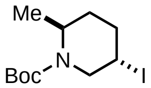

Following **GP**, *tert*-butyl 5-hydroxy-2-methylpiperidine-1-carboxylate (250 mg, 1.2 mmol) gave **S10** (78 mg, 21%) as an oil. R_f 0.4 [pentane:EtOAc 9:1]; ^{1}H NMR (400 MHz, $CDCl_3$, rotamers) δ 4.47 (0.35H, br s), 4.33 (0.35H, br s), 4.09–3.79 (1.65H, m), 3.54 (0.65H, br s), 3.36–2.97 (1H, m), 2.30–2.11 (0.8H, m), 2.09–1.94 (1.2H, m), 1.93–1.72 (1H, m), 1.64–1.53 (1H, m), 1.47 & 1.45 (9H, s), 1.25 (2H, d, J = 6.2 Hz), 1.17 (1H, d, J = 6.9 Hz); ^{13}C NMR (101 MHz, $CDCl_3$, rotamers) δ 154.7, 154.1, 80.1, 79.8, 59.7, 55.1, 33.0, 33.0, 31.4, 30.2, 28.6, 28.5, 15.5, 11.4; HRMS (ESI): Found M+Na$^+$ 348.0420 $C_{11}H_{20}INO_2Na$ requires 348.0431.

7-(2-Iodopropyl)-1,3-dimethyl-3,7-dihydro-1_H_-purine-2,6-dione (S11)

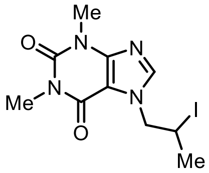

Following **GP1**, 7-(2-hydroxypropyl)-1,3-dimethyl-3,7-dihydro-1*H*-purine-2,6-dione (1.00 g, 4.20 mmol) gave **S11** as a solid (1.08 g, 74%). ^{1}H NMR (500 MHz, $CDCl_3$) δ 7.65 (1H, s), 4.61–4.54 (1H, m), 4.50 (1H, dd, *J* = 14.1, 5.0 Hz), 4.37 (1H, dd, *J* = 14.1, 9.0 Hz), 3.60 (3H, s), 3.40 (3H, s), 1.96 (3H, d, *J* = 6.9 Hz); ^{13}C NMR (126 MHz, $CDCl_3$) δ 155.4, 151.7, 149.4, 141.6, 106.7, 56.8, 30.0, 28.2, 25.4, 25.0; HRMS (APCI): Found 349.0156 $C_{10}H_{14}IN_4O_2$ requires 349.0162.

tert-Butyl 3-exo-Iodo-8-azabicyclo[3.2.1]octane-8-carboxylate (S12)

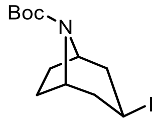

Following **GP1** *tert*-butyl *endo*-3-hydroxy-8-azabicyclo[3.2.1]octane-8-carboxylate (1.56 g, 6.93 mmol) gave **S12** as a solid (1.69 g, 73%). R_f 0.56 [petrol:EtOAc (8:2)]. ^{1}H NMR (400 MHz, $CDCl_3$, rotamers) δ 4.51 (1H, tt, *J* = 11.9, 5.7 Hz), 4.14–4.04 (1H, m), 4.04–3.92 (1H, m), 2.47–2.23 (2H, m), 2.22–2.14 (2H, m), 1.98–1.83 (2H, m), 1.71–1.57 (2H, m), 1.47 (9H, s); ^{13}C NMR (101 MHz, $CDCl_3$, rotamers) δ 150.2, 81.2, 56.2, 55.6, 45.3, 44.5, 28.5, 27.7, 27.1, 18.5; HRMS (ASAP): Found M+H$^+$ 338.0612, $C_{12}H_{21}INO_2$ requires 338.0611.

α-Cholesteryl Iodide (S13)

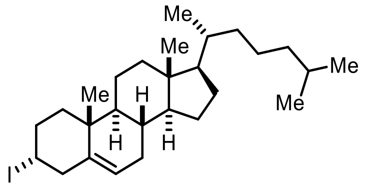

S13 is commercially available [CAS: 2930-80-5] but was prepared. Following **GP1** cholesterol (3.87 g, 10.00 mmol) gave **S13** as a solid (3.57 g, 72%). ^{1}H NMR (400 MHz, $CDCl_3$) δ 5.32 (1H, d, *J* = 5.6 Hz), 4.07–4.00 (1H, m), 2.96–2.89 (1H, m), 2.70–2.64 (1H, m), 2.30–0.85 (38H, m), 0.66 (3H, s); ^{13}C NMR (101 MHz, $CDCl_3$) δ 142.9, 121.8, 56.8, 56.2, 50.5, 46.5, 42.4, 42.0, 39.8, 39.6, 36.7, 36.6, 36.3, 35.9, 31.8, 31.7, 30.7, 28.3, 28.1, 24.4, 23.9, 22.9, 22.7, 20.9, 19.3, 18.8, 11.9. Data in accordance with the literature.[4]

2-(4-Bromophenyl)-4-iodotetrahydro-2*H*-pyran (S14)

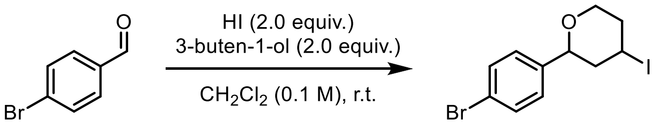

A round bottomed flask equipped with a stirring bar was charged with 4-bromobenzaldehyde (0.3 g, 1.6 mmol), CH_2Cl_2 (16 mL), 3-buten-1-ol (0.28 mL, 3.2 mmol) and HI (0.4 mL, 55 wt% solution in water, 3.2 mmol). The mixture was stirred at room temperature for 4 h when it was judged complete (TLC analysis). The mixture was diluted with H_2O (30 mL), the layers were separated, and the aqueous layer was extracted with CH_2Cl_2 (3 x 30 mL). The combined organic layers were washed with $Na_2S_2O_3$ (30 mL, 10% solution), brine (30 mL), dried ($MgSO_4$), filtered, and evaporated. Purification by flash column chromatography on silica gel gave **S14** (0.29 g, 49%) as a solid. ^{1}H NMR (400 MHz, $CDCl_3$) δ 7.46 (d, *J* = 8.5 Hz, 2H), 7.22 (d, *J* =

8.5 Hz, 2H), 4.91 (t, *J* = 3.3 Hz, 1H), 4.80 (dd, *J* = 10.6, 2.1 Hz, 1H), 4.05 (t, *J* = 5.6 Hz, 2H), 2.18 (dt, *J* = 14.7, 2.7 Hz, 1H), 1.96 (dt, *J* = 5.3, 2.7 Hz, 2H), 1.81 (ddd, *J* = 14.5, 10.6, 3.5 Hz, 1H); ^{13}C NMR (101 MHz, $CDCl_3$) δ 140.4, 131.8, 127.6, 121.8, 80.7, 69.6, 47.6, 39.6, 29.9. Data in accordance with the literature.[5]

N-(4-iodooctahydropentalen-1-yl)acetamide (S15)

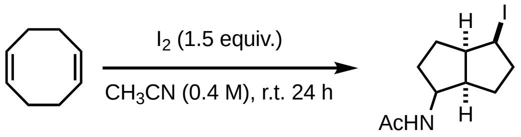

A solution of I_2 (7.6 g, 30 mmol, 1.5 equiv.) and COD (2.2 g, 20 mmol, 1.0 equiv.) in CH_3CN (50 mL, 0.4 M) was stirred at room temperature for 24 h. The mixture was diluted with aqueous NaCl (150 mL), the layers were separated and the aqueous layer was extracted with $CHCl_3$ (3 x 30 mL). The combined organic layers were washed with aqueous $Na_2S_2O_3$ (50 mL), brine (50 mL), dried ($MgSO_4$), filtered, and evaporated. Purification by flash column chromatography on silica gel gave **S15** (2.3 g, 39%) as a solid as a mixture of diastereomers. dr 1:1. ^1H NMR (400 MHz, $CDCl_3$, diastereomers) δ 5.55 (1H, s), 5.47 (1H, s), 4.42 (0.5H, dt, *J* = 8.5, 6.2 Hz), 4.35–4.22 (0.5H, m), 4.06 (0.5H, ddd, *J* = 11.8, 7.5, 5.8 Hz), 3.91 (0.5H, td, *J* = 13.9, 12.7, 6.4 Hz), 2.77 (0.5 H, dq, *J* = 17.6, 9.9, 8.9 Hz), 2.70–2.61 (0.5H, m), 2.60–2.46 (0.5H, m), 2.13 (1.5 H, m), 2.00 (1.5H, s), 1.97 (1.5H, s), 1.89–1.37 (6.5H, m), 1.35–1.14 (0.5H, m); ^{13}C NMR (101 MHz, $CDCl_3$) δ 169.8, 58.8, 53.9, 48.6, 48.0, 47.4, 44.6, 39.3, 36.5, 35.9, 33.4, 32.9, 31.8, 30.8, 30.7, 25.7, 23.4, 23.2. Data in accordance with literature.[6]

1-(1-(*tert*-Butoxycarbonyl)piperidin-4-yl)-2,4,6-triphenylpyridin-1-ium Tetrafluoroborate Salt (31)

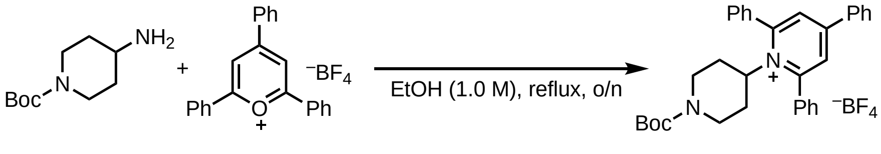

An oven-dry tube equipped with a stirring bar was charged with 2,4,6-triphenylpyrylium tetrafluoroborate (1.00 g, 2.52 mmol, 1.0 equiv.), *tert*-butyl 4-aminopiperidine-1-carboxylate (607 mg, 3.02 mmol, 1.2 equiv.) and EtOH (2.5 mL, 1.0 M). The mixture was heated under reflux overnight and then was cooled to room temperature. The mixture was diluted with Et_2O (5 mL) and vigorously stirred for 15 minutes to precipitate product. The resultant solid was filtered, washed with Et_2O (3 x 15 mL) and dried under vacuum to give **31** (993 mg, 68%) as a solid. ^1H NMR (500 MHz, $CDCl_3$) δ 7.86–7.69 (6H, m), 7.67 (2H, d, *J* = 7.2 Hz), 7.64–7.52

(6H, m), 7.52–7.46 (1H, m), 7.46–7.36 (2H, m), 4.82–4.69 (1H, m), 4.04–3.75 (2H, m), 2.27–1.95 (4H, m), 1.74–1.51 (2H, m), 1.30 (9H, s); ^{13}C NMR (101 MHz, $CDCl_3$) δ 157.2, 155.5, 154.3, 134.0, 133.8, 132.1, 131.2, 129.7, 129.4, 129.1, 128.4, 128.3, 80.2, 70.0, 44.3, 32.8, 28.3. Data in accordance with the literature.[7]

***tert*-Butyl 4-((1*H*-Imidazole-1-carbonothioyl)oxy)piperidine-1-carboxylate (32)**

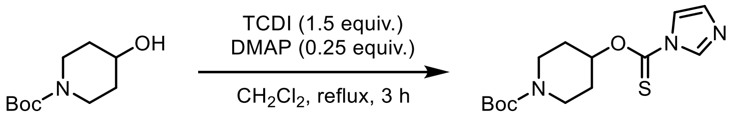

This procedure was adapted from a literature procedure:[7] a round-bottom flask equipped with a stirring bar was charged with *tert*-butyl 4-hydroxypiperidine-1-carboxylate (5.00 g, 24.8 mmol, 1.0 equiv.), DMAP (1.12 g, 9.94 mmol, 0.25 equiv.) and CH_2Cl_2 (75 mL, 0.33 M). Thiocarbonyldiimidazole (6.64 g, 37.3 mmol, 1.5 equiv.) was added and then the mixture was heated under reflux for 3 h. The mixture was cooled to r.t. and diluted with H_2O (100 mL). The layers were separated and the aqueous layer was extracted with CH_2Cl_2 (75 mL). The combined organic layers were washed with brine (100 mL), dried ($MgSO_4$), filtered and evaporated. Purification by flash column chromatography on silica gel gave **32** (6.91 g, 90%) as a solid. ^{1}H NMR (500 MHz, $CDCl_3$) δ 8.33 (1H, s), 7.61 (1H, s), 7.03 (1H, s), 5.70–5.63 (1H, m), 3.76–3.70 (2H, m), 2.08–2.02 (2H, m), 1.90–1.82 (2H, m), 1.47 (9H, s); ^{13}C NMR (101 MHz, $CDCl_3$) δ 183.1, 154.7, 136.9, 131.0, 117.9, 80.2, 79.5, 30.0, 28.5. Data in accordance with the literature.[8]

3 Reaction Optimisations

3.1 Pictures of Reaction Set-Up

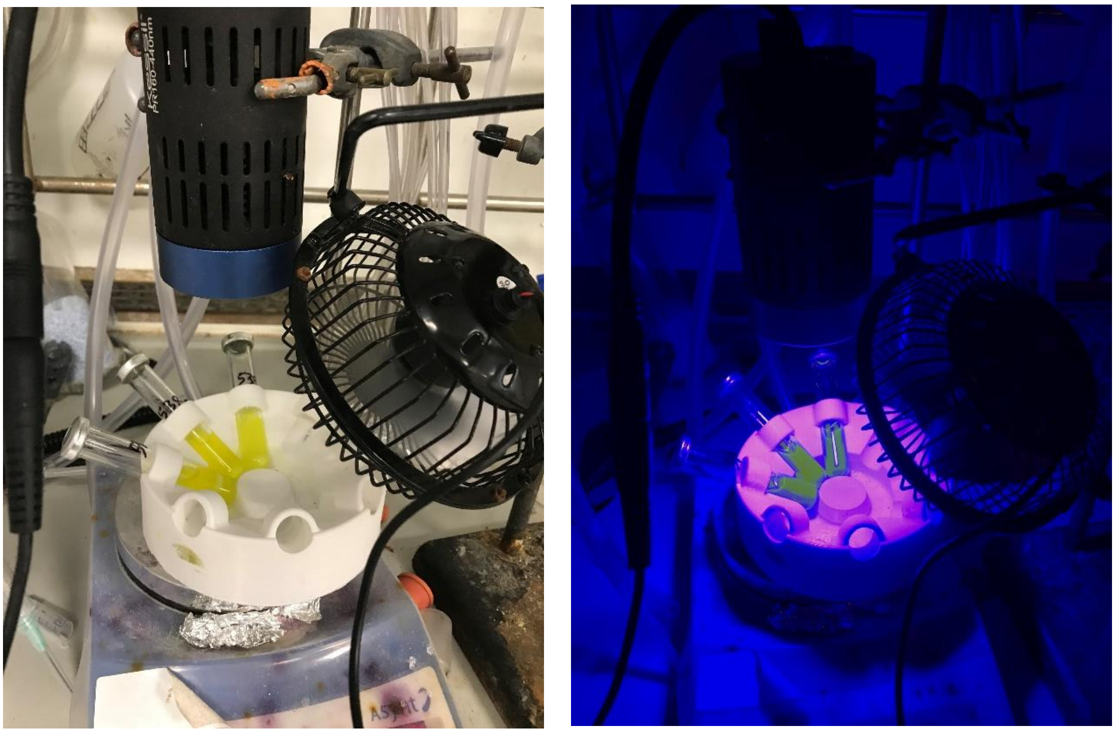

Figure S1.

3.2 Hydroxymethylation of Alkyl Iodides

General Procedure for the Hydroxymethylation of 1 – GP2

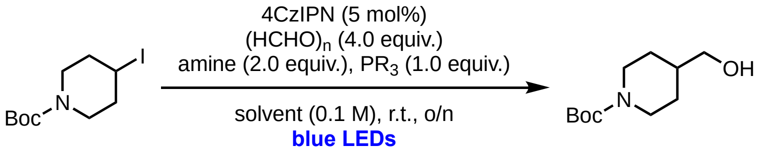

An oven-dry tube equipped with a stirring bar was charged with *tert*-butyl 4-iodopiperidine-1-carboxylate (31 mg, 0.1 mmol, 1.0 equiv.), photocatalyst (5 µmol, 5 mol%) and PR_3 (0.3 mmol, 3.0 equiv.). The tube was capped with a Supelco aluminium crimp seal with septum (PTFE/butyl), evacuated and refilled with N_2 (x 3). Dry and degassed organic solvent (1.0 mL, 0.1 M), H_2O (100 µL), H_2CO (31 µL, 0.4 mmol, 4 equiv.) and amine (0.3 mmol, 3.0 equiv.) were sequentially added. The tube was place in front of the blue LEDs (approx. 10 cm) and the lights were switched on. The mixture was stirred under continuous irradiation for 16 h whilst being cooled by a fan to give an internal temperature between 25–30 °C. The lights were switched off and the tube was opened. The mixture was diluted with brine (2 mL) and EtOAc (2 mL), then 1,3-dintrobenzene (1 mL, 0.05 M solution in EtOAc) was added as an internal standard and the mixture was vigorously shaken. The layers were separated and the aqueous layer was extracted with EtOAc (x 2). The combined organic layers were dried ($MgSO_4$), filtered and evaporated. The crude was solubilised in $CDCl_3$ and analysed by ^{1}H NMR spectroscopy.

Table S1.

Entry	Amine	PR_3	Solvent	Yield (%)
**1**	Et_3N	–	CH_3CN–H_2O (10:1)	27
**2**	*i*-Pr_2NEt	–	CH_3CN–H_2O (10:1)	35
**3**	*i*-Pr_2NH	–	CH_3CN–H_2O (10:1)	–
**4**	TMP	–	CH_3CN–H_2O (10:1)	–
**5**	PMP	–	CH_3CN–H_2O (10:1)	11
**6**	DABCO	–	CH_3CN–H_2O (10:1)	–
**7**	Ph_3N	–	CH_3CN–H_2O (10:1)	–
**8**	*i*-Pr_2NEt	PPh_3	CH_3CN–H_2O (10:1)	60
**9**[a]	*i*-Pr_2NEt	PPh_3	CH_3CN–H_2O (10:1)	61

To further improve the efficiency of the process we have used the statistical software Ellistat for DoE. We investigated the effects of varying equivalents of amine, PPh_3, HCHO and H_2O leading us to the conditions reported in Scheme S1.

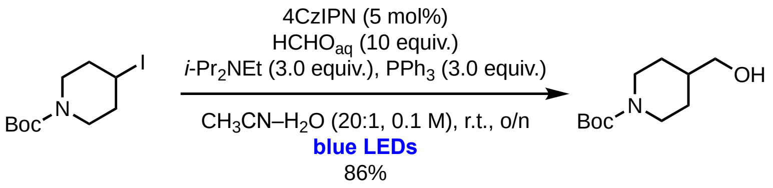

Scheme S1.

Further screening was performed using the conditions described below (Scheme S2) according to **GP2**, which is detailed in Table S2.

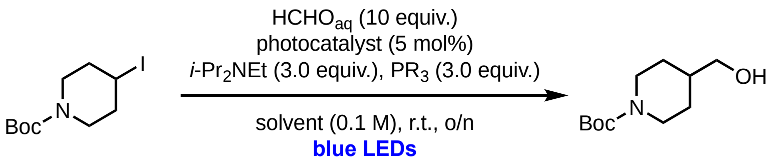

Scheme S2.

Table S2.

Entry	Photocatalyst	PR_3	Solvent	Yield (%)
**10**	4CzIPN	PPh_3	$CH_3CN–H_2O$ (20:1)	86
**11**	4CzIPN	$P(4\text{-F-}C_6H_4)_3$	$CH_3CN–H_2O$ (20:1)	78
**12**	4CzIPN	$P(4\text{-}CF_3\text{-}C_6H_4)_3$	$CH_3CN–H_2O$ (20:1)	76
**13**	4CzIPN	$P(C_6F_5)_3$	$CH_3CN–H_2O$ (20:1)	26
**14**	4CzIPN	$P(4\text{-MeO-}C_6H_4)_3$	$CH_3CN–H_2O$ (20:1)	70
**15**	4CzIPN	$P(1\text{-Nap})_3$	$CH_3CN–H_2O$ (20:1)	40
**16**	4CzIPN	PCy_3	$CH_3CN–H_2O$ (20:1)	65
**17**	4CzIPN	$P(t\text{-Bu})_3$	$CH_3CN–H_2O$ (20:1)	43
**18**	4CzIPN	$P(OEt)_3$	$CH_3CN–H_2O$ (20:1)	40
**19**	4CzIPN	$P(OPh)_3$	$CH_3CN–H_2O$ (20:1)	74
**20**	4CzIPN	Ph_3PO	$CH_3CN–H_2O$ (20:1)	48
**21**	4CzIPN	BPh_3	$CH_3CN–H_2O$ (20:1)	–
**22a**	4CzIPN	–	$CH_3CN–H_2O$ (20:1)	–

**23**	4CzIPN	PPh_3	DMF–H_2O (20:1)	58
**24**	4CzIPN	PPh_3	DMSO–H_2O (20:1)	67
**25**	4CzIPN	PPh_3	toluene–H_2O (20:1)	–
**26**	$Ru(bpy)_3Cl_2$	PPh_3	CH_3CN–H_2O (20:1)	18
**27**	Eosin Y (Na salt)	PPh_3	CH_3CN–H_2O (20:1)	36
**28**	Rhodamine 6G	PPh_3	CH_3CN–H_2O (20:1)	36
**29**	$Ir(ppy)_3$	PPh_3	CH_3CN–H_2O (20:1)	59
**30**	$Ir(ppy)_2(dtbbpy)PF_6$	PPh_3	CH_3CN–H_2O (20:1)	73
**31**	–	PPh_3	CH_3CN–H_2O (20:1)	25
**32**[a]	4CzIPN	PPh_3	CH_3CN–H_2O (20:1)	–
**33**[b]	4CzIPN	PPh_3	CH_3CN–H_2O (20:1)	–
a = no amine; b = no light				

3.3 Hydroxymethylation of Katritzky's Pyridiniums

General Procedure for the Hydroxymethylation of 31 via Photoredox Catalysis – GP3

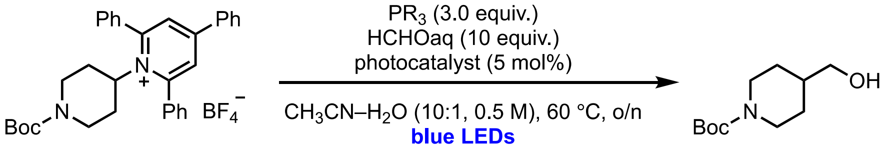

An oven-dry tube equipped with a stirring bar was charged with **31** (54 mg, 0.1 mmol, 1.0 equiv.), the photocatalyst (5 μmol, 5 mol%) and the phosphine (0.3 mmol, 3.0 equiv.). The tube was capped with a Supelco aluminium crimp seal with septum (PTFE/butyl), evacuated and refilled with N_2 (x 3). Dry and degassed CH_3CN (1.0 mL, 0.1 M), H_2O (100 µL), and H_2CO (78 µL, 1.0 mmol, 10 equiv.) were sequentially added. The tube was place in front of the blue LEDs and the lights were switched on. The reaction setup was covered in aluminium foil and the mixture was stirred under continuous irradiation for 16 h. The lights were switched off and the tube was opened. The mixture was diluted with brine (2 mL) and EtOAc (2 mL), then 1,3-dintrobenzene (1 mL, 0.05 M solution in EtOAc) was added as an internal standard and the mixture was vigorously shaken. The layers were separated, and the aqueous layer was extracted with EtOAc (x 2). The combined organic layers were dried ($MgSO_4$), filtered and evaporated. The crude was solubalised in $CDCl_3$ and analysed by ^{1}H NMR spectroscopy.

Table S3

Entry	Photocatalyst	Phosphine	Additive	Yield (%)
**1**[a]	4CzIPN	PPh_3	Na-ascorbate (2 eq.)	5
**2**[a]	$[Ir(dtbbpy)(ppy)_2]PF_6$	PPh_3	Na-ascorbate (2 eq.)	12
**3**[a]	$[Ir(dF(CF_3)ppy)_2(dtbbpy)]PF_6$	PPh_3	Na-ascorbate (2 eq.)	10
**4**[a]	$[Ru(bpy)_3]Cl_2$	PPh_3	Na-ascorbate (2 eq.)	10
**5**[a]	Rose Bengal	PPh_3	Na-ascorbate (2 eq.)	9
**6**[a]	$Ir(ppy)_3$	PPh_3	Na-ascorbate (2 eq.)	8
**7**[b]	$Ir(ppy)_3$	PPh_3	Na-ascorbate (2 eq.)	56
**8**[b]	$Ir(ppy)_3$	PPh_3	Ph_3N	59
**9**[b]	$[Ir(dtbbpy)(ppy)_2]PF_6$	PPh_3	Ph_3N	62
**10**[b]	$Ir(ppy)_3$	PPh_3	–	69
**11**[b]	$[Ir(dtbbpy)(ppy)_2]PF_6$	PPh_3	–	45
**12**[b]	$Ir(ppy)_3$	$P(4\text{-}CF_3\text{-}C_6H_4)_3$	–	10
**13**[b]	$Ir(ppy)_3$	PCy_3	–	27

**14**[b]	Ir(ppy)$_3$	HP^{t}Bu$_3$·BF$_4$	–	–
**15**[b]	Ir(ppy)$_3$	BPh$_3$	–	–
**16**[b]	Ir(ppy)$_3$	P(4-MeO-C$_6$H$_4$)$_3$	–	39
**17**[b]	Ir(ppy)$_3$	P(*o*-tol)$_3$	–	–
[a] room temperature, [b] 60°C				

General Procedure for the Hydroxymethylation of 31 via EDA – GP4

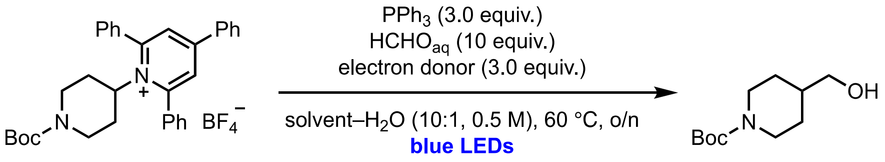

An oven-dry tube equipped with a stirring bar was charged with **31** (54 mg, 0.1 mmol, 1.0 equiv.), PPh$_3$ (79 mg, 0.3 mmol, 3.0 equiv.), and the electron donor if solid (0.3 mmol, 3 equiv.). The tube was capped with a Supelco aluminium crimp seal with septum (PTFE/butyl), evacuated and refilled with N$_2$ (x 3). Dry and degassed CH$_3$CN (1.0 mL, 0.1 M), H$_2$O (100 µL), H$_2$CO (78 µL, 1.0 mmol, 10 equiv.), and electron donor if liquid (0.3 mmol, 3.0 equiv.) were sequentially added. The tube was placed in front of the blue LEDs and the lights were switched on. The reaction setup was covered in aluminium foil and the mixture was stirred under continuous irradiation for 16 h. The lights were switched off and the tube was opened. The mixture was diluted with brine (2 mL) and EtOAc (2 mL), then 1,3-dintrobenzene (1 mL, 0.05 M solution in EtOAc) was added as an internal standard and the mixture was vigorously shaken. The layers were separated, and the aqueous layer was extracted with EtOAc (x 2). The combined organic layers were dried (MgSO$_4$), filtered and evaporated. The crude was solubilised in CDCl$_3$ and analysed by ^{1}H NMR spectroscopy.

Table S4.

Entry	Electron Donor	Solvent	Concentration	Yield (%)
**1**[a]	Et$_3$N	CH$_3$CN–H$_2$O (20:1)	0.1 M	22
**2**[a]	*i*-Pr$_2$NEt	CH$_3$CN–H$_2$O (20:1)	0.1 M	37
**3**[a]	**HE**	CH$_3$CN–H$_2$O (20:1)	0.1 M	10
**4**[a]	**HE**	CH$_3$CN–H$_2$O (20:1)	0.2 M	10
**5**	**HE**	CH$_3$CN–H$_2$O (10:1)	0.5 M	31
**6**	**HE**	DMA–H$_2$O (10:1)	0.5 M	28
**7**	**HE**	HFIP–H$_2$O (10:1)	0.5 M	41

**8**	**4-Me-HE**	HFIP–H_2O (10:1)	0.5 M	72

a = K_2CO_3 (1.5 equiv.)

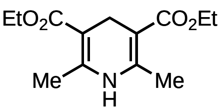

HE

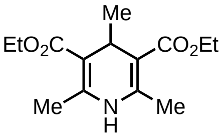

4-Me-HE

3.4 Hydroxymethylation of Thiocarbamates

General Procedure for the Hydroxymethylation of 33 – GP5

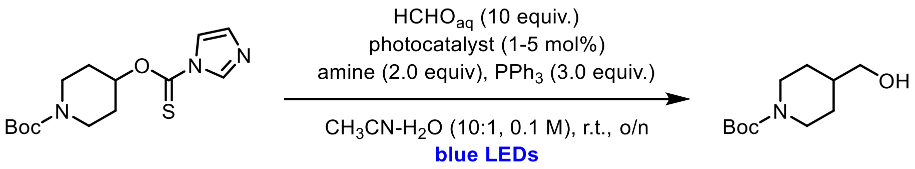

An oven-dry tube equipped with a stirring bar was charged with **33** (31 mg, 0.1 mmol, 1.0 equiv.), the photocatalyst (1-5 µmol, 1-5 mol%) and the phosphine (0.3 mmol, 3.0 equiv.). The tube was capped with a Supelco aluminium crimp seal with septum (PTFE/butyl), evacuated and refilled with N_2 (x 3). Dry and degassed CH_3CN (1.0 mL, 0.1 M), H_2O (100 µL), H_2CO (78 µL, 1.0 mmol, 10 equiv.) and the amine (0.2 mmol, 2.0 equiv.) were sequentially added. The tube was place in front of the blue LEDs (approx. 10 cm) and the lights were switched on. The mixture was stirred under continuous irradiation for 16 h whilst being cooled by a fan to give an internal temperature between 25–30 °C. The lights were switched off and the tube was opened. The mixture was diluted with brine (2 mL) and EtOAc (2 mL), then 1,3-dintrobenzene (1 mL, 0.05 M solution in EtOAc) was added as an internal standard and the mixture was vigorously shaken. The layers were separated and the aqueous layer was extracted with EtOAc (x 2). The combined organic layers were dried ($MgSO_4$), filtered and evaporated. The crude was solubilised in $CDCl_3$ and analysed by ^{1}H NMR spectroscopy.

Table S5.

Entry	Photocatalyst (mol%)	PR$_3$	Amine	Yield (%)
**1**	4CzIPN (5%)	PPh$_3$	*i*-Pr$_2$NEt	22
**2**	Ir(ppy)$_3$ (5%)	PPh$_3$	*i*-Pr$_2$NEt	29
**3**	[Ir(dtbbpy)(ppy)$_2$]PF$_6$ (5%)	PPh$_3$	*i*-Pr$_2$NEt	12
**4**	fluorescein (5%)	PPh$_3$	*i*-Pr$_2$NEt	–
**5**	eosin Y (Na salt) (5%)	PPh$_3$	*i*-Pr$_2$NEt	–
**6**	rose bengal (5%)	PPh$_3$	*i*-Pr$_2$NEt	–
**7**	rhodamine 6G (5%)	PPh$_3$	*i*-Pr$_2$NEt	–
**8**	Ir(ppy)$_3$ (1%)	PPh$_3$	*i*-Pr$_2$NEt	30
**9**	Ir(ppy)$_3$ (1%)	PPh$_3$	Et$_3$N	25
**10**	Ir(ppy)$_3$ (1%)	PPh$_3$	*i*-Pr$_2$NMe	27
**11**	Ir(ppy)$_3$ (1%)	PPh$_3$	*n*-Bu$_3$N	28

**12**	Ir(ppy)$_3$ (1%)	PPh_3	*i*-Bu$_3$N	14
**13**	Ir(ppy)$_3$ (1%)	PPh_3	PMP	13
**14**	Ir(ppy)$_3$ (1%)	PPh_3	Bn$_3$N	–
**15**[a]	Ir(ppy)$_3$ (1%)	PPh_3	*i*-Pr$_2$NEt	15
**16**	Ir(ppy)$_3$ (1%)	P(4-OMe-C$_6$H$_4$)$_3$	*i*-Pr$_2$NEt	16
**17**	Ir(ppy)$_3$ (1%)	P(4-F-C$_6$H$_4$)$_3$	*i*-Pr$_2$NEt	–
**18**[b]	Ir(ppy)$_3$ (1%)	PPh_3	*i*-Pr$_2$NEt	47
**19**	Ir(ppy)$_3$ (1%)	PPh_3	*i*-Pr$_2$NEt	22
**20**	Ir(ppy)$_3$ (1%)	PPh_3	*i*-Pr$_2$NEt	–

a = 20 mol% *i*-Pr$_2$NEt; b = reaction concentration 0.05 M

4 Mechanistic Considerations

4.1 Proposed Mechanism for Hydroxymethylation of Alkyl Iodides

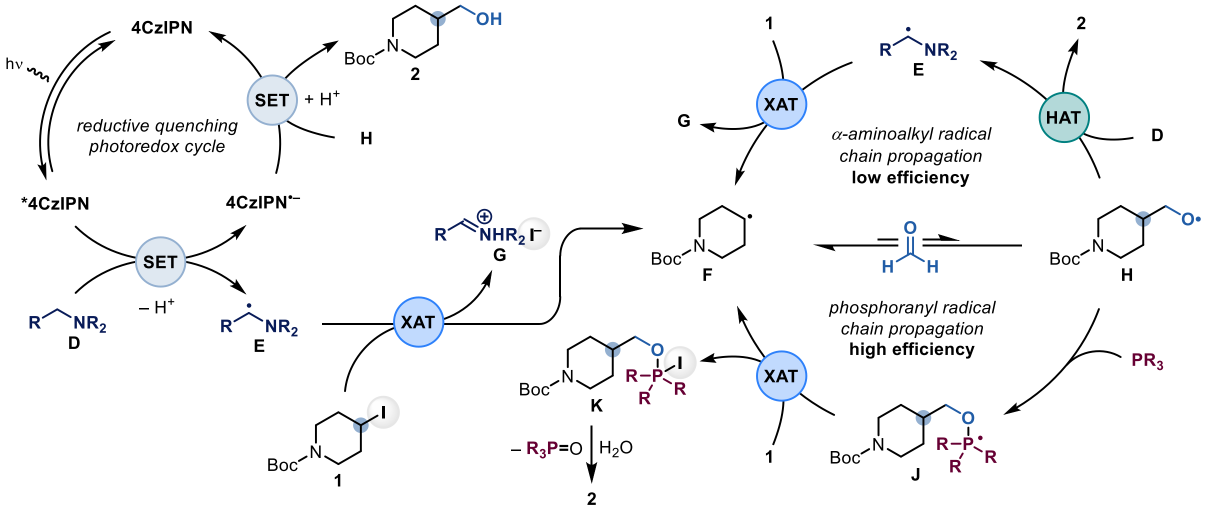

Scheme S3.

4.2 Stern-Volmer Quenching Studies

Stern-Volmer experiments were carried out monitoring the emission intensity of argon-degassed solutions of 4CzIPN (3 x 10^{-5} M solution in CH_3CN) containing variable amounts of the quencher in dry acetonitrile. The reported excited-state lifetime for 4CzIPN in CH_3CN (1.4 □s)[9] was used for k_q calculations (see Table S6). These experiments show the *i*-Pr_2NEt quenches *4CzIPN at faster rates than any other reagent (Figure S2 and Table S6).

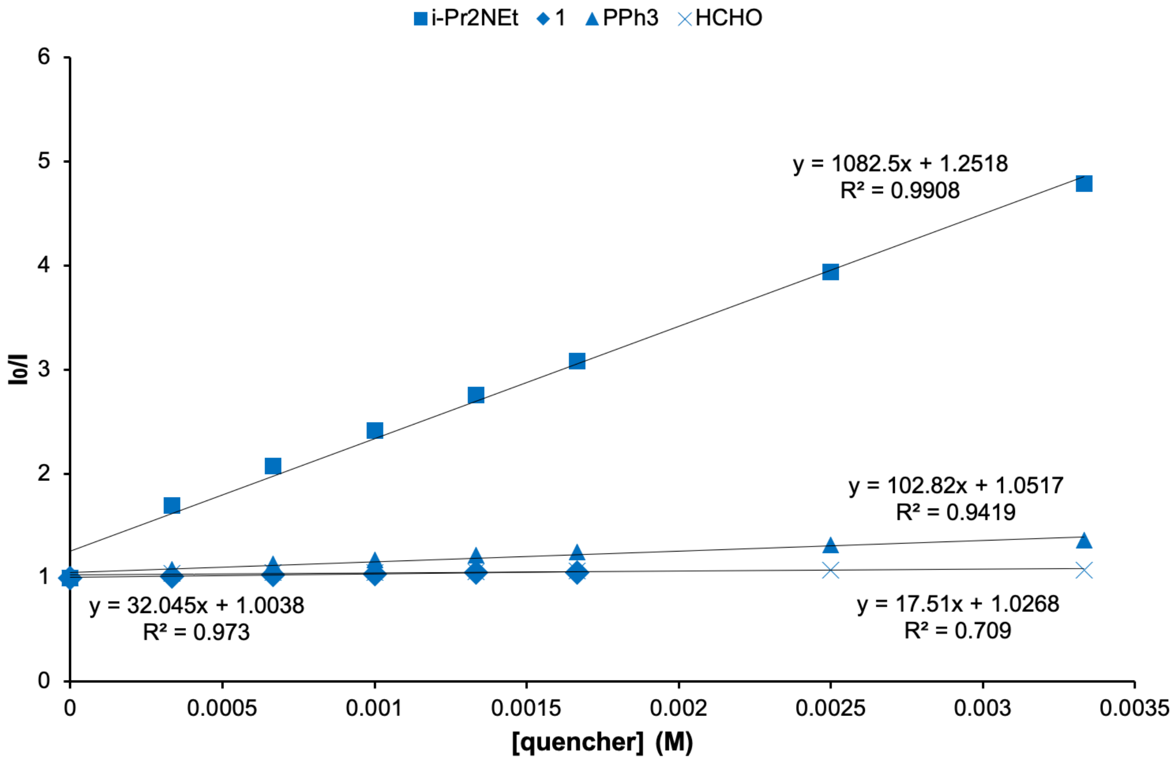

Figure S2.

Table S6.

Entry	Quencher	K_q 10^{-7} (M^{-1} s^{-1})
**1**	*i*-Pr_2NEt	156
**2**	PPh_3	14.8
**3**	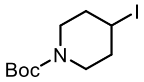	2.31
**4**	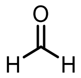	0.814

4.3 Ruling Out the Formation of Electron Donor-Acceptor (EDA) Complexes

To rule out the formation of EDA complexes between the alkyl iodide and the amine that might be absorbing in the visible region, we have performed UV/Vis absorption spectroscopy studies (Figure S3). These studies demonstrate that there is not EDA complexation between the amine and the alkyl iodide.

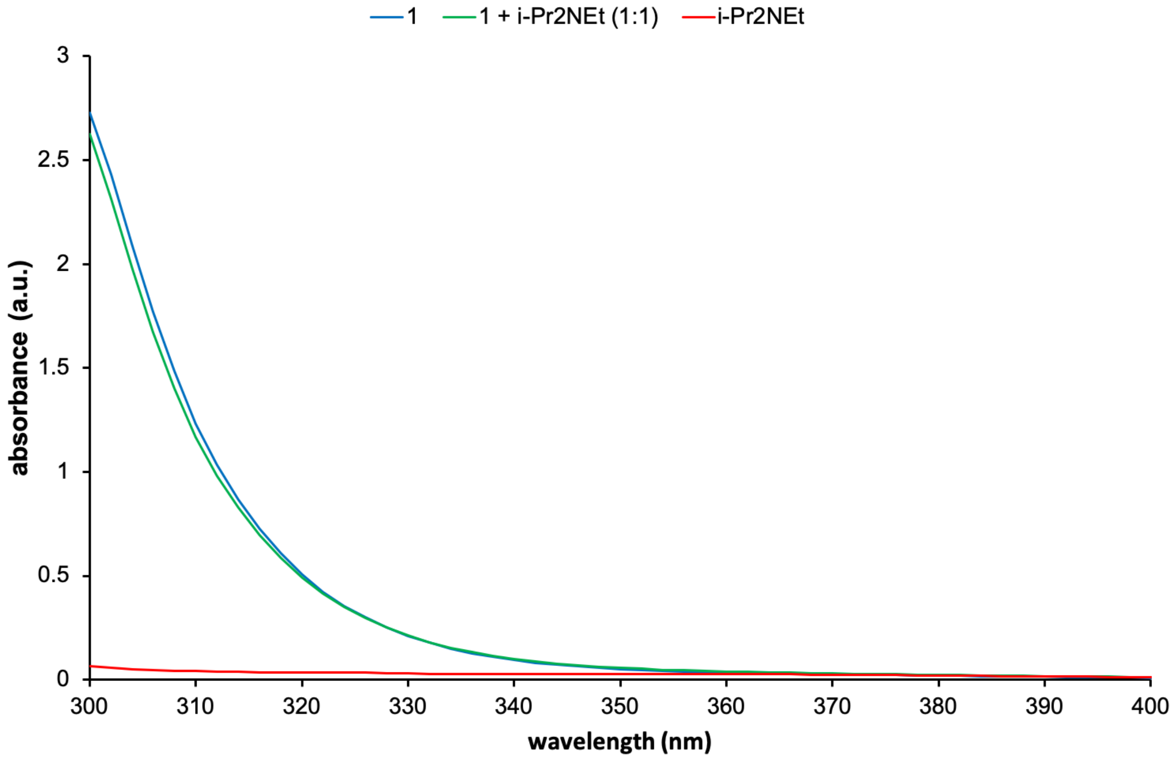

Figure S3.

4.4 Evidences Supporting XAT by the Phosphoranyl Radical

In order to obtain supporting evidences on the ability of phosphoranyl radicals to sustain a chain-propagation based on XAT, we evaluated the hydroxymethylation of **1** in the absence of amines as well as any other possible reductant (e.g. *4CzIPN or 4CzIPN$^{•-}$).

We speculated that by treatment of **1**, HCHO and PPh_3 with (*t*-BuO)$_2$, the phosphoranyl radical **X** could be obtained upon O–O bond homolysis (photochemical or thermal) and diffusion-controlled reaction of *t*-BuO• with PPh_3 (Scheme S4). **X** would then act as an initiating species for the generation of the alkyl radical **F** upon XAT on **1**. Reaction of **F** with HCHO followed by fast trap of the O-radical **H** with PPh_3 would provide the chain carrier phosphoranyl radical **J** from which product formation can occur.

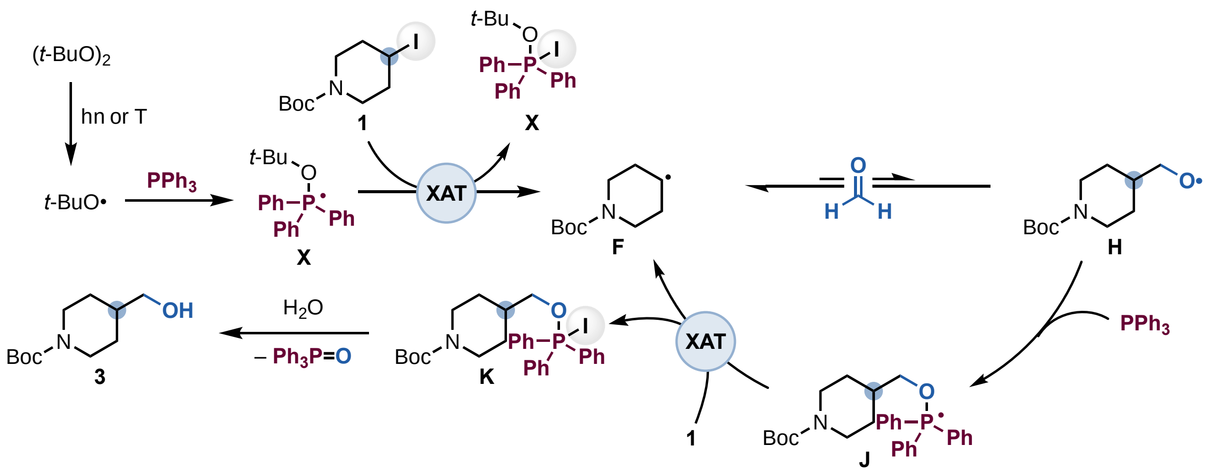

Scheme S4.

Pleasingly, irradiation of **1**, HCHO, PPh_3 and (*t*-BuO)$_2$ with purple LEDs (390 nm) led to the formation of **3** in 50% yield (Scheme 5A). Control experiments in the absence of (*t*-BuO)$_2$ resulted in full starting material recovery thus proving the homolytic activation of the sp^3 C–I bond was not taking place under these conditions (Scheme 5B).

Under these conditions we monitored the reaction by NMR to detect formation of MeI or acetone, formed from *β*-fragmentation of the phosphoranyl radical **X**. Neither of these side-products were detected, ruling out the possibility that Me• was generated and acted as XAT agent/initiator.

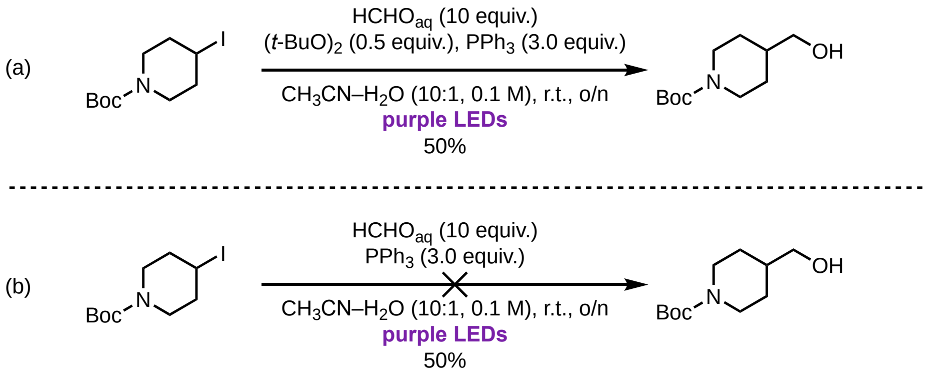

Scheme S5.

Procedure for the Peroxide Initiated Hydroxymethylation of 1

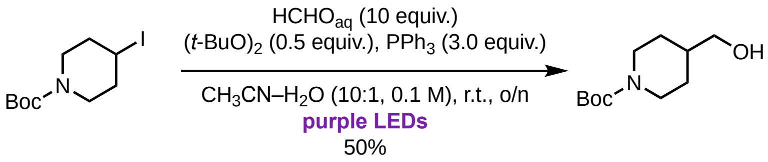

An oven-dry tube equipped with a stirring bar was charged with *tert*-butyl 4-iodopiperidine-1-carboxylate (31 mg, 0.1 mmol, 1.0 equiv.) and PPh_3 (78 mg, 0.3 mmol, 3.0 equiv.). The tube was capped with a Supelco aluminium crimp seal with septum (PTFE/butyl), evacuated and refilled with N_2 (x 3). Dry and degassed CH_3CN (1.0 mL, 0.1 M), H_2O (100 µL), H_2CO (78 µL, 1.0 mmol, 10 equiv.) and (t-BuO)$_2$ (9.2 µL, 0.05 mmol, 0.5 equiv.) were sequentially added. The tube was place in front of the purple LEDs (approx. 10 cm) and the lights were switched on. The mixture was stirred under continuous irradiation overnight whilst either being cooled by a fan or in an oil bath at 70 °C. The lights were switched off and the tube was opened. The mixture was diluted with brine (2 mL) and EtOAc (2 mL), then 1,3-dintrobenzene (1 mL, 0.05 M solution in EtOAc) was added as an internal standard and the mixture was vigorously shaken. The layers were separated and the aqueous layer was extracted with EtOAc (x 2). The combined organic layers were dried ($MgSO_4$), filtered and evaporated. The crude was solubilised in $CDCl_3$ and analysed by ^{1}H NMR spectroscopy.

4.5 Quantum Yield (Φ) Determination

The quantum yield (Φ) of the photochemical hydroxymethylation reaction of **1** was determined at 50 ºC following procedures described in literature (Scheme S6).[10] Elevated temperatures were required to accelerate the reaction for quantum yield determination, as it was not possible to record an accurate quantum yield at room temperature due to the reaction progressing slowly. The degassed reaction tube was irradiated using blue LEDs plates (λ_{max} = 444 nm) and product yield was determined by ^{1}H NMR spectroscopy analysis. The photon flux of the blue LEDs used was determined by standard ferrioxalate actinometry.[11]

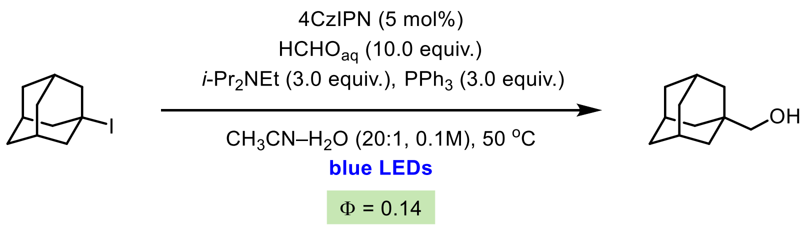

Scheme S6.

Reactions where a radical chain propogations is present are typically expected to provide a Φ > 1. In our case we have observed that the hydroxymethylation reaction displays a signifcant induction time that might account for the low Φ observed (Figure S4).

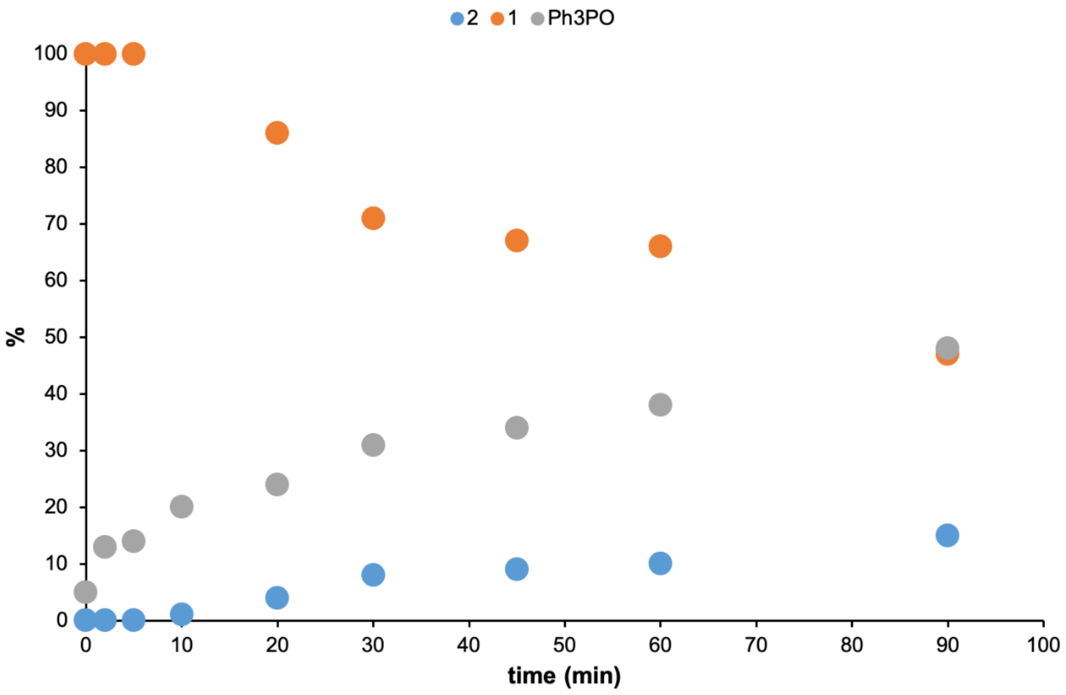

Figure S4.

4.6 Hydroxymethylation of Alkyl Bromide 30

We have evaluated the reactivity of alkyl bromide **30** under our standard conditions since α-aminoalkyl radical-mediated XAT is feasible.[12] However, the desired product **2** was obtained in low yield with remaining **30** accounting for the remaining mass balance (Scheme S7).

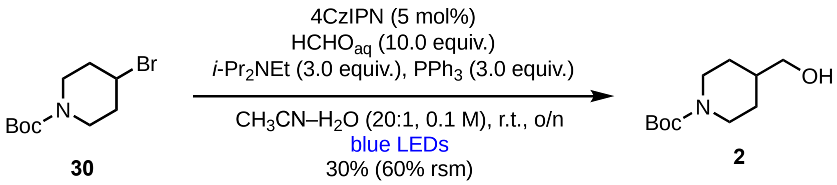

Scheme S7.

Despite considerable efforts aimed at optimising this reactivity changing all reaction parameters we did not succeed in engaging this class of derivatives in higher yield.

According to our proposed mechanism we speculated that there might have been an issue with one of the two XAT steps: either the one mediated with the α-aminoalkyl radical or the one mediated by the phosphoranyl radical. Various alkylamines were screened to evaluate their impact in the reactivity (Table S7).

Table S7.

Entry	Amine	Yield (%)
**1**	*i*-Pr$_2$NEt	25
**2**	PMP	25
**3**	*i*-Pr$_2$NMe	14
**4**	*i*-Pr$_2$NH	–
**5**	Et$_3$N	–
**6**	*n*-Bu$_3$N	–
**7**	*i*-Bu$_3$N	–
**8**	Bn$_3$N	–
**9**	Me$_3$N	–
**10**	Cy$_3$N	–
**11**	PhNMe$_2$	–
**12**	BnNPh$_2$	–

As shown in Table S7, this led to no improvement in the reaction yield. Interestingly, we detected the hydroxymethylation of some amines by mass spectrometry analysis of the reaction crudes. This suggests that in the case of the alkyl bromides were XAT is slower, the nucleophilic α-aminoalkyl radical can trap HCHO and undergo PPh_3-mediated hydroxymethylation. To obtain further evidences on this reactivity we have performed DFT studies to determine the reaction parameters for the addition step. As shown in Scheme S8, the reaction of an α-aminoalkyl radical derived from Et_3N should undergo a feasible addition to HCHO.

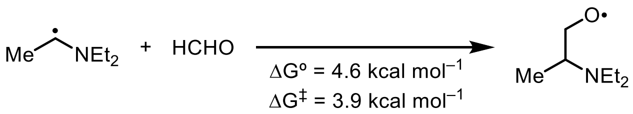

Scheme S8.

Depending on the structure of the amine, mono- or tri-hydroxymethylation was observed.

2-(Diisopropylamino)propan-1-ol (S16)

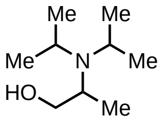

HRMS (ESI): Found M+H$^+$ 160.1691, $C_9H_{22}NO$ requires 160.1696.

2,2',2''-Nitrilotris(pentan-1-ol) (S17)

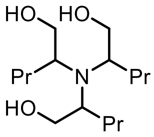

HRMS (ESI): Found M-H$^+$ 274.2393, $C_{15}H_{32}NO_3$ requires 274.2382.

2,2',2''-Nitrilotris(propan-1-ol) (S18)

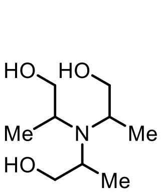

HRMS (ESI): Found M-H$^+$ 190.1451, $C_9H_{20}NO_3$ requires 190.1449.

4.7 Cyclic Voltammetry Studies

General Experimental Details

Cyclic voltammetry was conducted on an EmStat (PalmSens) potentiostat using a 3-electrode cell configuration. A glassy carbon working electrode was employed alongside a platinum wire counter electrode and a Ag/AgCl reference electrode. All the solutions were degassed by bubbling Ar prior to measurements. 10 mM solutions of the desired compounds were freshly prepared in dry acetonitrile along with 0.1 M of tetrabutylammonium hexafluorophosphate as supporting electrolyte and were examined at a scan rate of 0.1 V s^{-1}. Ferrocene ($E_{1/2}$ = +0.42 V vs SCE)[13] was added at the end of the measurements as an internal standard to determine the precise potential scale. Potential values are given versus the saturated calomel electrode (SCE). When irreversible waves were obtained the potentials were estimated at half the maximum current, as previously described by Nicewicz.[14]

Table S8.

Entry	Substrate	E_{red} (V vs SCE)
**1**	(Ph_3POMe)OTf	–1.65
**2**	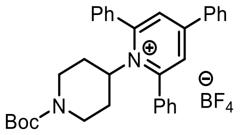	–0.89
**3**	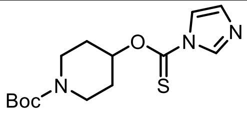	–1.71

5 Reaction Scope

General Procedure for the Hydroxymethylation of Alkyl Iodides – GP6

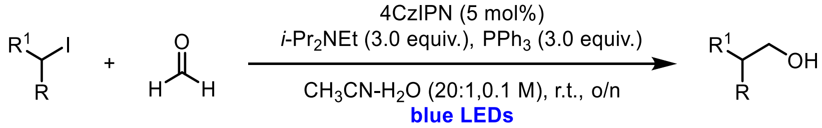

An oven-dry tube equipped with a stirring bar was charged with the alkyl iodide (0.1 mmol, 1.0 equiv.), 4CzIPN (4 mg, 5 μmol, 5 mol%) and PPh_3 (78 mg, 0.3 mmol, 3.0 equiv.). The tube was capped with a Supelco aluminium crimp seal with septum (PTFE/butyl), evacuated and refilled with N_2 (x 3). Dry and degassed CH_3CN (1.0 mL, 0.1 M), H_2O (50 µL), H_2CO (78 µL, 1.0 mmol, 10 equiv.) and (*i*-Pr)$_2$NEt (53 µL, 0.3 mmol, 3.0 equiv.) were sequentially added. The tube was place in front of the blue LEDs (approx. 10 cm) and the lights were switched on. The mixture was stirred under continuous irradiation for 16 h whilst being cooled by a fan to give an internal temperature between 25–30 °C. The lights were switched off and the tube was opened. The mixture was diluted with brine (2 mL) and EtOAc (2 mL), then 1,3-dintrobenzene (1 mL, 0.05 M solution in EtOAc) was added as an internal standard and the mixture was vigorously shaken. The layers were separated and the aqueous layer was extracted with EtOAc (x 2). The combined organic layers were dried ($MgSO_4$), filtered and evaporated. Purification by flash column chromatography on silica gel gave the products.

tert-Butyl 4-(Hydroxymethyl)piperidine-1-carboxylate (2)

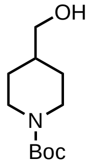

Following **GP6**, *tert*-butyl 4-iodopiperidine-1-carboxylate (31 mg, 0.1 mmol) gave **2** (19 mg, 86%) as an oil. ^{1}H NMR (500 MHz, $CDCl_3$) δ 4.14–4.09 (2H, m), 3.48 (2H, d, J = 6.3 Hz), 2.72–2.69 (2H, m), 1.74–1.67 (4H, m), 1.44 (9H, s), 1.14–1.09 (2H, m); ^{13}C NMR (126 MHz, $CDCl_3$) δ 155.0, 79.5, 67.6, 43.9, 38.9, 28.7, 28.6. Data in accordance with the literature.[15]

tert-Butyl 3-(Hydroxymethyl)piperidine-1-carboxylate (3)

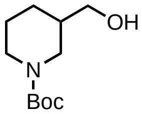

Following **GP6**, *tert*-butyl 3-iodopiperidine-1-carboxylate (31 mg, 0.1 mmol) gave **3** (18 mg, 85%) as an oil. ^{1}H NMR (500 MHz, $CDCl_3$) δ 3.78–3.50 (4H, m), 3.06–2.95 (2H, m), 1.81–

1.53 (4H, m), 1.45 (9H, s), 1.33–1.24 (1H, m); ^{13}C NMR (126 MHz, $CDCl_3$) δ 155.3, 79.5, 64.5, 46.5, 44.8, 38.1, 28.5, 26.9, 23.9. Data in accordance with literature.[16]

***tert*-Butyl 4-(Hydroxymethyl)azepane-1-carboxylate (4)**

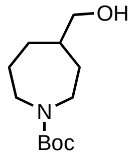

Following **GP6**, **S1** (33 mg, 0.1 mmol) gave **4** (21 mg, 90%) as an oil. R_f 0.4 [EtOAc:pentane 7:3]; ^{1}H NMR (400 MHz, $CDCl_3$) δ 3.55 (1H, dt, J = 14.2, 5.0 Hz), 3.51–3.43 (3H, m), 3.30–3.22 (2H, m), 1.93–1.81 (3H, m), 1.62–1.51 (2H, m), 1.45 (9H, s), 1.34–1.25 (1H, m), 1.17–1.10 (1H, m). ^{13}C NMR (126 MHz, $CDCl_3$) δ 155.7, 79.2, 68.2, 46.7, 45.5, 41.8, 31.4, 29.8, 28.7, 27.2. A HRMS could not be obtained for this compound.

***tert*-Butyl 3-(Hydroxymethyl)pyrrolidine-1-carboxylate (5)**

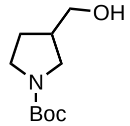

Following **GP6**, *tert*-butyl 3-iodopyrrolidine-1-carboxylate (30 mg, 0.1 mmol) gave **5** (13 mg, 65%) as an oil. ^{1}H NMR (500 MHz, $CDCl_3$) δ 3.65–3.58 (2H, m), 3.52–3.35 (2H, m), 3.34–3.22 (1H, m), 3.10–3.04 (1H, m), 2.65 (1H, s), 2.42–2.32 (1H, m), 2.01–1.85 (1H, m), 1.73–1.56 (1H, m), 1.42 (9H, s); ^{13}C NMR (126 MHz, $CDCl_3$) δ 154.6, 79.0, 63.7, 48.6, 48.1, 45.3, 44.9, 41.1, 40.3, 28.3. Data in accordance with the literature.[17]

1-(*tert*-Butyl) 2-Methyl 4-(hydroxymethyl)pyrrolidine-1,2-dicarboxylate (6)

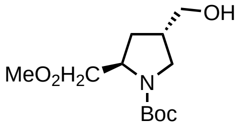

Following **GP6**, **S2** (36 mg, 0.1 mmol) gave **6** (16 mg, 62%) as an oil as a mixture of diastereomers. *trans*:*cis* 1.5:1. ^{1}H NMR (500 MHz, $CDCl_3$,) δ 4.39 (0.4H, J = 8.6, 2.8 Hz), 4.29 (0.6H, dd, J = 7.5, 4.9 Hz), 3.70–3.60 (3H, m), 3.25 (0.6H, dd, J = 10.4, 7.3 Hz), 3.19 (0.4H, dd, J = 10.4, 8.0 Hz), 2.67–2.47 (1H, m), 2.17–1.59 (2H, m), 1.46 (3.6H, s), 1.41 (5.4H, s); ^{13}C NMR (126 MHz, $CDCl_3$) δ 173.6, 173.5, 154.6, 154.0, 80.2, 80.1, 63.7, 63.5, 59.0, 58.7, 52.2, 52.0, 49.1, 48.7, 39.8, 39.0, 33.0, 32.3, 28.4, 28.3. Data in accordance with literature.[18]

***tert*-Butyl 3-(Hydroxymethyl)azetidine-1-carboxylate (7)**

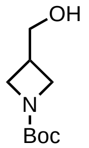

Following **GP6**, *tert*-butyl 3-iodoazetidine-1-carboxylate (28 mg, 0.1 mmol) gave **7** (12 mg, 66%) as an oil. ^{1}H NMR (500 MHz, $CDCl_3$) δ 3.99 (2H, t, *J* = 8.5 Hz), 3.78 (2H, d, *J* = 6.6 Hz), 3.68 (2H, dd, *J* = 8.8, 5.2 Hz), 2.74–2.67 (1H, m), 1.43 (9H, s); ^{13}C NMR (126 MHz, $CDCl_3$) δ 156.6, 79.6, 64.8, 30.6, 28.5. Data in accordance with the literature.[19]

(Tetrahydro-2*H*-pyran-4-yl)methanol (8)

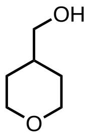

Following **GP6**, 4-iodotetrahydro-2*H*-pyran (21 mg, 0.1 mmol) gave **8** (10 mg, 84%) as an oil. ^{1}H NMR (500 MHz, $CDCl_3$) δ 4.00 (2H, dd, *J* = 11.7, 4.6 Hz), 3.51 (2H, d, *J* = 6.4 Hz), 3.41 (2H, td, *J* = 11.7, 2.1 Hz), 1.81–1.71 (1H, m), 1.68–1.62 (3H, m), 1.37 (1H, dd, *J* = 12.1, 4.5 Hz), 1.31 (1H, dd, *J* = 12.1, 4.5 Hz); ^{13}C NMR (126 MHz, $CDCl_3$) δ 67.9, 67.7, 37.7, 29.4. Data in accordance with literature.[20]

(2-(4-Bromophenyl)tetrahydro-2*H*-pyran-4-yl)methanol (9)

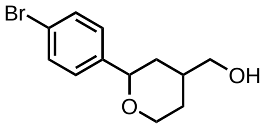

Following **GP6**, **S14** (37 mg, 0.1 mmol) gave **9** (20 mg, 74%) as an oil as a mixture of diastereomers. *cis*:*trans* 3:1. R_f 0.41 [EtOAc:Pentane 1:1; ^{1}H NMR (400 MHz, $CDCl_3$) δ 7.54–7.35 (2H, m), 7.26–7.16 (2H, m), 4.61 (0.25H, dd, *J* = 9.0, 3.4 Hz), 4.31 (0.75H, dd, *J* = 11.2, 2.1 Hz), 4.20 (0.75H, ddd, *J* = 11.5, 4.7, 1.6 Hz), 3.93 (0.25H, ddd, *J* = 10.9, 6.3, 4.5 Hz), 3.81–3.72 (0.5H, m), 3.62 (0.75H, ddd, *J* = 12.5, 11.5, 2.3 Hz), 3.58–3.43 (1.5H, m), 2.06 (0.25H, tt, *J* = 7.4, 4.8 Hz), 1.99–1.87 (1.5H, m), 1.87–1.77 (0.5H, m), 1.71 (0.75H, ddq, *J* = 13.3, 3.9, 2.0 Hz), 1.44–1.35 (0.75H, m), 1.35–1.17 (1.5H, m); ^{13}C NMR (126 MHz, $CDCl_3$, diastereomers) δ 142.14, 141.54, 131.60, 131.55, 131.54, 128.01, 127.66, 121.27, 121.14, 78.83, 73.85, 68.22, 67.92, 64.63, 63.38, 38.52, 37.26, 33.59, 33.53, 28.99, 27.24.

(Tetrahydro-2*H*-thiopyran-4-yl)methanol (10)

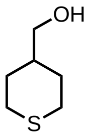

Following **GP6**, **S3** (23 mg, 0.1 mmol) gave **10** (10 mg ,73%) as an oil. ^{1}H NMR (500 MHz, $CDCl_3$) δ 3.47 (2H, d, *J* = 6.4 Hz), 2.70 (2H, ddd, *J* = 14.3, 11.9, 2.6 Hz), 2.64–2.58 (2H, m), 2.07 (2H, dd, *J* = 13.5, 3.5 Hz), 1.59 (1H, br s), 1.57–1.48 (1H, m), 1.39 (1H, dtd, *J* = 13.1, 11.8, 3.5 Hz); ^{13}C NMR (126 MHz, $CDCl_3$) δ 68.4, 40.2, 30.8, 28.3. Data in accordance with literature.[21]

4-(Hydroxymethyl)cyclohexan-1-one (11)

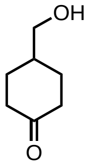

Following **GP6**, 4-iodocyclohexan-1-one (22 mg, 0.1 mmol) gave **11** (8 mg, 63%) as an oil. ^{1}H NMR (500 MHz, $CDCl_3$) δ 3.58 (2H, d, *J* = 6.6 Hz), 2.48–2.34 (4H, m), 2.13–2.08 (2H, m), 2.06–1.89 (1H, m), 1.45 (2H, dq, *J* = 12.1, 5.5 Hz); ^{13}C NMR (126 MHz, $CDCl_3$) δ 212.9, 66.5, 40.4, 38.5, 29.1. Data in accordance with literature.[22]

(4-Phenylcyclohexyl)methanol (12)

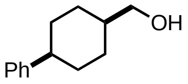

Following **GP6**, **S4** (29 mg, 0.1 mmol) gave **12** (19 mg, quant.) as an oil as a mixture of diastereomers. *cis*:*trans* 1.5:1. ^{1}H NMR (500 MHz, $CDCl_3$, diastereomers) δ 7.31–7.28 (2H, m), 7.24–7.17 (3H, m), 3.72–3.71 (1.2H, d, *J* = 7.5 Hz), 3.53–3.51 (0.8H, d, *J* = 6.4 Hz), 2.64–2.60 (0.6H, m), 2.52–2.46 (0.4H, m), 2.03–1.33 (8.2H, m), 1.27–1.04 (0.8H, m); ^{13}C NMR (126 MHz, $CDCl_3$, diastereomers) δ 147.4, 147.1, 128.3, 128.2, 126.9, 126.7, 125.8, 68.2, 64.2, 44.4, 43.3, 40.0, 35.8, 33.7, 29.8, 29.1, 26.9. Data in accordance with literature.[23]

(Adamantan-2-yl)methanol (13)

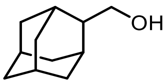

Following **GP6**, 2-iodoadamantane (26 mg, 0.1 mmol) gave **13** (13 mg, 79%) as an oil. ^{1}H NMR (500 MHz, $CDCl_3$) δ 3.74 (2H, d, *J* = 7.1 Hz), 1.94–1.90 (1H, m), 1.89–1.84 (4H, m),

1.83–1.79 (3H, m), 1.79–1.77 (1H, m), 1.75–1.72 (2H, m), 1.57 (1H, br s), 1.55 (3H, br s); ^{13}C NMR (126 MHz, $CDCl_3$) δ 65.3, 47.3, 39.1, 38.2, 31.9, 29.2, 28.4, 27.9. Data in accordance with literature.[21]

(Adamantan-1-yl)methanol (14)

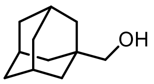

Following **GP6**, 1-iodoadamantane (26 mg, 0.1 mmol) gave **14** (14 mg, 85%) as an oil. ^{1}H NMR (500 MHz, $CDCl_3$) δ 3.20 (2H, s), 2.01–1.95 (3H, m), 1.76–1.69 (3H, m), 1.67–1.60 (3H, m), 1.54–1.46 (6H, m), 1.32 (1H, br s); ^{13}C NMR (126 MHz, $CDCl_3$) δ 73.9, 39.1, 37.3, 34.6, 28.3. Data in accordance with literature.[24]

***tert*-Butyl (4-(hydroxymethyl)cyclohexyl)carbamate (15)**

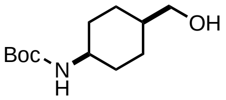

Following **GP6**, **S5** (33 mg, 0.1 mmol) gave **15** (23 mg, quant.) as an oil as a mixture of diastereomers. *cis*:*trans* 1.5:1. ^{1}H NMR (500 MHz, $CDCl_3$) δ 4.40 (1H, s), 3.45 (2H, d, J = 6.3 Hz), 3.38 (1H, s), 2.05–2.02 (2H, m), 1.84–1.81 (2H, m), 1.44 (10H, s), 1.17–1.01 (4H, m). ^{13}C NMR (101 MHz, $CDCl_3$) δ 155.4, 79.3, 68.2, 67.3, 50.1, 46.5, 39.8, 38.8, 33.1, 29.8, 29.7, 28.6, 28.6, 24.4. Data in accordance with literature.[25]

***tert*-Butyl (3-(Hydroxymethyl)cyclobutyl)carbamate (16)**

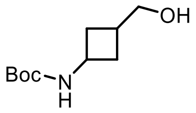

Following **GP6**, **S6** (22 mg, 0.1 mmol) gave **16** (12 mg, 60%) as an oil as a mixture of diastereomers. *cis:trans* 1:1. ^{1}H NMR (400 MHz, $CDCl_3$, diastereomers) δ 4.70 (1H, br. s), 4.17 (0.5H, br. s), 4.01 (0.5H, s), 3.67 (1H, d, J = 7.3 Hz), 3.57 (1H, d, J = 5.7 Hz), 2.48–2.39 (1H, m), 2.37–2.27 (2H, m), 2.05–1.93 (2H, m), 1.75–1.51 (1H, m), 1.66-1.54 (1H, m) 1.43 (9H, s). ^{13}C NMR (101 MHz, $CDCl_3$, diastereomers) δ 155.09, 154.86, 79.71, 79.49, 66.59, 66.30, 44.21, 42.40, 33.65, 32.71, 30.80, 30.24, 28.54. Data in accordance with literature.[26]

***tert*-Butyl 2-(Hydroxymethyl)-7-azaspiro[3.5]nonane-7-carboxylate (17)**

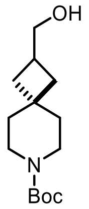

Following **GP6**, **S7** (35 mg, 0.1 mmol) gave **17** (21 mg, 81%) as an oil. R_f 0.35 [EtOAc:Pentane 1:1]; ^{1}H NMR (400 MHz, $CDCl_3$) δ 3.60 (2H, d, *J* = 6.6 Hz), 3.40–3.31 (2H, m), 3.29–3.21 (2H, m), 2.54–2.40 (1H, m), 1.95–1.85 (2H, m), 1.60–1.48 (6H, m), 1.44 (9H, s); ^{13}C NMR (101 MHz, $CDCl_3$) δ 155.2, 79.4, 68.0, 40.6, 39.3, 36.8, 34.3, 30.6, 28.6. HRMS (ESI): Found M+Na$^+$ 278.1718, $C_{14}H_{25}O_3NNa$ requires 278.1713.

***tert*-Butyl 6-(Hydroxymethyl)-2-azaspiro[3.3]heptane-2-carboxylate (18)**

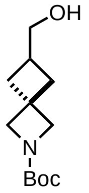

Following **GP6**, **S8** (32 mg, 0.1 mmol) gave **18** (12 mg, 55%) as an oil. ^{1}H NMR (500 MHz, $CDCl_3$) δ 3.92 (2H, s), 3.82 (2H, s), 3.56 (2H, d, *J* = 6.4 Hz), 2.35 (1H, hept, *J* = 7.1 Hz), 2.26–2.22 (2H, m), 1.96–1.92 (2H, m), 1.42 (9H, s); ^{13}C NMR (126 MHz, $CDCl_3$) δ 156.4, 79.4, 66.8, 62.4, 61.5, 35.4, 34.5, 31.5, 28.5; HRMS (ESI): Found M+Na$^+$ 250.1402, $C_{12}H_{21}O_3NNa$ requires 250.1414.

***N*-(4-(Hydroxymethyl)octahydropentalen-1-yl)acetamide (19)**

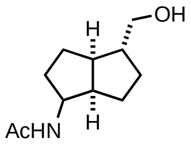

Following **GP6**, **S15** (29 mg, 0.1 mmol) gave **19** (12 mg, 62%) as an oil as a mixture of diastereomers. dr 1:1. R_f 0.25 [CH_2Cl_2:MeOH 9:1]. ^{1}H NMR (400 MHz, $CDCl_3$, diastereomers) δ 5.61 (2H, bs), 4.16–4.02 (1H, m), 3.88 (1H, dtd, *J* = 7.6, 5.6, 3.7 Hz), 3.67–3.45 (4H, m), 2.74–2.63 (1H, p, *J* = 7.8 Hz), 2.20 (1H, tt, *J* = 5.8, 3.0 Hz), 2.14–2.07 (1H, m), 1.96 (3H, s), 1.94 (3H, s), 1.84–1.17 (19H, m); ^{13}C NMR (126 MHz, $CDCl_3$, diastereomers) δ 170.0, 169.8, 66.7, 66.4, 57.4, 53.3, 51.9, 51.1, 50.3, 45.2, 45.1, 44.8, 33.0, 30.9, 30.9, 30.7, 30.0, 29.9, 29.3, 27.3, 23.7, 23.6.

tert-Butyl 5-(Hydroxymethyl)hexahydrocyclopenta[c]pyrrole-2(1H)-carboxylate (21)

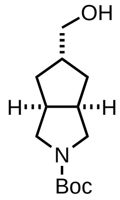

Following **GP6**, **S9** (34 mg, 0.1 mmol) gave **21** (17 mg, 72%) as an oil as a mixture of diastereomers. dr 10:1. ^{1}H NMR (400 MHz, $CDCl_3$) δ 3.53 (4H, d, *J* = 6.8 Hz), 3.13 (2H, m), 2.70 (2H, q, *J* = 3.9 Hz), 2.33 (1H, dq, *J* = 15.2, 7.8 Hz), 1.71–1.52 (4H, m), 1.45 (9H, s); ^{13}C NMR (101 MHz, $CDCl_3$) δ 154.7, 79.2, 66.8, 44.8, 41.3, 35.2, 29.9, 28.7. Data in accordance with the literature.[27]

tert-Butyl (2S,5S)-5-(Hydroxymethyl)-2-methylpiperidine-1-carboxylate (23)

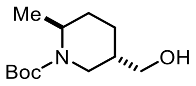

Following **GP6**, **S10** (33 mg, 0.1 mmol) gave **23** (6 mg, 26%) as an oil as a mixture of diastereomers. *trans*:*cis* 3.3:1. ^{1}H NMR (500 MHz, $CDCl_3$, diastereomers) δ 4.32 (0.22H, dd, *J* = 12.6, 3.9 Hz), 4.21 (0.78H, tt, *J* = 10.3, 5.1 Hz), 3.97 (0.22H, d, *J* = 12.8 Hz), 3.88 (0.78H, dd, *J* = 14.3, 1.3 Hz), 3.53–3.42 (2H, m), 2.95 (0.78H, dd, *J* = 14.3, 3.5 Hz), 2.47 (0.22H, t, *J* = 12.4 Hz) 2.05–1.45 (4H, m), 1.40 (9H, s), 1.12 (2.34H, d, *J* = 6.9 Hz), 1.04 (0.66H, d, *J* = 6.9 Hz); ^{13}C NMR (126 MHz, $CDCl_3$, diastereomers) δ 156.1, 79.9, 79.4, 66.2, 62.2, 47.3, 39.3, 38.1, 37.3, 35.8, 32.1, 29.9, 28.6, 26.0, 22.8, 22.1, 20.5, 16.8, 14.3. Data in accordance with literature.[28]

7-(3-Hydroxy-2-methylpropyl)-1,3-dimethyl-3,7-dihydro-1*H*-purine-2,6-dione (25)

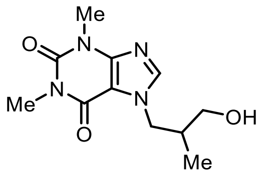

Following **GP6**, **S11** (35 mg, 0.1 mmol) gave **25** (8.0 mg, 32%) as an oil. ^{1}H NMR (500 MHz, $CDCl_3$) δ 7.59 (1H, s), 4.38 (1H, dd, *J* = 13.9, 6.9 Hz), 4.26 (1H, dd, *J* = 13.9, 5.4 Hz), 3.60 (3H, s), 3.50 (1H, dd, *J* = 11.5, 3.8 Hz), 3.41 (3H, s), 3.38 (1H, dd, *J* = 11.6, 6.5 Hz), 2.22–2.15 (1H, m), 0.98 (3H, d, *J* = 6.9 Hz); ^{13}C NMR (126 MHz, $CDCl_3$) δ 156.0, 151.6, 149.1, 107.4, 63.5, 48.8, 37.6, 30.0, 28.3, 14.3; HRMS (APCI): Found $M+H^+$ 253.1295, $C_{11}H_{17}O_3N_4$ requires 253.1301.

tert-Butyl 3-(Hydroxymethyl)-8-azabicyclo[3.2.1]octane-8-carboxylate (27)

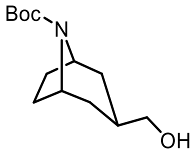

Following **GP6**, **S12** (34 mg, 0.1 mmol) gave **27** (17 mg, 70%) as an oil. ^{1}H NMR (500 MHz, $CDCl_3$) δ 4.36–4.14 (2H, m), 3.43 (2H, d, *J* = 6.2 Hz) 2.15–1.99 (2H, m), 1.95 (2H, dt, *J* = 7.5, 2.8 Hz), 1.75–1.61 (3H, m), 1.61–1.52 (2H, m), 1.45 (9H, s); ^{13}C NMR (126 MHz, $CDCl_3$) δ 153.6, 79.2, 77.2, 68.2, 31.5, 28.7. Note: some peaks aren't visible due to the conformation flip of the compound. Data in accordance with literature.[29]

((8*S*,9*S*,10*R*,13*R*,14*S*,17*R*)-10,13-Dimethyl-17-((*R*)-6-methylheptan-2-yl) 2,3,4,7,8,9,10,11,12,13,14,15,16,17-tetradecahydro-1*H*-cyclopenta[a]phenanthren-3-yl)methanol (29)

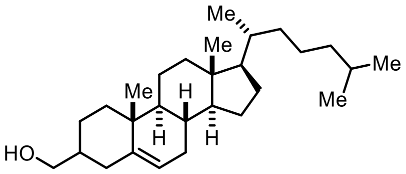

Following **GP6**, **S13** (50 mg, 0.1 mmol) gave **29** (16 mg, 40%) as an oil as a mixture of diastereomers. dr 2.3:1 ^{1}H NMR (500 MHz, $CDCl_3$, diastereomers) δ 5.32–5.27 (1H, m), 3.57–3.46 (2H, m), 2.53–2.44 (1H, m), 2.10–0.85 (41H, m), 0.67 (3H, d, *J* = 2.4 Hz); ^{13}C NMR (126 MHz, $CDCl_3$, diastereomers) δ 142.0, 140.2, 121.3, 119.9, 68.4, 63.8, 56.7, 56.1, 50.4, 50.3, 42.2, 42.0, 39.8, 39.7, 39.4, 38.9, 37.4, 37.3, 36.7, 36.1, 35.7, 34.3, 33.7, 31.8, 28.2, 27.9, 25.3, 24.2, 23.8, 23.1, 22.8, 22.5, 20.9, 20.7, 19.4, 19.3, 18.6, 11.8. Data in accordance with literature.[4]

6 Computational Studies

6.1 Computational Methods

Density functional theory (DFT)[30] calculations were performed using Gaussian 09 (revision E.01)[31] and the Gaussview[3] was used to generate input geometries and visualize output structures. Geometry optimizations and frequency calculations for the calculation of reaction energies were performed using the B3LYP functional[32] and an atom-pairwise dispersion correction (D3)[33] with a flexible triplet zeta basis set (def2-TZVP)[34] using acetonitrile to model solvation effect by applying the most commonly used integral equation formalism (IEF) version of polarized continuum model (PCM).[35] The bond distance [*d*(C–C)] between the carbon radical centre and carbon atom of CO or CH_2O was calculated from the transition states structures and the amount of charge transfer (δ^{TS}) from the carbon radical to the carbon atom of CO or CH_2O in the transition states was evaluated from the Mulliken charges. For calculation of electronic properties of phosphoranyl radical, global and local electrophilicity index, B3LYP functional.[32b, 32d, 36] was used and the geometry of studied radical was optimized at the UB3LYP/6-311+G(d,p) level of theory, followed by frequency calculation at the same level.[37] The computed Hirshfeld charges on the radicals were also calculated at the same level of theory.[38] All stationary points were characterized as minima based on normal vibrational mode analysis. Thermal corrections were computed from unscaled frequencies, assuming a standard state of 298.15 K and 1 atm.

6.2 Electronic Properties of Phosphoranyl Radical

DFT Method: UB3LYP/6-311+G(d,p)

Phosphoranyl Radical	Ionization Potential (IP, eV)	Electron affinity (EA, eV)	Electronegativity (χ, eV)	Electronic Chemical Potential (μ, eV)	Chemical Hardness (η, eV)	Chemical Softness (S, meV)	Global Electrophilicity Index (ω, eV)	Local Electrophilicity Index (ω□$_{rc}$, eV)	Hirshfeld Charge
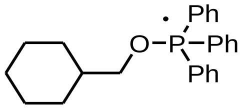	4.20	0.27	2.23	-2.23	3.93	254.31	0.64	0.07	0.3621

Computed Energies [values are in Hartree]

Phosphoranyl Radical	Total Electronic Energy	Sum of Electronic and Zero-point Energies	Sum of Electronic and Thermal Enthalpies	Gibbs Free Energy
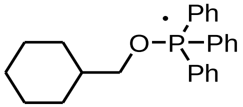	-1386.3564642	-1385.892647	-1385.866697	-1385.950731

Optimized Structure and Cartesian Coordinates

No.	Phosphoranyl Radical	Optimized Structure
1	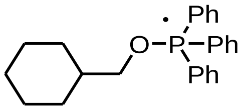	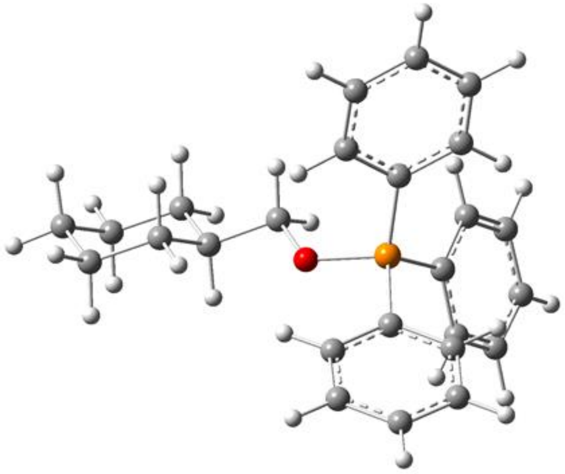

Cartesian Coordinates

C	-3.08059200	0.38203900	-0.26609100
C	-4.10383100	1.38502100	0.29859800
C	-5.48772600	1.21116200	-0.34397300
C	-5.99936200	-0.22993900	-0.20981900
C	-4.98197800	-1.23639600	-0.76428600
C	-3.60080900	-1.05823300	-0.11791600
H	-6.19923000	1.91039400	0.10719900
H	-4.18927600	1.23929400	1.38427500
H	-3.74697900	2.40981000	0.14804600
H	-2.95101300	0.59255000	-1.33644300
H	-6.18431500	-0.45021600	0.84987500
H	-6.96071300	-0.33900400	-0.72245200
H	-5.33806300	-2.26030300	-0.61039700
H	-4.89213200	-1.09862000	-1.84971600
H	-3.66763700	-1.30127300	0.95198200
H	-2.88323400	-1.75796600	-0.55471200
H	-5.42477400	1.47449200	-1.40766800
C	-1.72244200	0.58522500	0.39640500
H	-1.41801300	1.63433600	0.31577900
H	-1.76787600	0.32214600	1.46039600
O	-0.74270000	-0.24746200	-0.26118500
P	0.89215200	0.02607200	0.00024500
C	1.51219600	-1.11469300	-1.27678900
C	0.70739200	-2.11644500	-1.84189800
C	2.88217800	-1.08664000	-1.58556400
C	1.26536200	-3.06028800	-2.70314800

H	-0.34961600	-2.14125800	-1.61686100
C	3.42733100	-2.01761500	-2.46156300
H	3.51847600	-0.33091300	-1.13878700
C	2.62071500	-3.01286700	-3.01973200
H	0.63392000	-3.82950200	-3.13421300
H	4.48349700	-1.97380700	-2.70249600
H	3.04932900	-3.74636900	-3.69328800
C	1.48946000	1.65358300	-0.28274900
C	1.52893600	2.64267200	0.74781600
C	1.75155000	2.08948200	-1.61833300
C	1.89830300	3.94475700	0.46518300
H	1.26424700	2.37796700	1.76380200
C	2.11791700	3.39890800	-1.87647600
H	1.67227700	1.38817100	-2.43980800
C	2.20982000	4.34282200	-0.84454800
H	1.93433100	4.66900000	1.27224600
H	2.32948800	3.69485100	-2.89868500
H	2.49183500	5.36679500	-1.05689300
C	1.17438500	-0.52737700	1.68215500
C	0.39437100	-1.59402000	2.18665100
C	2.26438300	-0.07152500	2.45699600
C	0.68197700	-2.15530500	3.42666800
H	-0.43464500	-1.97615400	1.60386800
C	2.52705500	-0.62563800	3.70042200
H	2.91027900	0.70867300	2.07465300
C	1.73858900	-1.67234800	4.19733400
H	0.07088500	-2.97217700	3.79533300
H	3.36168400	-0.25190100	4.28333900
H	1.95551300	-2.10842200	5.16537800

6.3 Reaction Energies

DFT Method: B3LYP-D3/def2-TZVP [solvent: CH_3CN, values are in Kcal mol^{-1}]

**Addition Reactions**	**$\Delta G^{\neq}$**	**ΔG**	***d*(C–C) (Å)**	**δ^{TS}**
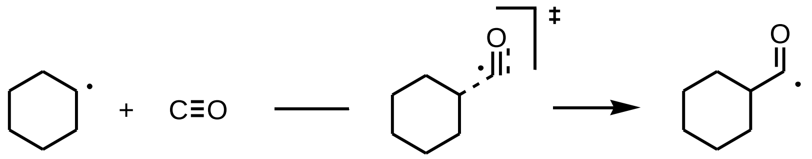	10.2	-0.7	2.27196	0.109536
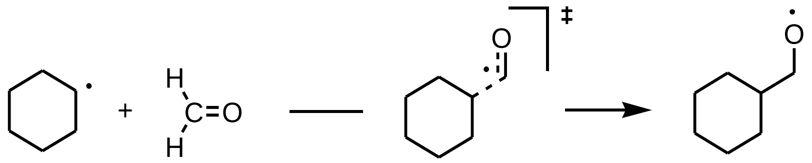	9.9	4.8	2.19618	0.128423
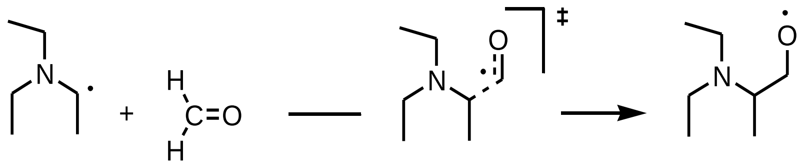	3.9	4.6	2.00949	0.158047

Computed Energies [values are in Hartree]

**Species**	**Total Electronic Energy**	**Sum of Electronic and Zero-point Energies**	**Sum of Electronic and Thermal Enthalpies**	**Gibbs Free Energy**
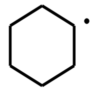	-235.3134492	-235.158424	-235.151457	-235.188113
C≡O	-113.3631968	-113.358162	-113.354857	-113.377278
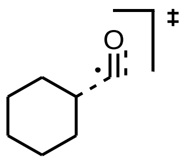	-348.6766246	-348.513916	-348.504598	-348.549078
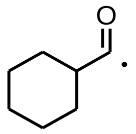	-348.7006279	-348.533770	-348.525235	-348.566516
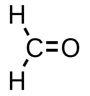	-114.5596746	-114.533057	-114.529246	-114.554060
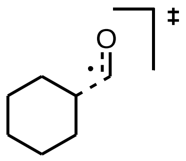	-349.8782668	-349.692471	-349.683177	-349.726337

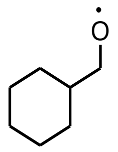	-349.8905406	-349.702052	-349.693384	-349.734564
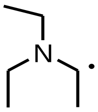	-291.8948752	-291.703445	-291.692932	-291.738154
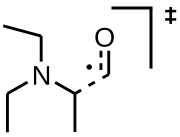	-406.4698197	-406.247269	-406.234509	-406.286078
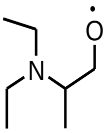	-406.4701611	-406.246298	-406.233375	-406.284919

Optimized Structures and Cartesian Coordinates

No.	Species	Optimized Structure
1	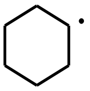	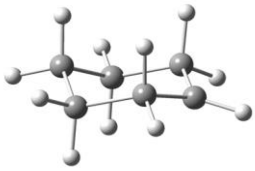

Cartesian Coordinates

C	1.26397300	-0.71045000	-0.24239500
C	1.28286100	0.77605000	0.15890500
C	0.00000100	1.45997600	-0.17061900
C	-1.28286000	0.77605100	0.15890400
C	-1.26397400	-0.71045000	-0.24239400
C	0.00000000	-1.40798200	0.26742700
H	1.45609800	0.82755100	1.24886100
H	2.13003100	1.28862500	-0.30343300
H	1.29813000	-0.78597100	-1.33447200
H	2.15786900	-1.20990500	0.14011700
H	-1.45609900	0.82755400	1.24886000
H	-2.13002900	1.28862600	-0.30343600
H	-2.15786900	-1.20990400	0.14012000
H	-1.29813200	-0.78597100	-1.33447100
H	0.00000000	-1.39975200	1.36403100
H	-0.00000100	-2.45748300	-0.03895400
H	0.00000100	2.51746400	-0.40619000

No.	Species	Optimized Structure
2	C≡O	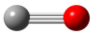

Cartesian Coordinates

C	0.00000000	0.00000000	-0.64266700
O	0.00000000	0.00000000	0.48200000

3

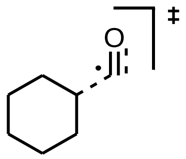

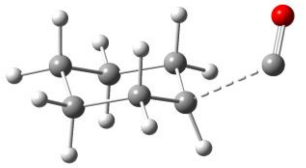

Cartesian Coordinates

C	-0.45667500	-0.17569500	0.72470200
C	0.15812000	-1.33743400	0.01060600
C	-0.10143300	1.17507200	0.19387300
H	-0.57760000	-0.26908900	1.79899600
C	1.69735400	-1.17854800	-0.00203400
H	-0.18859700	-1.36052100	-1.02927700
H	-0.12593700	-2.28473900	0.47191000
C	1.43781500	1.33571500	0.17580100
H	-0.46903600	1.27579500	-0.83395500
H	-0.56065200	1.97032600	0.78384100
C	2.10336100	0.18095100	-0.57794000
H	2.14316900	-1.99158400	-0.58102200
H	2.07546700	-1.26945000	1.02144800
H	1.69792000	2.29464100	-0.28020300
H	1.80788100	1.36017300	1.20574700
H	3.19042500	0.29133500	-0.54700400
H	1.81156000	0.22532900	-1.63335500
C	-2.66053000	-0.41826000	0.22870800
O	-2.98408400	0.28337400	-0.61242900

4

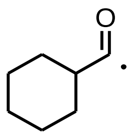

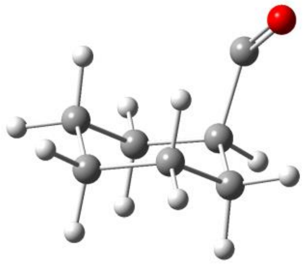

Cartesian Coordinates

C	-1.86843800	0.47132800	-0.39383600
C	-0.71546000	1.44795300	-0.14151000
C	0.18985600	0.96924400	0.99669500
C	0.69287400	-0.46748300	0.76821800
C	-0.46318100	-1.44093600	0.48518700

C	-1.34967700	-0.94490900	-0.66022700
H	-0.12257300	1.55106100	-1.05589200
H	-1.10327500	2.44214500	0.09272900
H	-2.52482900	0.45290500	0.48381500
H	-2.47580400	0.81497800	-1.23472200
H	1.23739000	-0.81922000	1.65297800
H	-0.06725200	-2.43497800	0.26807300
H	-1.06052300	-1.52346700	1.39752000
H	-0.77311200	-0.95024300	-1.59289800
H	-2.18358900	-1.63557800	-0.80499800
H	1.03872600	1.64328500	1.12401300
H	-0.37157800	0.97451600	1.93620400
C	1.75174000	-0.54647800	-0.32067500
O	2.37251700	0.31903500	-0.83374100
5	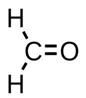	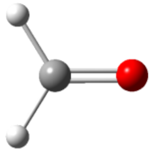	
Cartesian Coordinates			
C	0.00000000	0.00000000	-0.52939300
O	0.00000000	0.00000000	0.67536600
H	0.00000000	-0.93778400	-1.11328400
H	0.00000000	0.93778400	-1.11328400
6	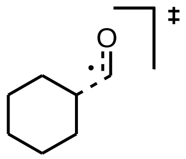	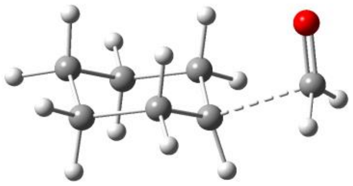	
Cartesian Coordinates			
C	0.41183800	0.00086800	0.72013000
C	-0.07769000	1.26541300	0.09524400
C	-0.07642800	-1.26459800	0.09616500
H	0.52570300	0.00131200	1.80108500
C	-1.62641800	1.26526100	0.09356200
H	0.27149900	1.31646600	-0.94070500
H	0.30246300	2.14349900	0.61968300
C	-1.62514300	-1.26597300	0.09469000
H	0.27274200	-1.31596600	-0.93976600
H	0.30470200	-2.14194300	0.62114200

C	-2.17412000	-0.00092600	-0.56917300
H	-1.98420900	2.15961600	-0.42232600
H	-1.98792100	1.32422200	1.12502700
H	-1.98216200	-2.16117200	-0.42026600
H	-1.98645700	-1.32425300	1.12626200
H	-3.26633500	-0.00145600	-0.52735300
H	-1.89756600	-0.00125500	-1.62920200
C	2.57061700	0.00112300	0.31656900
O	2.71065600	-0.00136000	-0.91196800
H	2.66266400	0.93353200	0.89755800
H	2.66369400	-0.92872800	0.90147400

7	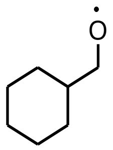	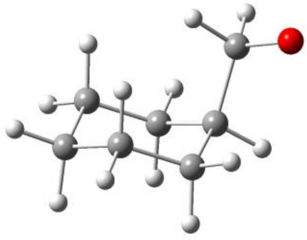

Cartesian Coordinates

C	-2.05368500	0.37682300	-0.32356100
C	-0.93520000	1.42155800	-0.27926200
C	0.09458700	1.09825000	0.80954600
C	0.66047600	-0.32450200	0.69528900
C	-0.46707500	-1.36463100	0.59671600
C	-1.48670600	-1.03456300	-0.49947400
H	-0.44415700	1.46439300	-1.25780900
H	-1.35136200	2.41702000	-0.10498300
H	-2.62196700	0.41957100	0.61294500
H	-2.75585100	0.60676600	-1.12908900
H	1.24532800	-0.53781000	1.59575300
H	-0.04623100	-2.36196200	0.43764700
H	-0.98466000	-1.39737700	1.56088500
H	-1.01560100	-1.11407400	-1.48565100
H	-2.29218100	-1.77320000	-0.48666200
H	0.91517200	1.81895200	0.78519100
H	-0.38452100	1.19899300	1.78887300
C	1.64726000	-0.46964900	-0.47802800
H	1.93720000	-1.52209700	-0.64096300
H	1.14877200	-0.17987900	-1.42912100
O	2.73651400	0.34262400	-0.42179600

8

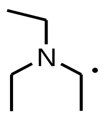

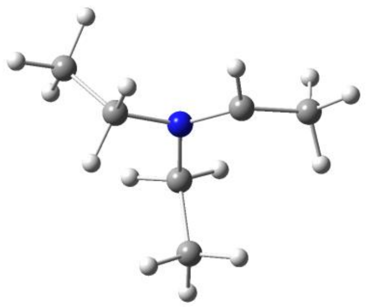

Cartesian Coordinates

C	0.84583400	-1.11049900	-0.45482600
N	-0.12060300	-0.23894100	0.03355900
C	-1.43388000	-0.23598700	-0.60541800
H	-1.45366300	-1.04061000	-1.34096200
H	-1.58164500	0.69667800	-1.16323900
C	-2.57442500	-0.42760400	0.39091400
H	-2.58834800	0.36503600	1.14098600
H	-3.53614300	-0.41251300	-0.12720000
H	-2.47420000	-1.38397900	0.90735100
C	0.29344000	1.02296300	0.64013500
H	-0.57571100	1.45508500	1.13831800
H	1.02305400	0.81402400	1.42398100
C	2.15508600	-1.25209000	0.24996200
H	2.04467300	-1.56672300	1.30125400
H	2.76074200	-2.00720500	-0.25257600
H	2.73679400	-0.32499400	0.26427300
C	0.87682500	2.03484400	-0.35171300
H	0.15300200	2.29096500	-1.12747600
H	1.15562600	2.95445200	0.16691900
H	1.76542400	1.62977600	-0.83796400
H	0.43733600	-1.98716700	-0.94290600

9

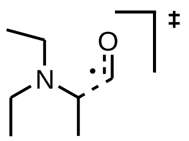

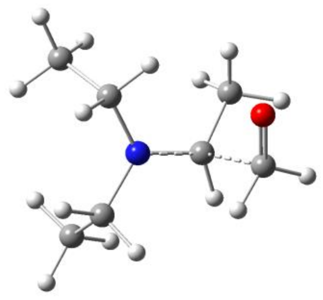

Cartesian Coordinates

C	-0.57928100	-0.47232000	0.71143600
N	0.42756700	-0.09952800	-0.13426900

C	1.33410000	-1.12711800	-0.64470100
H	0.75406500	-2.02328000	-0.88443100
C	0.45580400	1.22635000	-0.74499600
H	0.67929400	1.10284500	-1.80706300
H	-0.54654700	1.64697200	-0.68695700
C	-1.14008300	0.52888800	1.68559700
H	-1.55830700	1.39841800	1.18115300
H	-1.93505100	0.06617200	2.26909400
H	-0.36160000	0.86944700	2.37411000
C	1.47824800	2.16419800	-0.10469900
H	2.48865300	1.76268200	-0.19577200
H	1.45245500	3.13674700	-0.60007100
H	1.26159200	2.31030500	0.95415700
H	-0.41742800	-1.46322100	1.12702800
C	2.44861500	-1.47680700	0.34019000
H	3.08476800	-2.25957500	-0.07729900
H	3.06775500	-0.60463600	0.55316500
H	2.03476700	-1.84124100	1.28186200
H	1.76170800	-0.76632600	-1.57994600
C	-2.18264800	-0.93250900	-0.40904800
H	-1.64382100	-1.65286600	-1.05074700
H	-2.71049400	-1.41916800	0.43321200
O	-2.66166400	0.12116600	-0.91928700

10	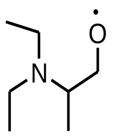	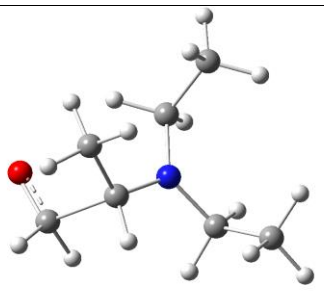

Cartesian Coordinates

C	-0.61501100	-0.52697200	0.64565600
N	0.43262900	-0.09099800	-0.15221900
C	1.38961400	-1.07790200	-0.64243800
H	0.85641100	-2.00354500	-0.88128100
C	0.38429500	1.22448600	-0.77629400
H	0.74260500	1.12555500	-1.80295100
H	-0.66330300	1.52559800	-0.83561000
C	-1.15752300	0.44746200	1.67235800
H	-1.52315800	1.36221200	1.21086200

H	-1.98584700	-0.01179000	2.21124600
H	-0.37703200	0.70503800	2.39090600
C	1.21402800	2.28157200	-0.04506900
H	2.26620100	1.99480400	-0.01139600
H	1.13653100	3.23775900	-0.56635400
H	0.86338000	2.41813800	0.97788400
H	-0.36751600	-1.48923900	1.09260400
C	2.50490600	-1.36704600	0.36228100
H	3.18274900	-2.12341100	-0.03814100
H	3.07884100	-0.46402900	0.57381500
H	2.09443500	-1.74068900	1.30187600
H	1.81576700	-0.70738400	-1.57468800
C	-1.99380700	-1.00431900	-0.37718000
H	-1.49488500	-1.72522600	-1.05218500
O	-2.55500100	0.02572600	-0.92140000
H	-2.57258600	-1.53629600	0.40426900

7 NMR Spectra

S1 - ^{1}H NMR (500 MHz, $CDCl_3$)

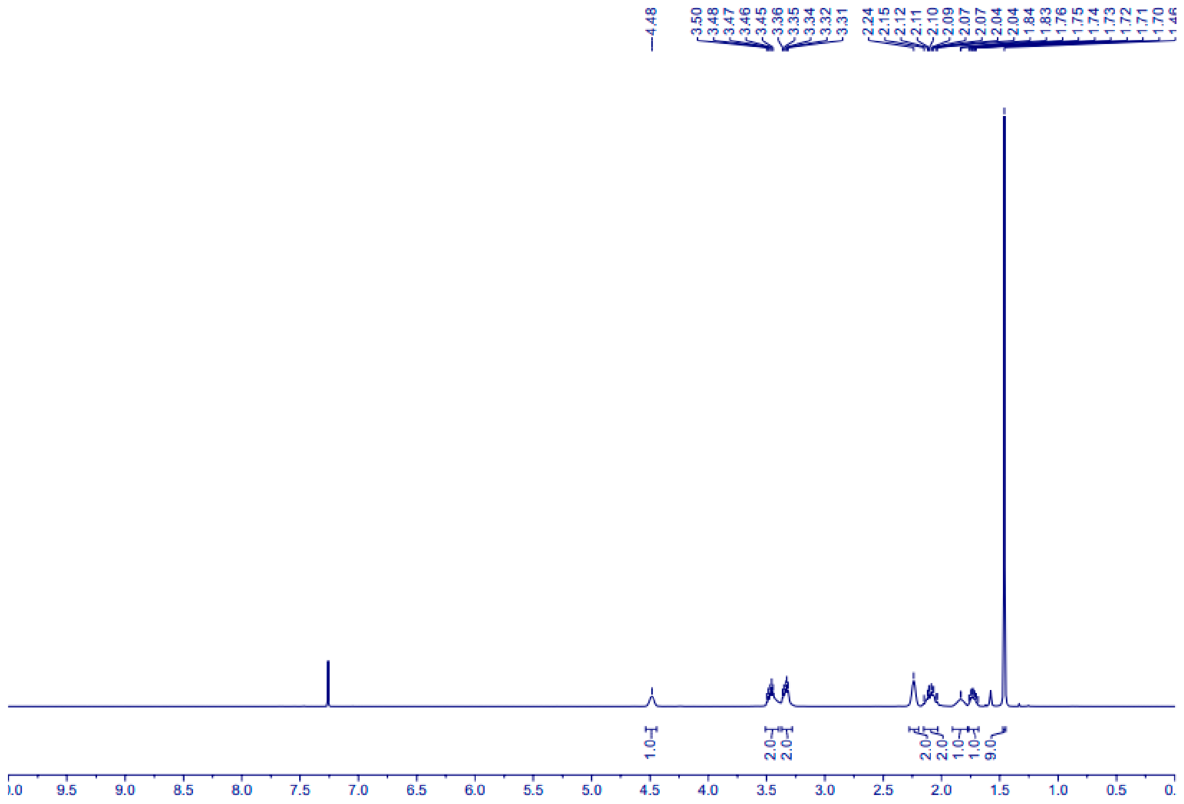

S1 - ^{13}C NMR (101 MHz, $CDCl_3$)

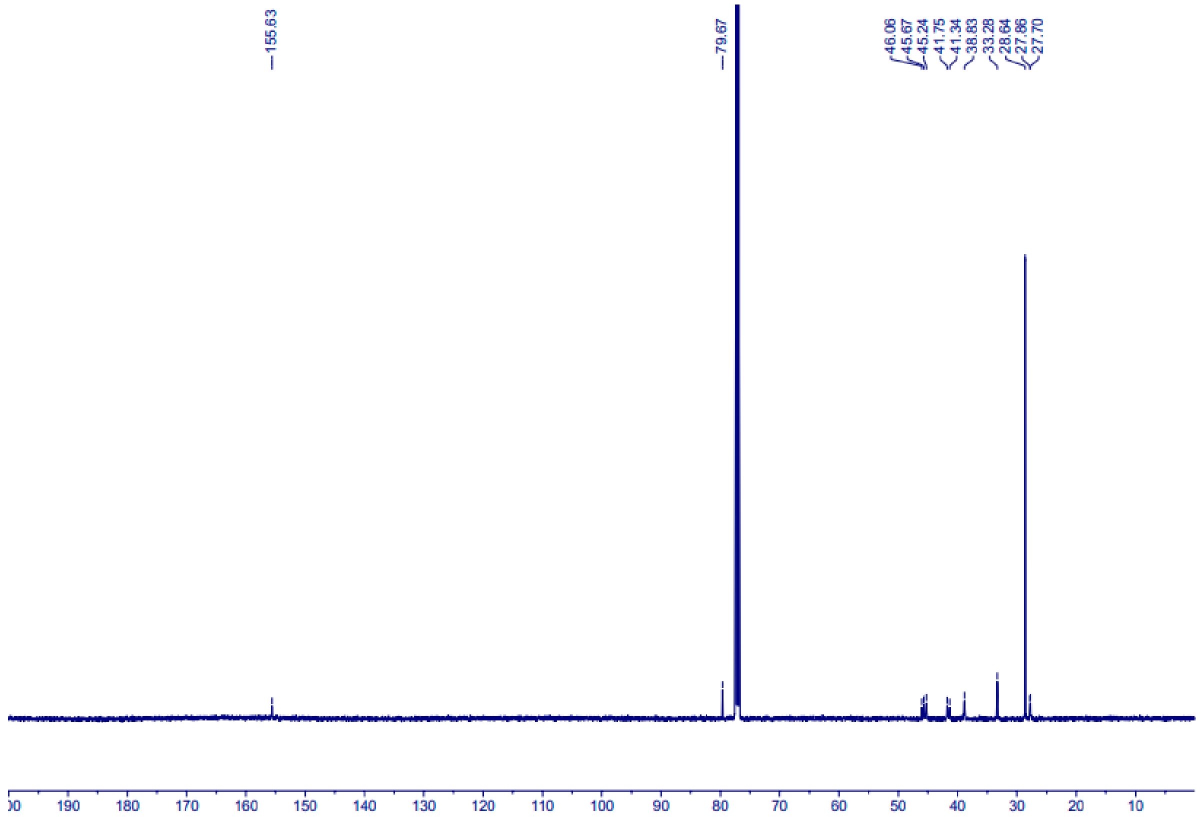

S6 - ^{1}H NMR (400 MHz, $CDCl_3$)

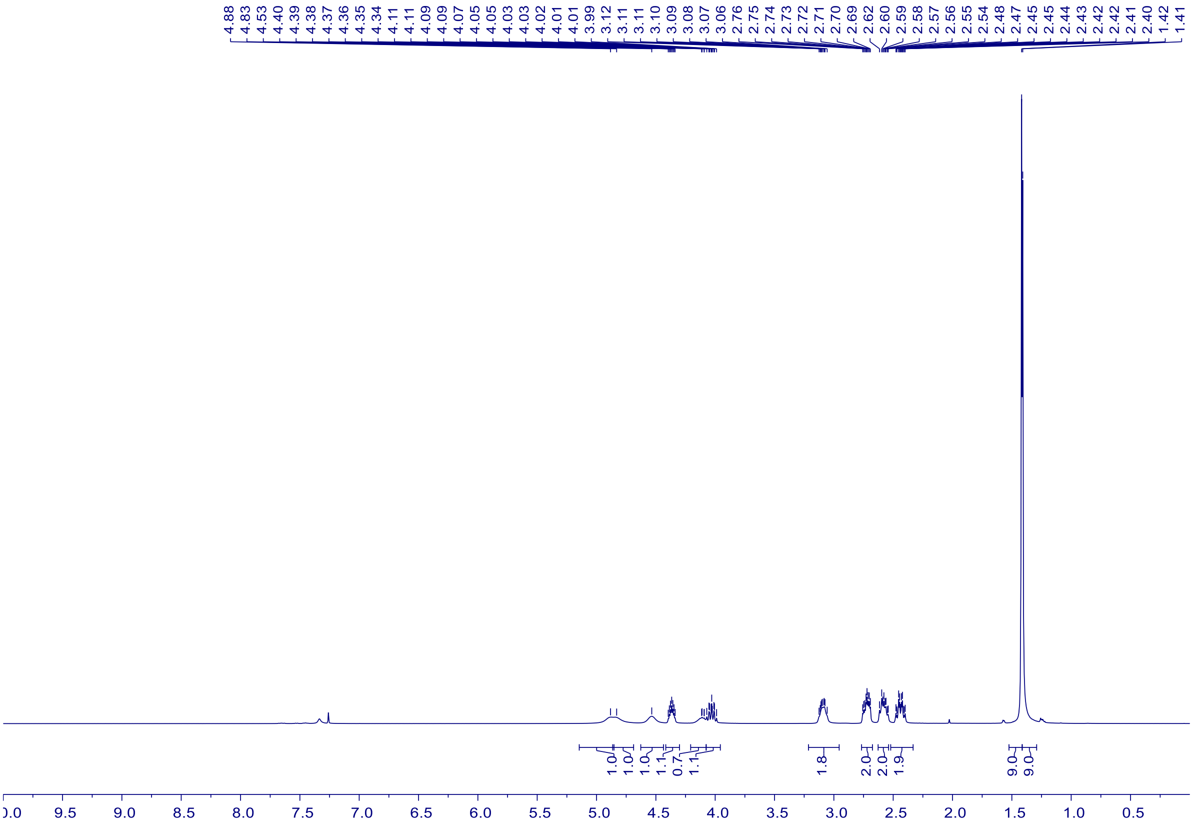

S6 - ^{13}C NMR (101 MHz, $CDCl_3$)

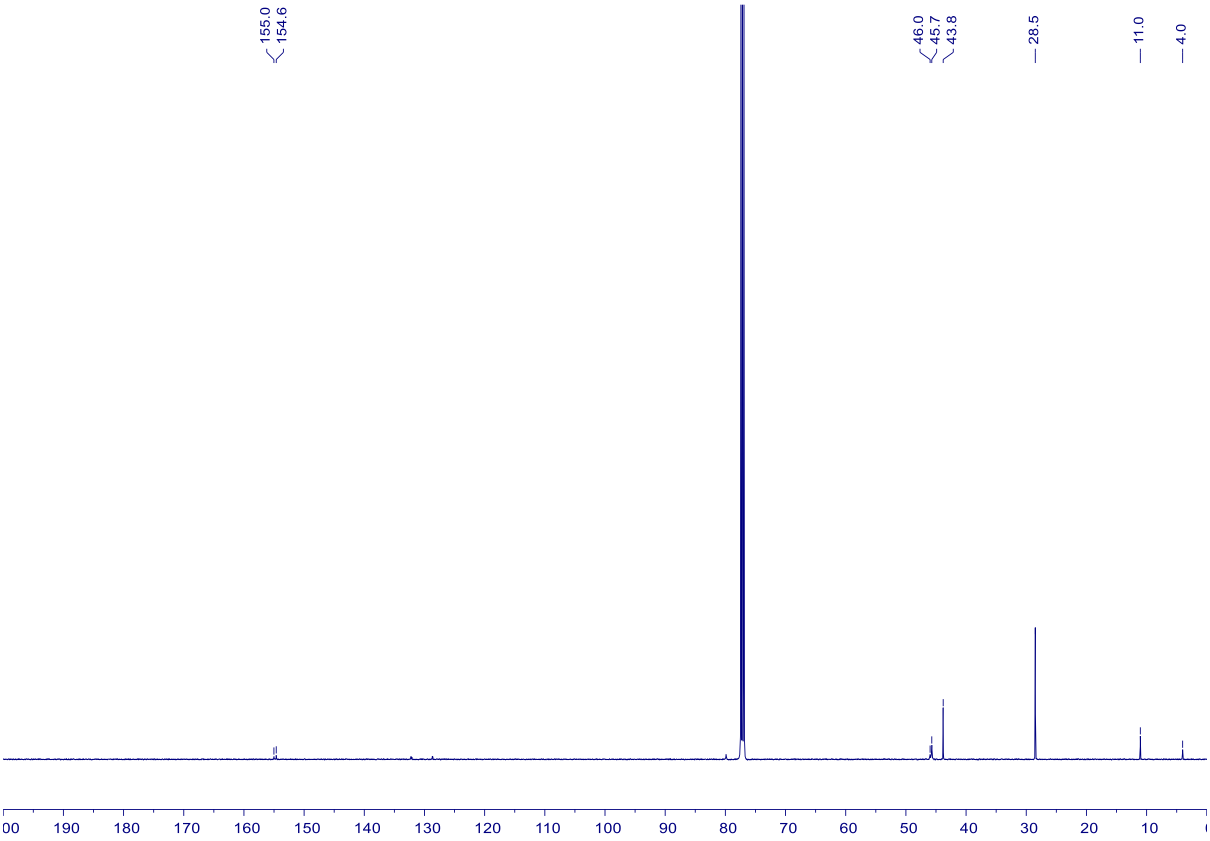

S9 – ^{1}H NMR (400 MHz, CDCl$_3$)

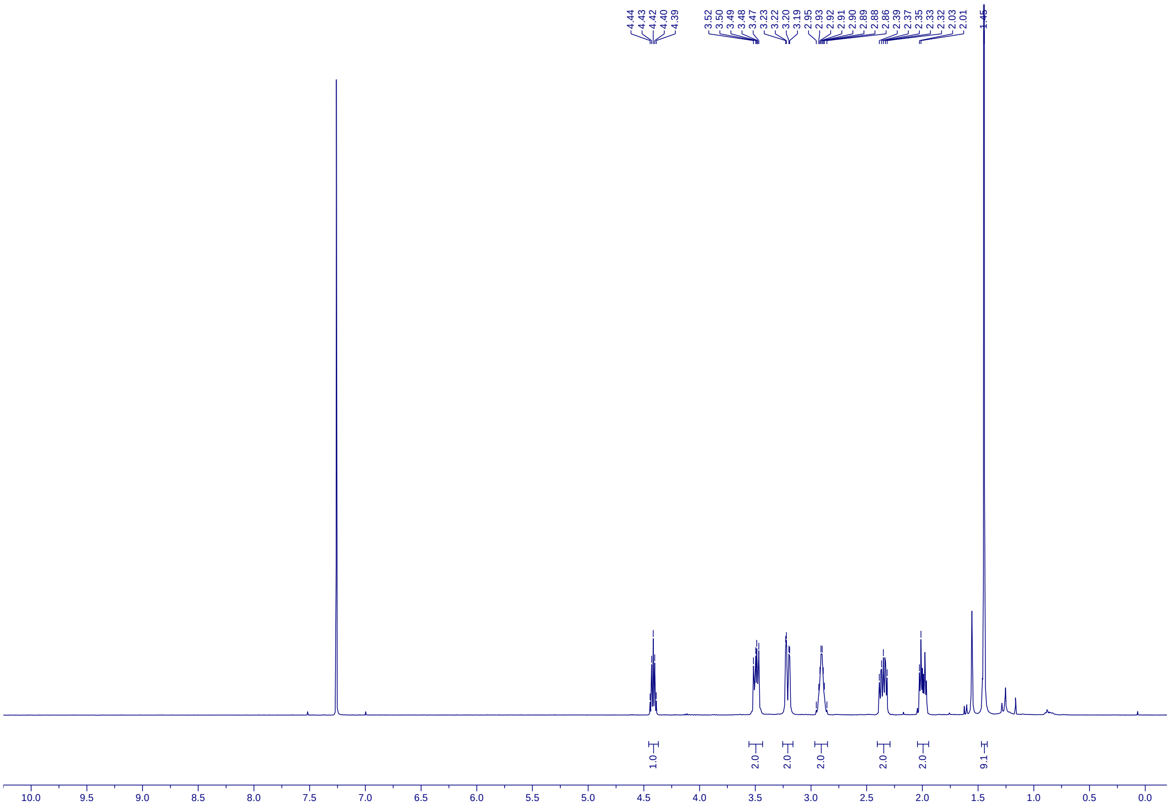

S9 – ^{13}C NMR (101 MHz, CDCl$_3$)

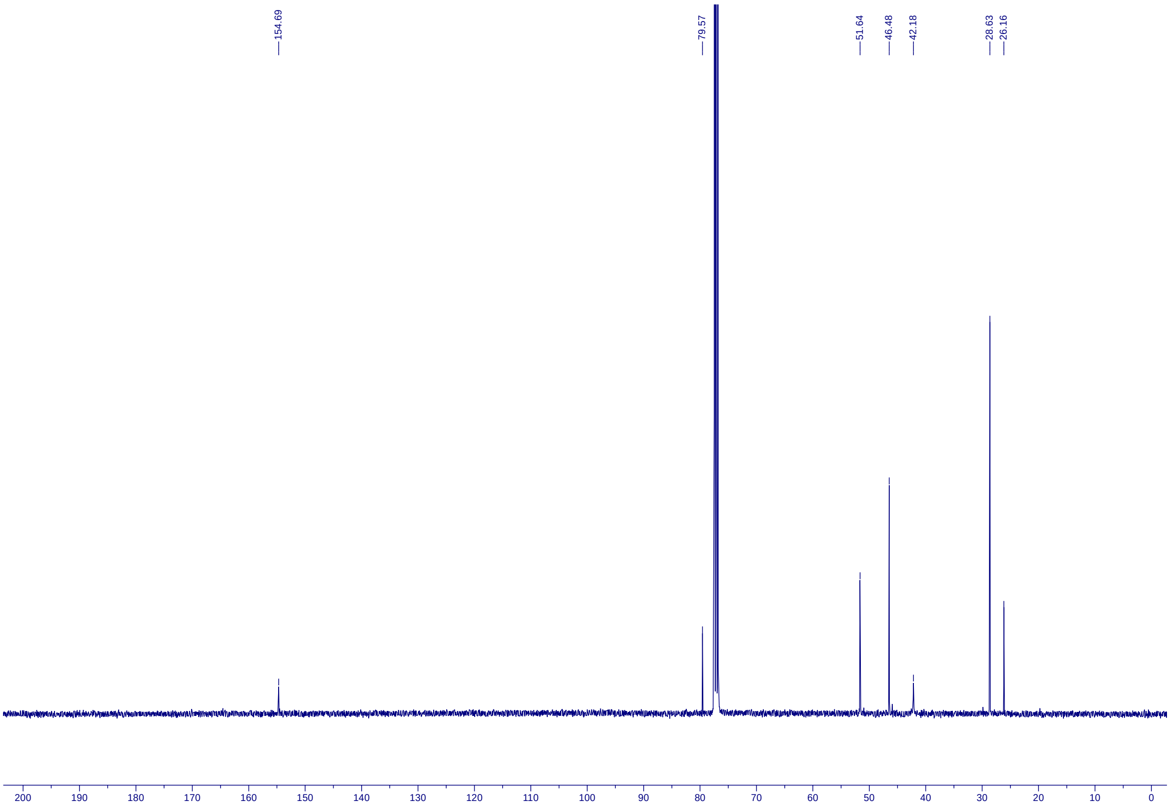

S10 – ^{1}H NMR (400 MHz, $CDCl_3$)

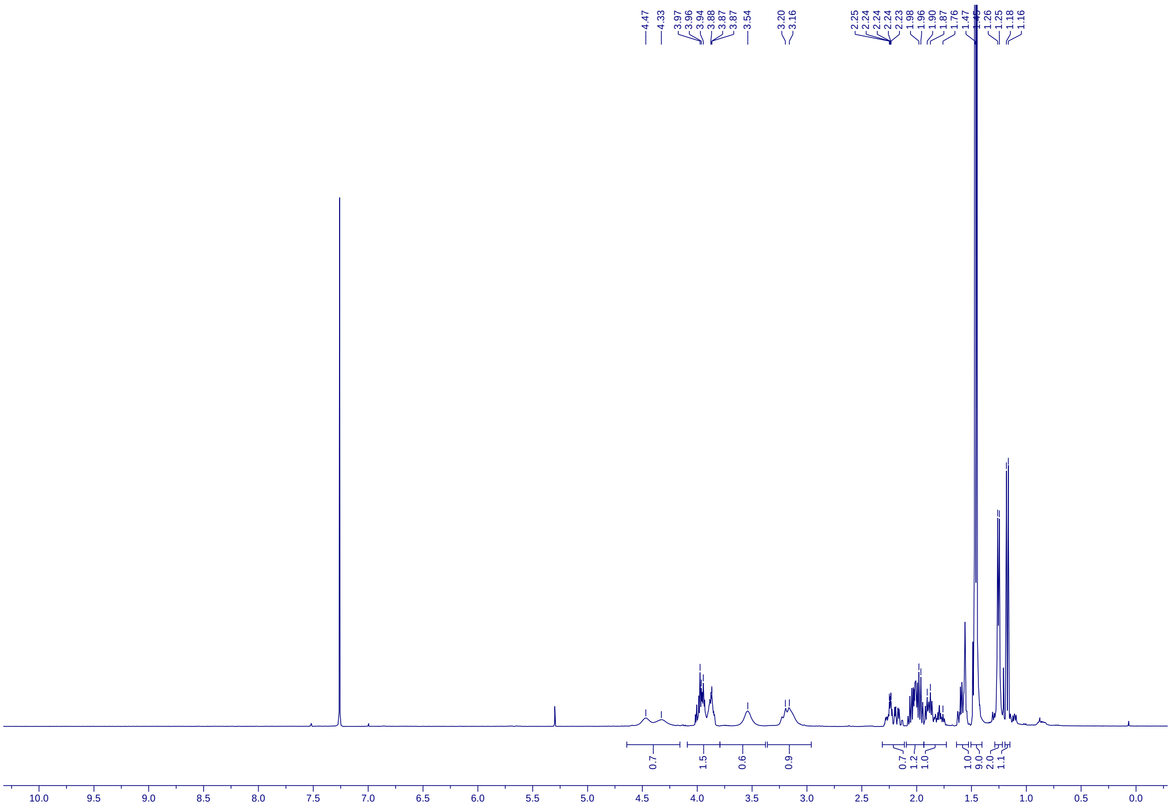

S10 – ^{13}C NMR (101 MHz, $CDCl_3$)

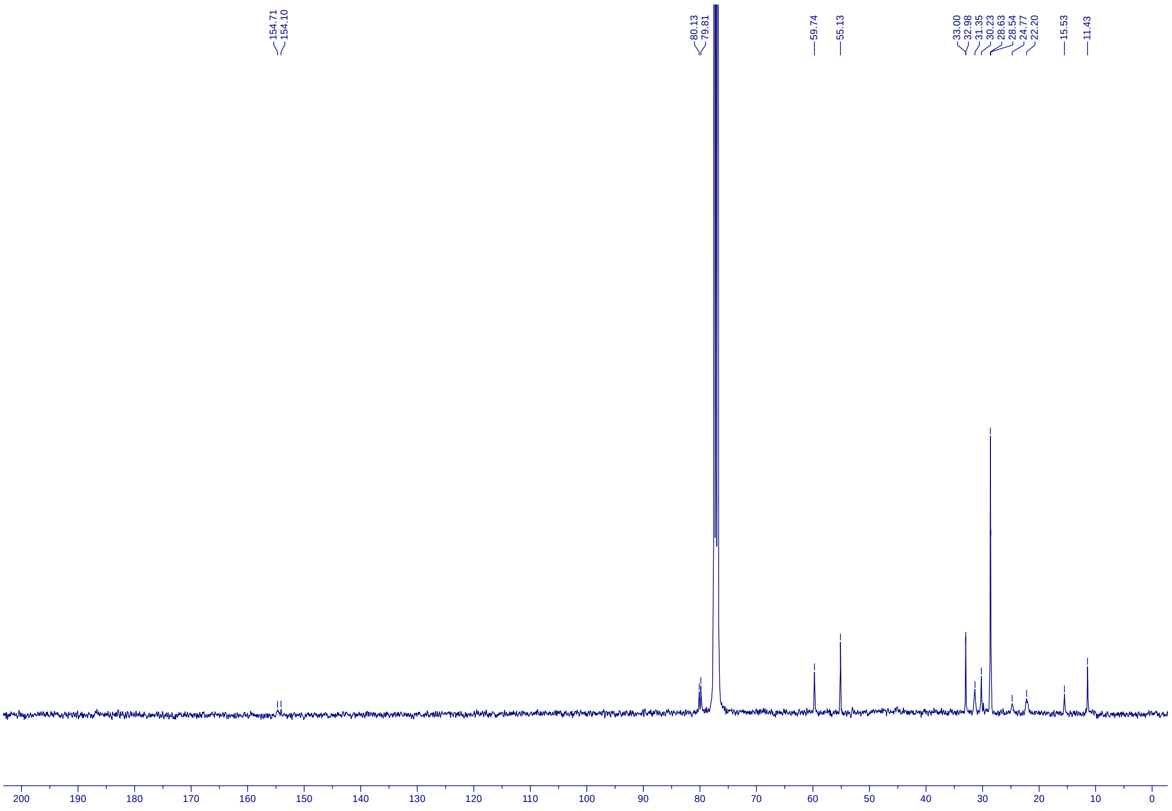

S11 - ^{1}H NMR (500 MHz, $CDCl_3$)

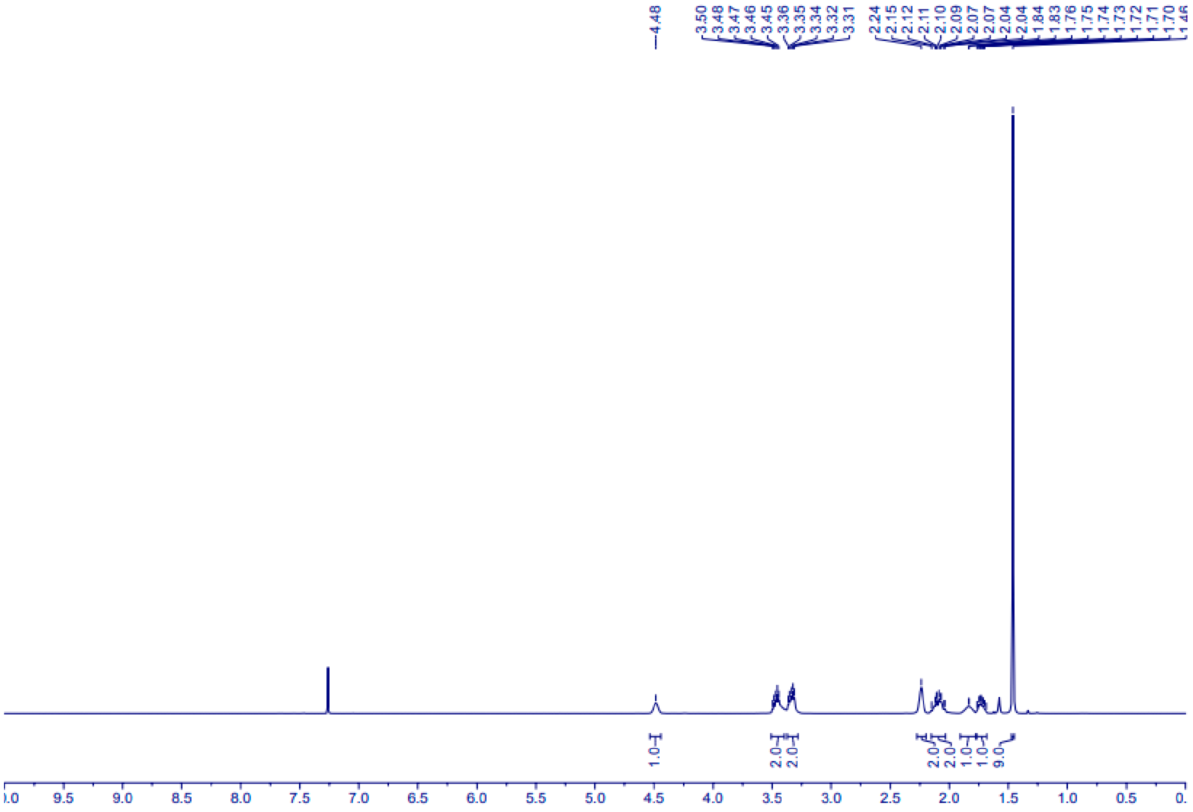

S11 - ^{13}C NMR (101 MHz, $CDCl_3$)

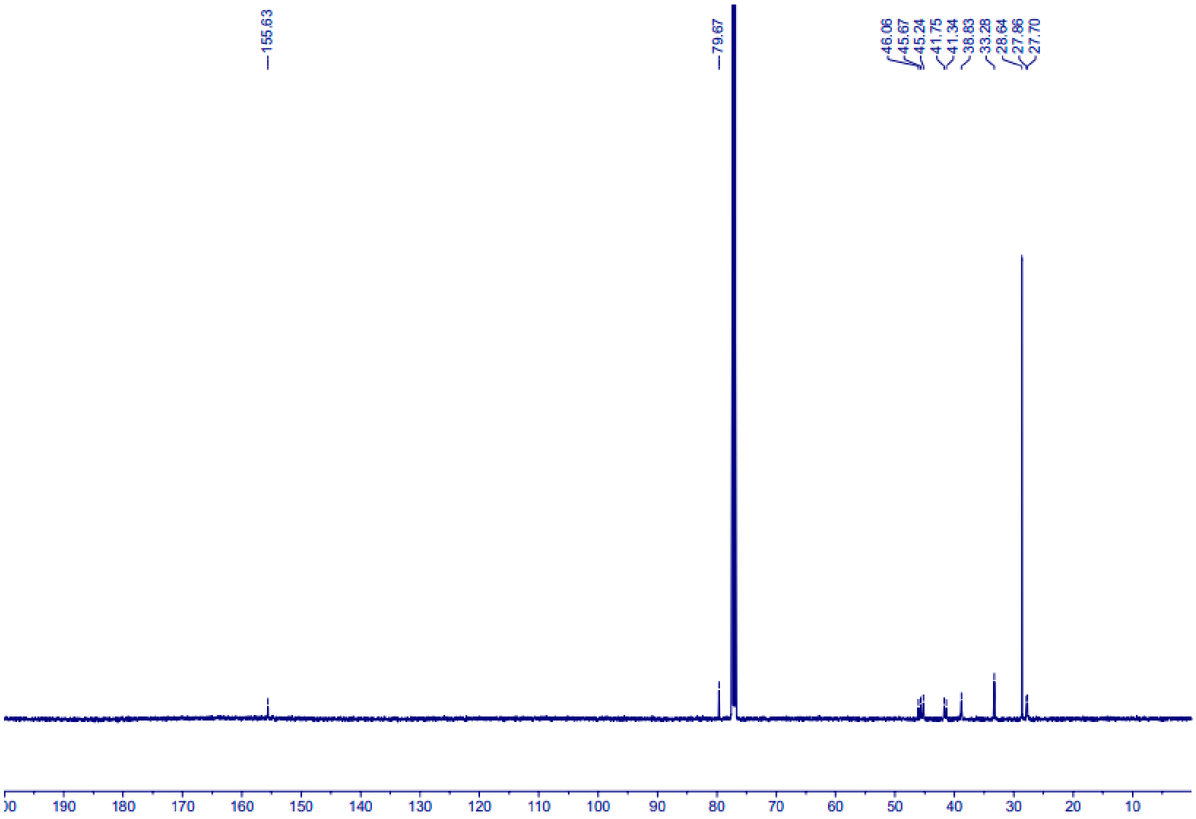

S12 - ^{1}H NMR (400 MHz, $CDCl_3$)

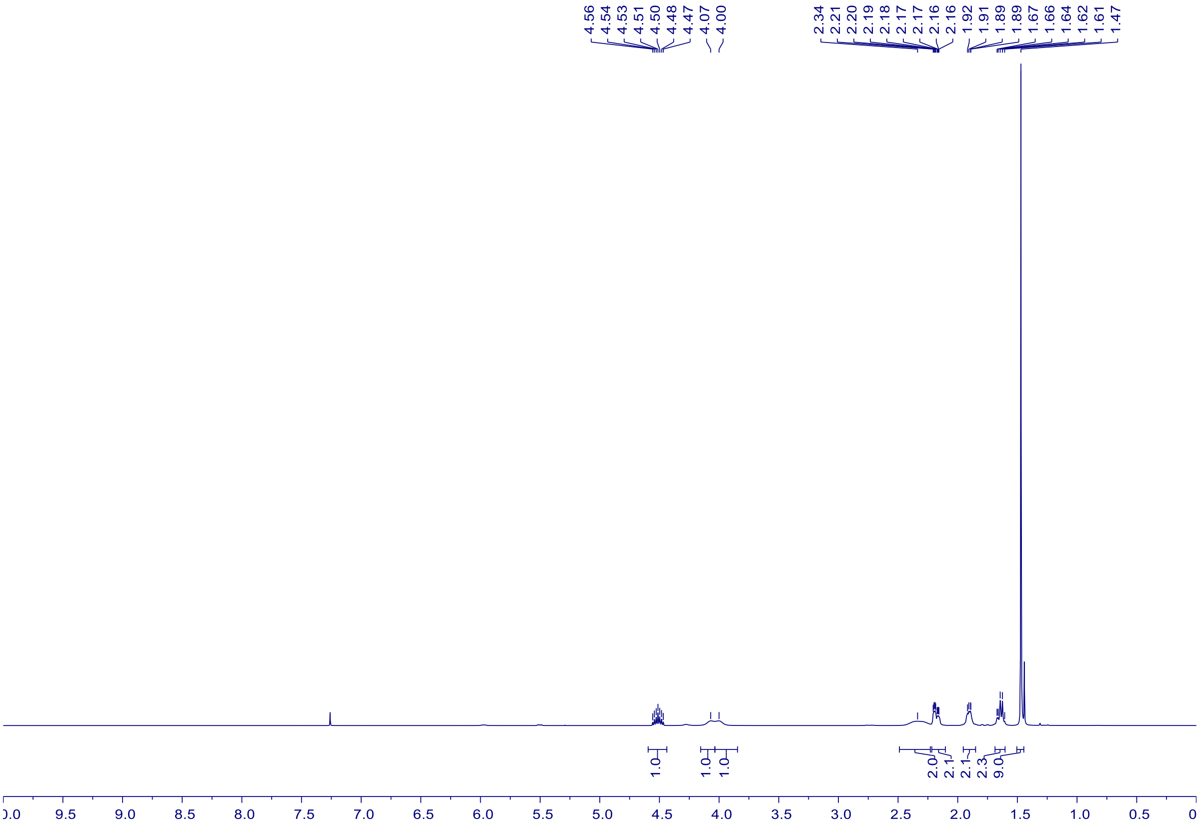

S12 - ^{13}C NMR (101 MHz, $CDCl_3$)

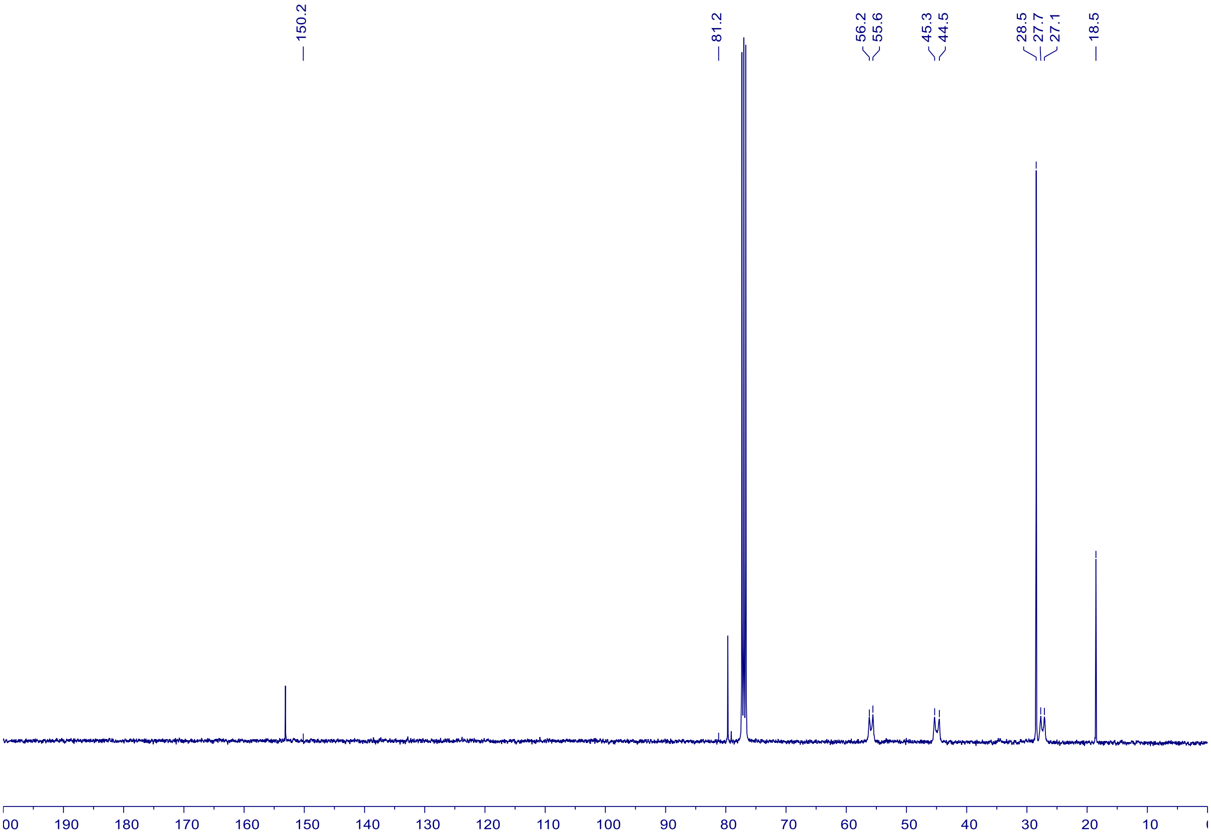

4 - ^{1}H NMR (400 MHz, $CDCl_3$)

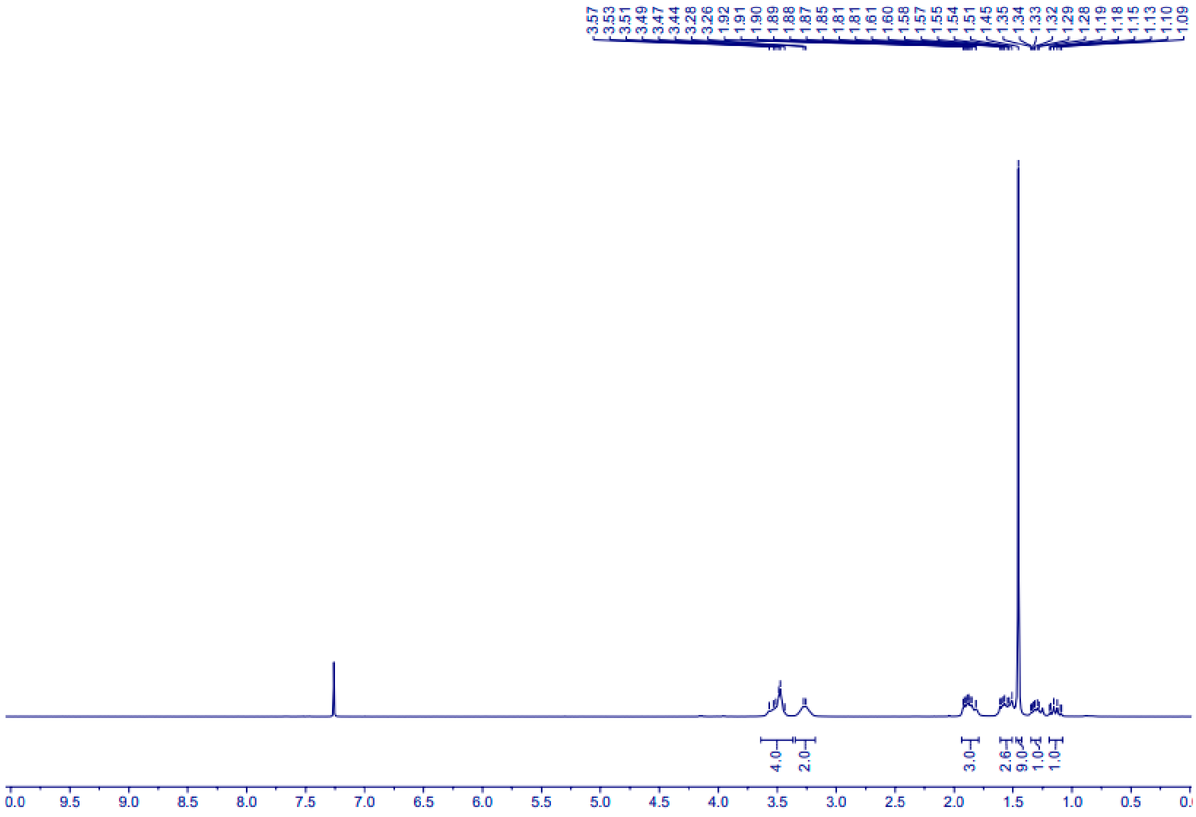

4 - ^{13}C NMR (101 MHz, $CDCl_3$)

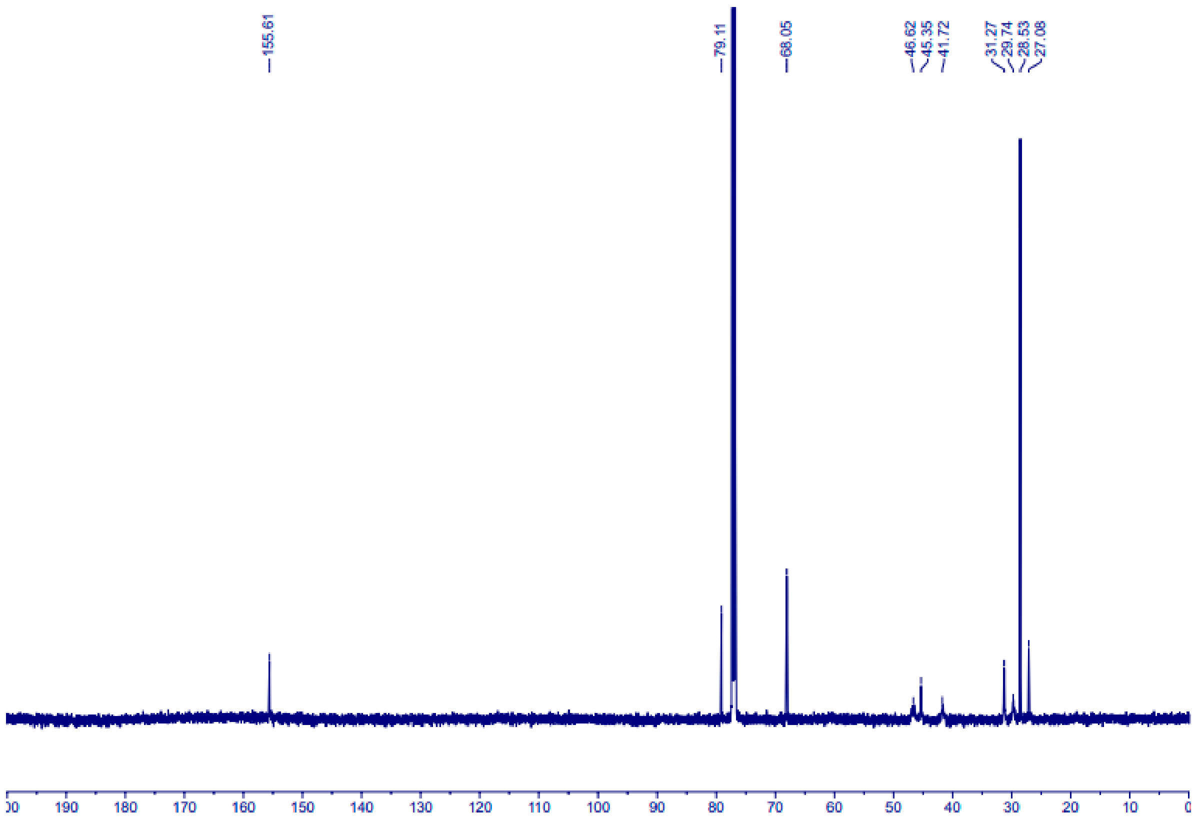

9 – ^{1}H NMR (400 MHz, $CDCl_3$)

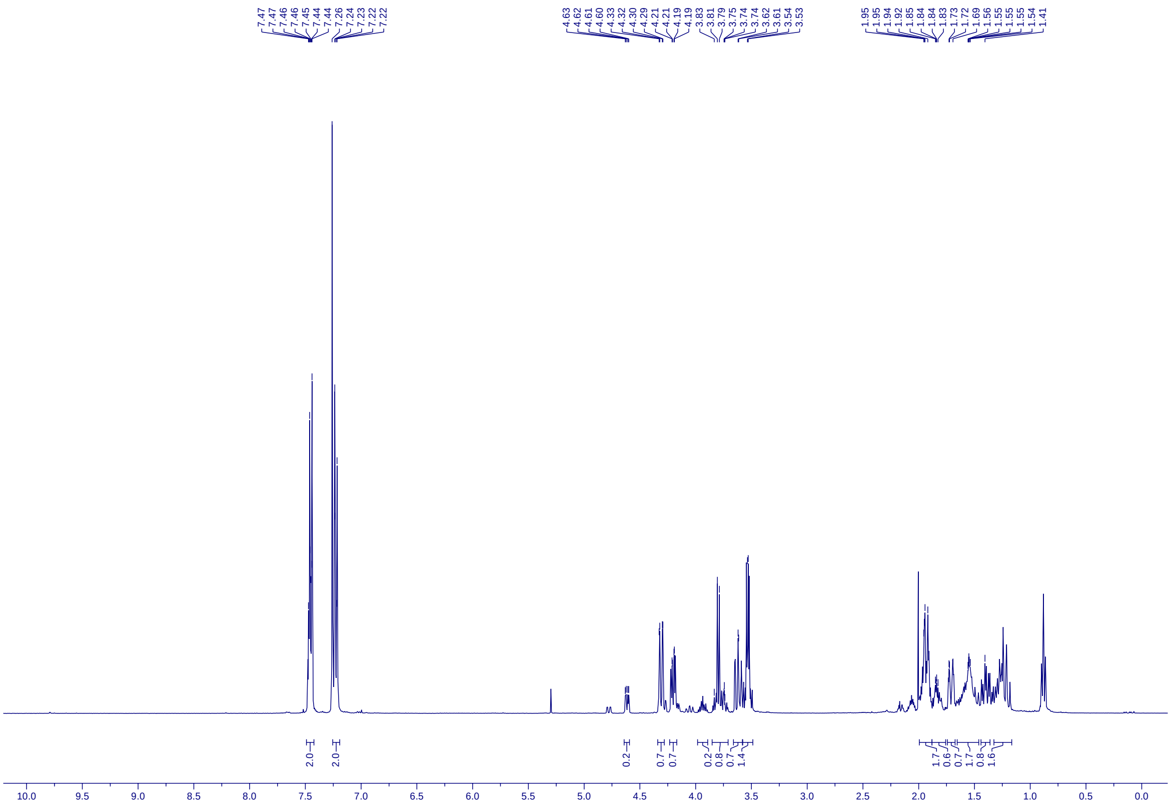

9 – ^{13}C NMR (126 MHz, $CDCl_3$)

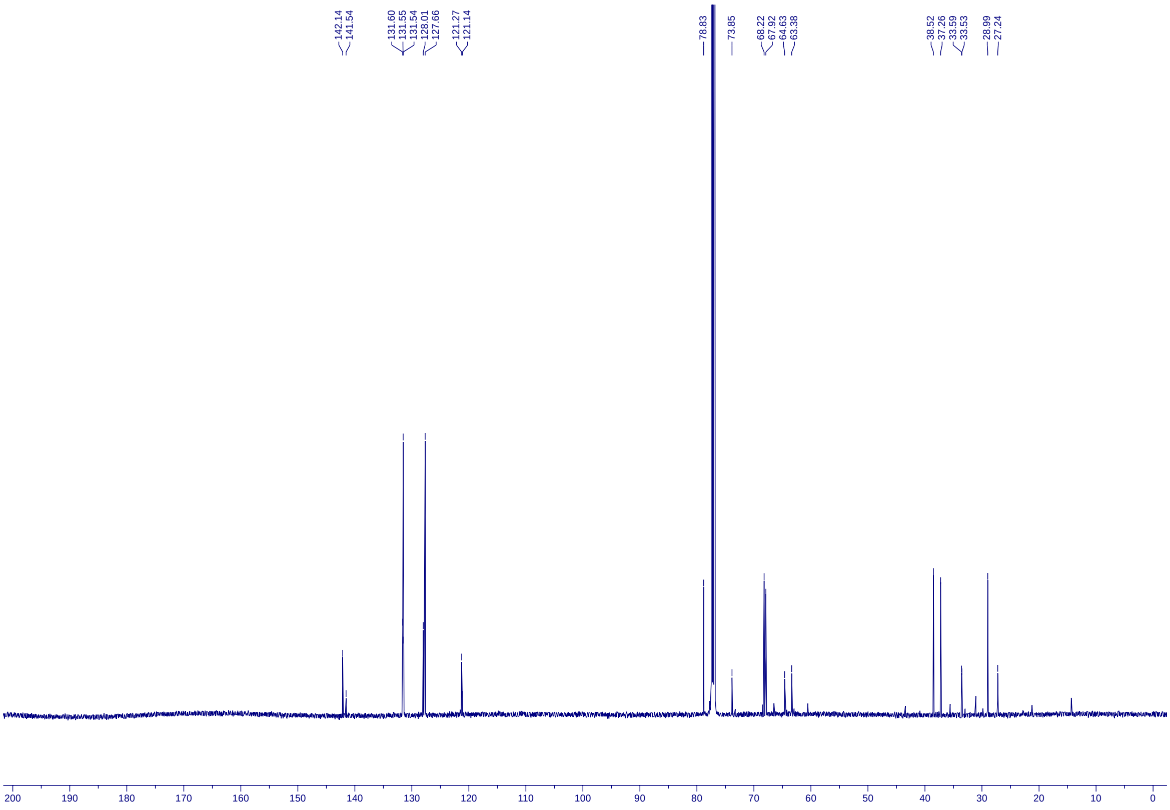

17 – ^{1}H NMR (400 MHz, $CDCl_3$)

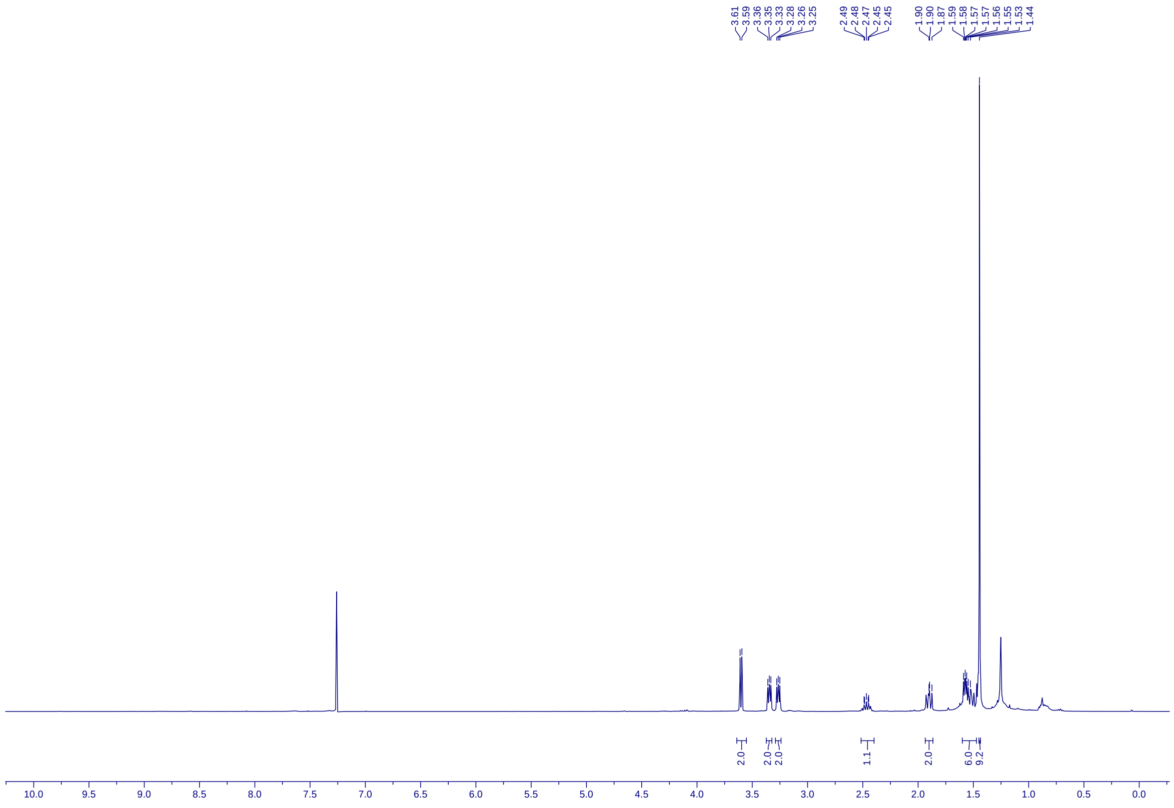

17 – ^{13}C NMR (101 MHz, $CDCl_3$)

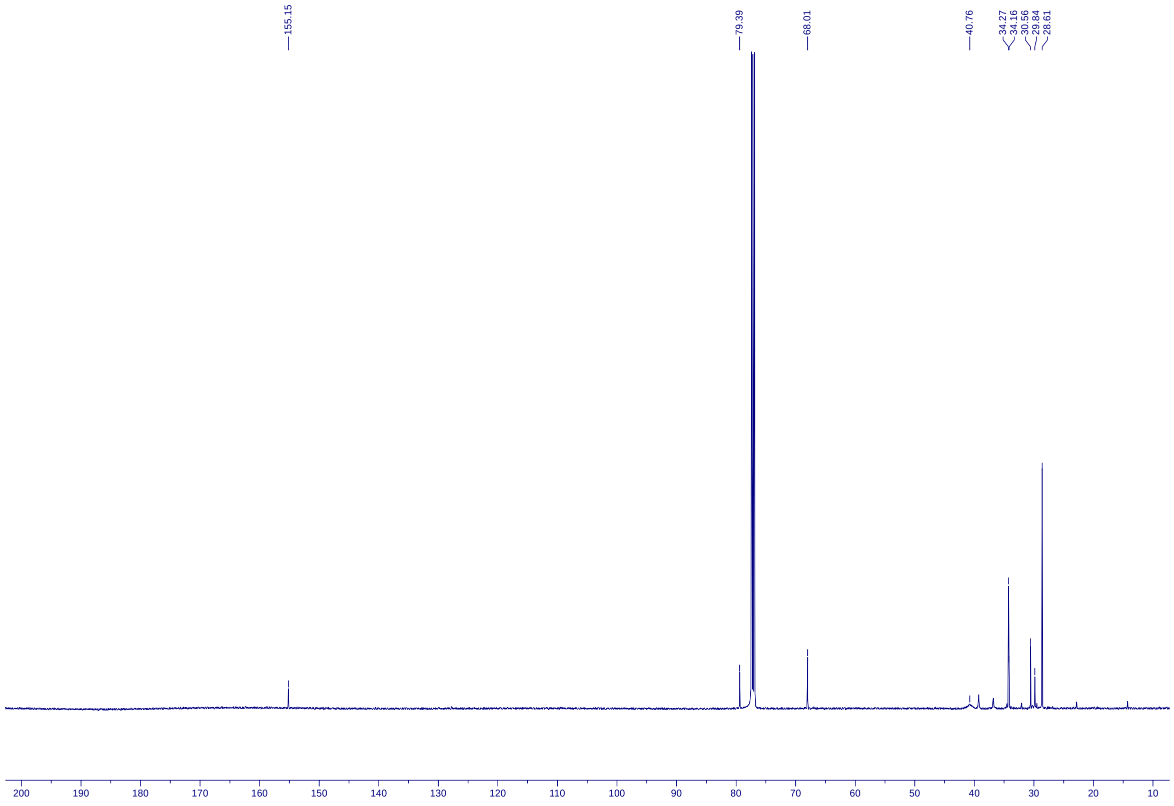

18 - ^{1}H NMR (500 MHz, CDCl$_3$)

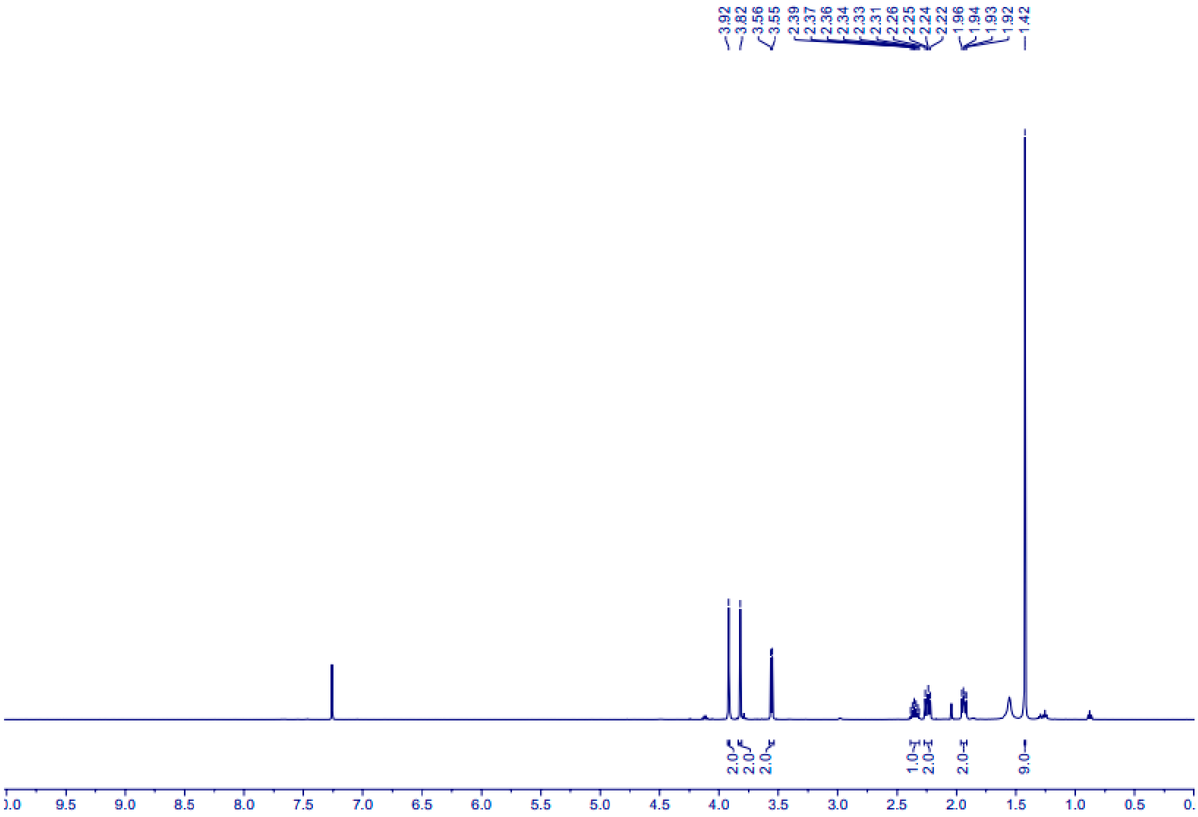

18 - ^{13}C NMR (101 MHz, CDCl$_3$)

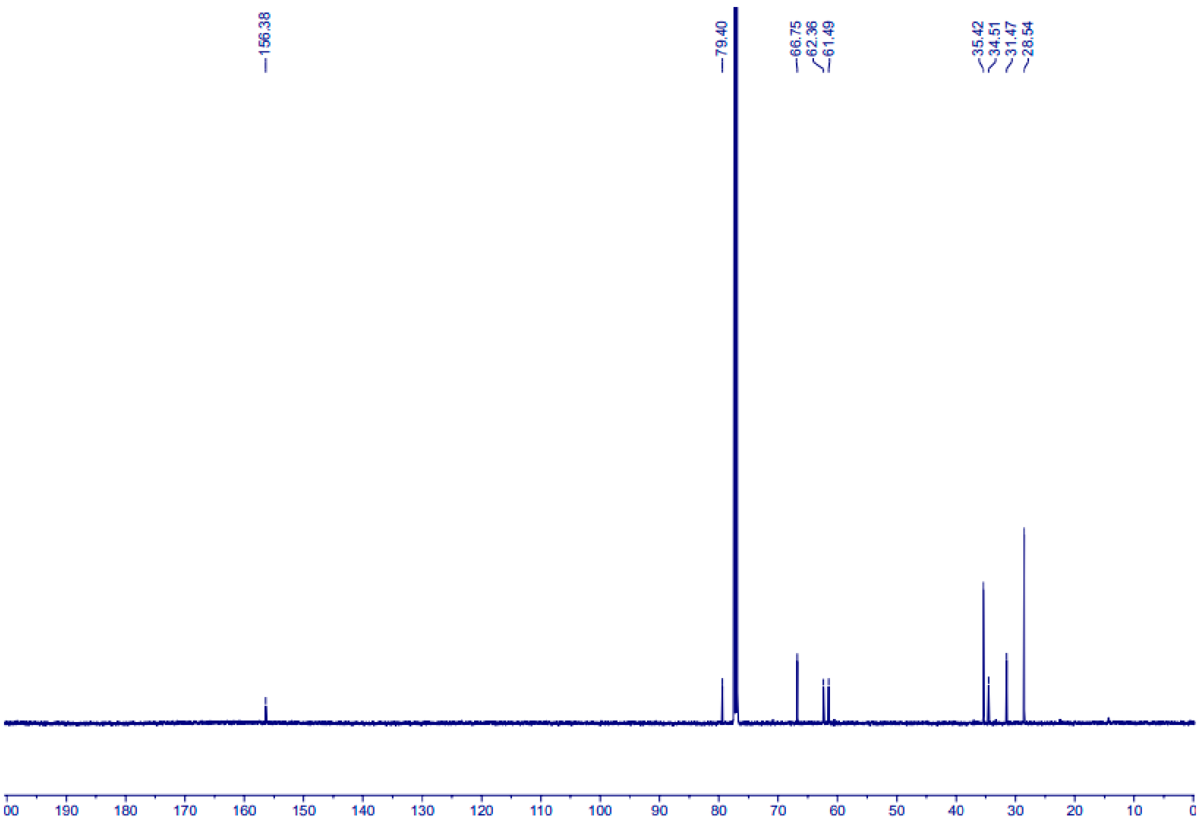

19 – ^{1}H NMR (400 MHz, $CDCl_3$)

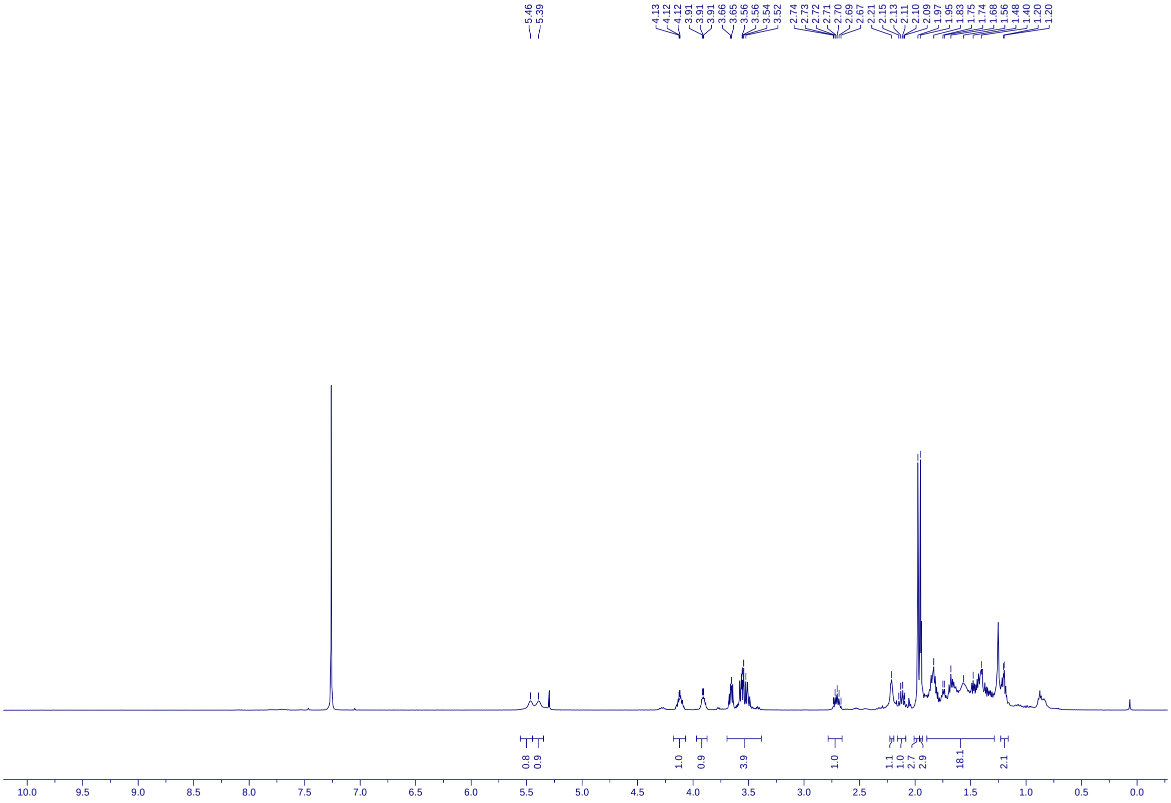

19 – ^{13}C NMR (126 MHz, $CDCl_3$)

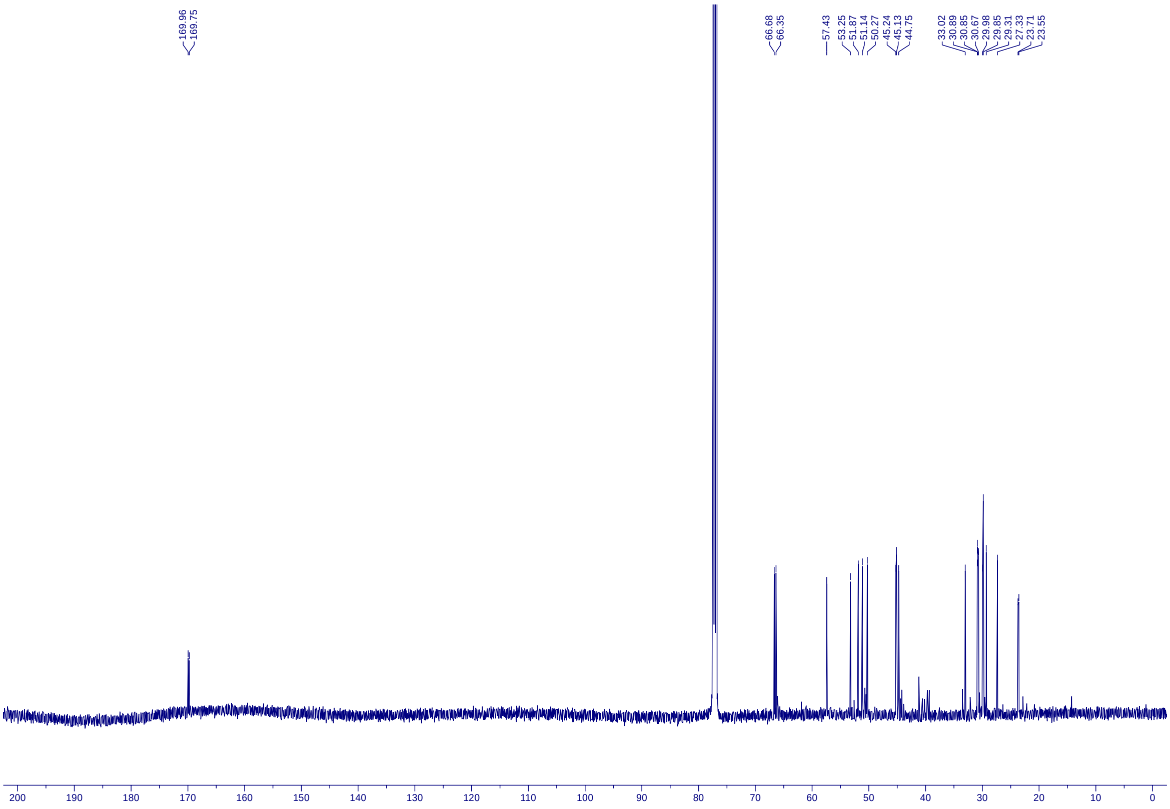

25 - ^{1}H NMR (500 MHz, $CDCl_3$)

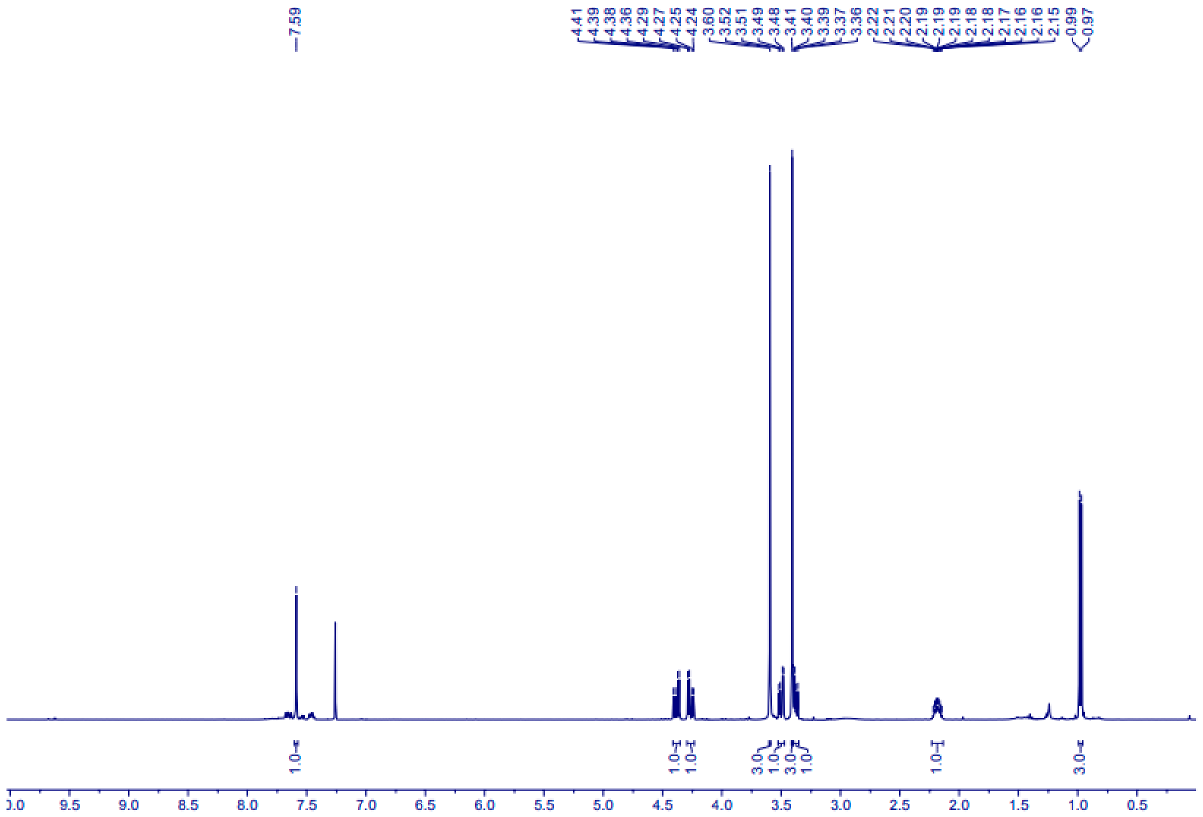

25 - ^{13}C NMR (101 MHz, $CDCl_3$)

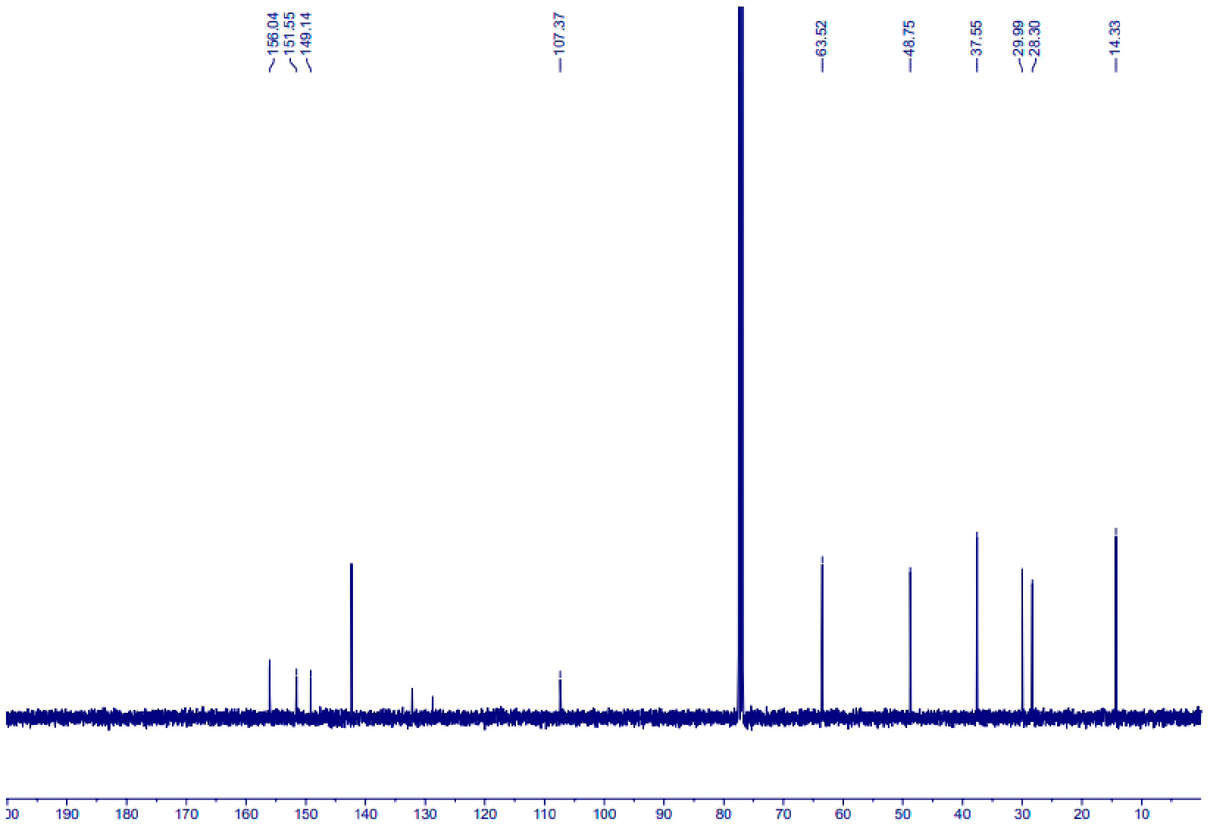

8 References

1. K. K. Schumacher, J. Jiang and M. M. Joullié, *Tetrahedron: Asymmetry*, 1998, **9**, 47-53.
2. J. Marco-Contelles and M. Álvarez-Pérez, *Synthesis*, 2009, **2009**, 3649-3653.
3. M. Tissot, N. Body, S. Petit, J. Claessens, C. Genicot and P. Pasau, *Org. Lett.*, 2018, **20**, 8022-8025.
4. S. Kobayashi, T. Kawamoto, S. Uehara, T. Fukuyama and I. Ryu, *Org. Lett.*, 2010, **12**, 1548-1551.
5. S. A. K., B. Somasekhar, I. Kiran and G. Paramartha, *Chemistry Letters*, 2011, **40**, 1176-1178.
6. S. Uemura, S. Fukuzawa, A. Toshimitsu, M. Okano, H. Tezuka and S. Sawada, *J. Org. Chem.*, 1983, **48**, 270-273.
7. C. H. Basch, J. Liao, J. Xu, J. J. Piane and M. P. Watson, *J. Am. Chem. Soc.*, 2017, **139**, 5313-5316.
8. L. Chenneberg, A. Baralle, M. Daniel, L. Fensterbank, J.-P. Goddard and C. Ollivier, *Advanced Synthesis & Catalysis*, 2014, **356**, 2756-2762.
9. R. Ishimatsu, S. Matsunami, K. Shizu, C. Adachi, K. Nakano and T. Imato, *The Journal of Physical Chemistry A*, 2013, **117**, 5607-5612.
10. E. D. Nacsa and D. W. C. MacMillan, *J. Am. Chem. Soc.*, 2018, **140**, 3322-3330.
11. H. J. Kuhn, S. E. Braslavsky and R. Schmidt, *Pure and Applied Chemistry*, 2004, **76**, 2105-2146.
12. T. Constantin, M. Zanini, A. Regni, N. S. Sheikh, F. Juliá and D. Leonori, *Science*, 2020, **367**, 1021-1026.
13. V. V. Pavlishchuk and A. W. Addison, *Inorganica Chimica Acta*, 2000, **298**, 97-102.
14. H. G. Roth, N. A. Romero and D. A. Nicewicz, *Synlett*, 2016, **27**, 714.
15. J. Lalut, G. Santoni, D. Karila, C. Lecoutey, A. Davis, F. Nachon, I. Silman, J. Sussman, M. Weik, T. Maurice, P. Dallemagne and C. Rochais, *European Journal of Medicinal Chemistry*, 2019, **162**, 234-248.
16. H. Toya, K. Okano, K. Takasu, M. Ihara, A. Takahashi, H. Tanaka and H. Tokuyama, *Org. Lett.*, 2010, **12**, 5196-5199.
17. L. Wu, I. Fleischer, R. Jackstell, I. Profir, R. Franke and M. Beller, *J. Am. Chem. Soc.*, 2013, **135**, 14306-14312.
18. Q. Wang, N. AndréSasaki and P. Potier, *Tetrahedron*, 1998, **54**, 15759-15780.
19. R. Pertschi, J.-M. Weibel, P. Pale and A. Blanc, *Org. Lett.*, 2019, **21**, 5616-5620.

20. D. Y. Ong, Z. Yen, A. Yoshii, J. Revillo Imbernon, R. Takita and S. Chiba, *Angew. Chem., Int. Ed.*, 2019, **58**, 4992-4997.

21. Y. Chen, M. Leonardi, P. Dingwall, R. Labes, P. Pasau, D. C. Blakemore and S. V. Ley, *J. Org. Chem.*, 2018, **83**, 15558-15568.

22. I. Wauters, A. De Blieck, K. Muylaert, T. S. A. Heugebaert and C. V. Stevens, *European Journal of Organic Chemistry*, 2014, **2014**, 1296-1304.

23. B.-T. Xin, G. de Bruin, E. M. Huber, A. Besse, B. I. Florea, D. V. Filippov, G. A. van der Marel, A. F. Kisselev, M. van der Stelt, C. Driessen, M. Groll and H. S. Overkleeft, *Journal of Medicinal Chemistry*, 2016, **59**, 7177-7187.

24. T. Kawamoto, T. Okada, D. P. Curran and I. Ryu, *Org. Lett.*, 2013, **15**, 2144-2147.

25. K. A. Leonard, L. A. Madge, P. J. Krawczuk, A. Wang, K. D. Kreutter, G. M. Bacani, W. Chai, R. C. Smith, M. S. Tichenor, M. C. Harris, R. Malaviya, M. Seierstad, M. E. Johnson, J. D. Venable, S. Kim, G. C. Hirst, A. S. Mathur, T. S. Rao, J. P. Edwards, M. C. Rizzolio and T. Koudriakova, *Journal of Medicinal Chemistry*, 2020, **63**, 2915-2929.

26. *WO2019144912A1*.

27. *WO2020068867*.

28. T. D. Downes, S. P. Jones, H. F. Klein, M. C. Wheldon, M. Atobe, P. S. Bond, J. D. Firth, N. S. Chan, L. Waddelove, R. E. Hubbard, D. C. Blakemore, C. De Fusco, S. D. Roughley, L. R. Vidler, M. A. Whatton, A. J.-A. Woolford, G. L. Wrigley and P. O'Brien, *Chemistry – A European Journal*, 2020, **26**, 8969-8975.

29. A. J. Grenning and F. Emmetiere, *Synthesis*, 2020, DOI: 10.1055/s-0040-1707184.

30. R. G. Parr and W. Yang, *Density-Functional Theory of Atoms and Molecules*, 1989, Oxford University Press, Oxford U.K.

31. M. J. Frisch, G. W. Trucks, H. B. Schlegel, G. E. Scuseria, M. A. Robb, J. R. Cheeseman, G. Scalmani, V. BaronE, B. Mennucci, G. A. Petersson, H. Nakatsuji, M. Caricato, X. Li, H. P. Hratchian, A. F. Izmaylov, J. Bloino, G. Zheng, J. L. Sonnenberg, M. Hada, M. Ehara, K. Toyota, R. Fukuda, J. Hasegawa, M. Ishida, T. Nakajima, Y. Honda, O. Kitao, H. Nakai, T. Vreven, J. A. M. Jr., J. E. Peralta, F. Ogliaor, M. Bearpark, J. J. Heyd, E. Brothers, K. N. Kudin, V. N. taroverov, T. Keith, R. Kobayashi, J. Normand, K. Raghavachari, A. Rendell, J. C. Burant, S. S. Iyengar, J. Tomasi, M. CossI, N. Rega, J. M. Millam, M. Klene, J. E. Knox, J. B. CrossI, V. Bakken, C. Adamo, J. Jaramillo, R. Gomperts, R. E. Stratmann, O. Yazyev, A. J. Austin, R. Cammi, C. Pomelli, J. W. Ochterski, R. L. Martin, K. Morokuma, V. G.

Zakrzewski, G. A. Voth, P. Salvador, J. J. Dannenberg, S. Dapprich, A. D. Daniels, O. Farkas, J. B. Foresman, J. V. Ortiz, J. Cioslowski and D. J. Fox, *Gaussian 09*, 2013, revision D.01; Gaussian, Inc.

32. (a) P. J. Stephens, F. J. Devlin, C. F. Chabalowski and M. J. Frisch, *J. Chem. Phys.*, 1994, **98**, 11623; (b) A. D. Becke, *J. Chem. Phys.*, 1993, **98**, 1372; (c) A. D. Becke, *J. Phys. Chem.*, 1993, **98**, 5648; (d) C. Lee, W. Yang and R. G. Parr, *Phys. Rev. B*, 1988, **37**, 785.
33. (a) S. Grimme, S. Ehrlich and L. Goerigk, *Journal of Computational Chemistry*, 2011, **32**, 1456-1465; (b) S. Grimme, J. Antony, S. Ehrlich and H. Krieg, *The Journal of Chemical Physics*, 2010, **132**, 154104.
34. F. Weigend and R. Ahlrichs, *Physical Chemistry Chemical Physics*, 2005, **7**, 3297-3305.
35. (a) J. Tomasi, B. Mennucci and R. Cammi, *Chemical Reviews*, 2005, **105**, 2999-3094; (b) B. Mennucci, E. Cancès and J. Tomasi, *The Journal of Physical Chemistry B*, 1997, **101**, 10506-10517.
36. (a) A. D. Becke, *The Journal of Chemical Physics*, 1993, **98**, 5648-5652; (b) P. J. Stephens, F. J. Devlin, C. F. Chabalowski and M. J. Frisch, *J. Phys. Chem.*, 1994, **98**, 1372.
37. F. D. Vleeschouwer, V. V. Speybroeck, M. Waroquier, P. Geerlings and F. D. Proft, *Org. Lett.*, 2007, **9**, 2721-2724.
38. F. L. Hirshfeld, *Theoret. Chim. Acta*, 1977, **44**, 129.
